# Relationships among gut microbes, the interleukin family, and hypertension: a mediation Mendelian randomization study

**DOI:** 10.3389/fnut.2023.1293170

**Published:** 2023-11-28

**Authors:** Shiyao Zhao, Jing Zhang, Fuxia Ding, Shougang Sun

**Affiliations:** Department of Cardiology, Lanzhou University Second Hospital, Lanzhou, Gansu, China

**Keywords:** gut microbiome, hypertension, Mendelian randomization, interleukin, mediation

## Abstract

**Purpose:**

Observational studies have increasingly recognized the influence of gut microbes on blood pressure modulation. Despite these findings, a direct causal link between gut flora and hypertension remains unestablished due to inherent confounders and the challenges of reverse causality in observational research. In this study, we sought to elucidate the causal relationship between specific gut flora and hypertension and its intermediary mediators.

**Methods:**

We employed a two-sample Mendelian randomization (MR) and mediation MR analysis, analyzing 211 species of gut bacteria, with a focus on the interleukin family as potential mediators and hypertension as the primary outcome. The central methodological technique was inverse variance-weighted estimation, supplemented by various other estimators.

**Results:**

Our findings revealed that two bacterial species positively correlated with hypertension risk, while five exhibited a negative association. Further validation was conducted using sensitivity analyses. Notably, our mediation MR results suggest interleukin-1 receptor type 2 (IL-1R2) as a mediator for the effect of the genus *Clostridium innocuum group* on hypertension, accounting for a mediation proportion of 14.07% [mediation effect: (*b* = 0.0007, 95%CI: 0.0002–0.0011); proportion mediation = 14.07% (4.26–23.40%)].

**Conclusion:**

Our research confirms a genetic causal relationship between specific gut microbes and hypertension, emphasizing the potential mediating role of interleukin-1 receptor type 2 (IL-1R2) and offering insights for clinical hypertension interventions.

## 1 Introduction

Globally, hypertension is an increasing health concern, affecting over 1.5 billion individuals (1). This condition is intrinsically linked with CVD (cardiovascular disease), cerebrovascular diseases, and kidney diseases (2–4). Notably, a 2 mmHg reduction in blood pressure can lead to a 7% decrease in cardiovascular disease mortality and a 10% decline in stroke mortality (5). While the interplay of genetic and environmental influences contributes to the onset of hypertension (6–8), its precise pathogenesis remains elusive.

The human gastrointestinal system houses a vast array of microorganisms, of which the intestinal bacteria play an indispensable role in maintaining ecological balance (9). Not only is it pivotal for digesting foods and aiding nutrient absorption, supplying energy for daily metabolic processes, but it also performs a vital endocrine function by secreting substances that partake in various physiological processes (10). Recent research has highlighted a potential connection between hypertension and the gut microbiota (11, 12). For instance, Li et al. showed a reduced abundance of gut microbiota in hypertensive individuals compared to healthy ones (12). Additionally, after transplanting fecal matter from hypertensive patients into germ-free mice, an elevation in blood pressure was observed after 10 weeks (12). While animal models provide valuable insights, they cannot entirely replace clinical studies. Exploring the causal relationship between hypertension and gut flora in clinical studies poses challenges, leading to the paucity of such studies. Currently, the number of clinical studies investigating the relationship between gut microbes and hypertension is limited, and those available often feature small sample sizes. Several factors contribute to this complexity. The primary pathophysiological mechanisms might be masked by compensatory pathways, and long-term dietary habits have a pronounced effect on gut flora. This is evident in studies examining the relationship between hypertension and *Clostridium difficile*, where divergent findings are presented (13, 14). It is also possible that the observed intestinal ecological disturbances are merely symptoms accompanying hypertension. The existing links between hypertension and gut flora largely stem from observational studies with limited sample sizes, tainted by numerous confounding variables. The nature of these studies makes it difficult to exclude the possibility of reverse causation, thereby compromising their reliability in establishing causality. In essence, while a relationship between hypertension and enterobacteria is evident, the causal links remain ambiguous, demanding further research to elucidate the intricate pathophysiological mechanism.

Mendelian randomization (MR) is a research technique grounded in Mendel's second law that leverages the random assignment of alleles from parents to offspring during gamete formation (15, 16). This method uses genotypes, which determine intermediate phenotypes like exposure traits, to draw associations with disease outcomes (15, 16). Because of this natural randomness in allele assignment, MR inherently mitigates the effects of confounding environmental factors and precludes reverse causation (15, 16). As a result, MR offers a robust mechanism for deriving causal inferences from observational data. Therefore, in this study, we employed the MR approach to probe the causal relationship between hypertension and gut flora.

## 2 Methods and materials

### 2.1 Study design

**Figure 1:**
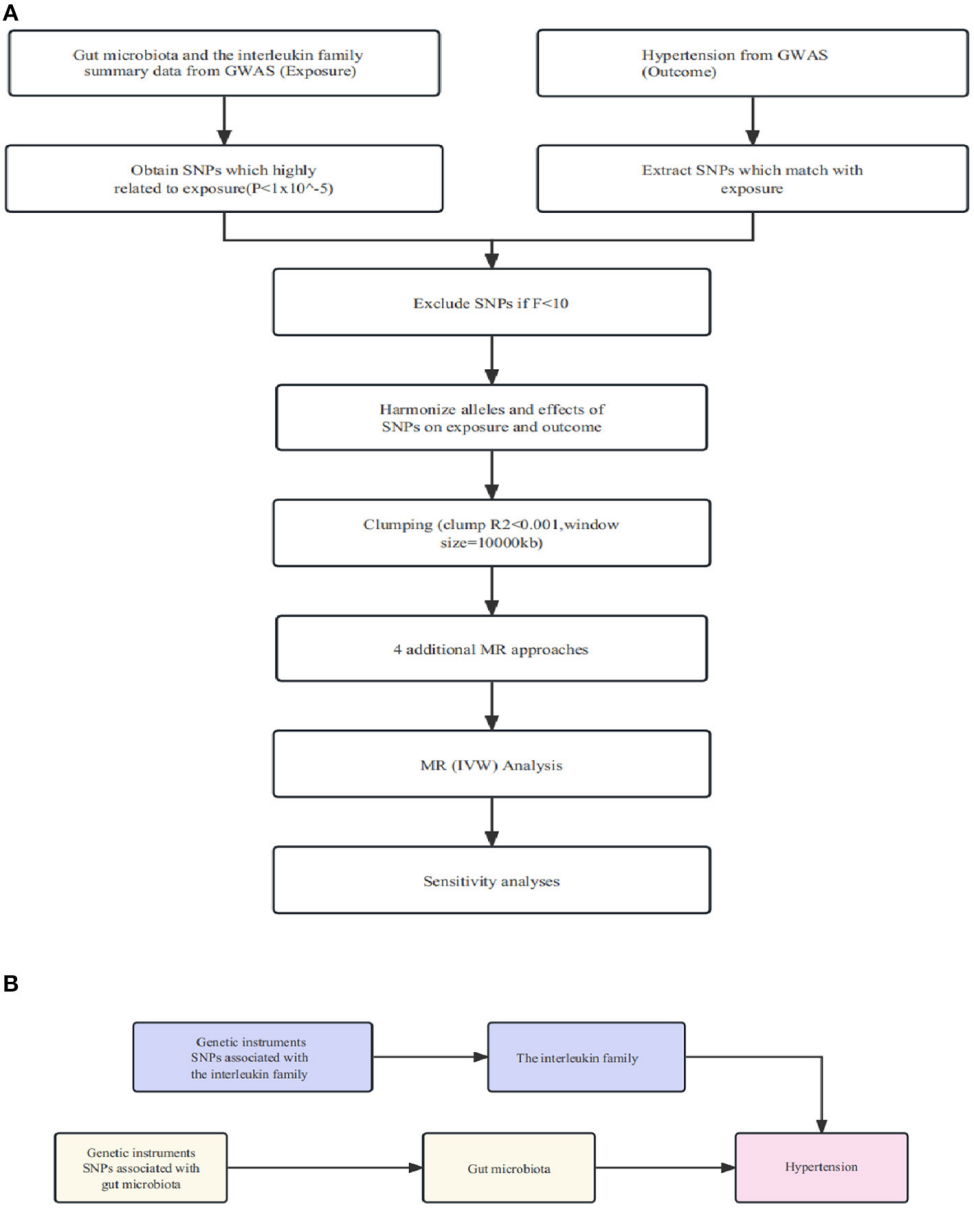
**(A)** Illustration of the study design and workflow. **(B)** Two-step Mendelian randomization assessment detailing the impact of gut microbiota on hypertension through IL-1R2.

In order to obtain reliable results, a two-sample MR should satisfy three essential assumptions: (1) Instrumental variables (IVs) must be significantly associated with gut microorganisms; (2) IVs are not associated with other factors that could confound the relationship between exposure and outcome variables; (3) IVs should influence outcomes solely via the gut microorganisms. As depicted in the flowchart (Figure 1), our study first employed a two-sample MR to establish the causal relationship between specific gut flora and interleukin (IL)-related factors in relation to hypertension. Following this, we utilized a mediated MR (two-step MR) to delve into the causal link between specific gut flora, as mediated by interleukin-related factors, and hypertension.

### 2.2 Data sources

For exposure variables, we sourced summary statistics on gut microorganisms from a comprehensive, multi-ethnic GWAS meta-analysis involving 340,024 participants from 18 cohorts. This analysis comprised 211 taxa, including 131 genera, 35 families, 20 orders, 16 classes, and 9 phyla (17).

For mediator and outcome phenotypes, we relied on the data of the MRC-IEU consortium [IEU OpenGWAS project (mrcieu.ac.uk)]. The study populations for these phenotypes were primarily of European descent (18). Specific data IDs can be referenced in Supplementary material S1.

### 2.3 Selection for genetic variation

Utilizing Plink Software, we first screened for single nucleotide polymorphisms (SNPs) from GWAS of gut microbiota with *P* < 1^*^10^−5^, a genetic distance of 10,000 kb, and a linkage disequilibrium parameter (*r*^2^) of <0.001. When the identified SNPs were absent in the outcomes GWAS summary data, we substituted them with proxies boasting an *r*^2^ ≥ 0.8. Subsequently, we computed the *F* statistic for each SNP to detect any weak IV bias (19). SNPs with an F statistic of <10 indicated potential weak IV bias, and to ensure accurate results, these were excluded from the study (20).

### 2.4 Statistical analysis

While the core focus of this study was on the results derived from the inverse variance weighting (IVW) method (19), it was essential to ensure the directional consistency of results across all utilized methods with the IVW. For a more rigorous secondary analysis, we employed MR-Egger regression, weighted median, simple mode, weighted mode, and MR-Pleiotropy Residual Sum and Outlier (MR-PRESSO) (15, 21–23) to provide a sensitivity examination for our IVW findings.

### 2.5 Pleiotropy and heterogeneity analysis

We initiated our analysis using the MR-PRESSO (23) approach to identify outliers and proceeded with a re-analysis after their exclusion. Subsequently, to assess any individual SNP's potential to drive the association between exposure and outcome, a leave-one-out sensitivity analysis was performed by iteratively removing one SNP at a time. Furthermore, to discern horizontal pleiotropy in MR analysis, the MR-Egger regression test was employed, emphasizing the statistical significance of the intercept term (24). Finally, we computed the Cochran Q statistic to detect heterogeneity (25), setting the significance of the threshold at *P* = 0.05. All statistical analyses were conducted in R, version 4.2.3, with the MR and MR-PRESSO packages.

## 3 Results

### 3.1 Instrument variables included in the analysis

Detailed information on the SNPs available for our study can be found in Supplementary Figure S2. All SNPs included in our analysis possessed an F-value exceeding 10.

### 3.2 Effects of microbial taxa on hypertension

**Figure 2:**
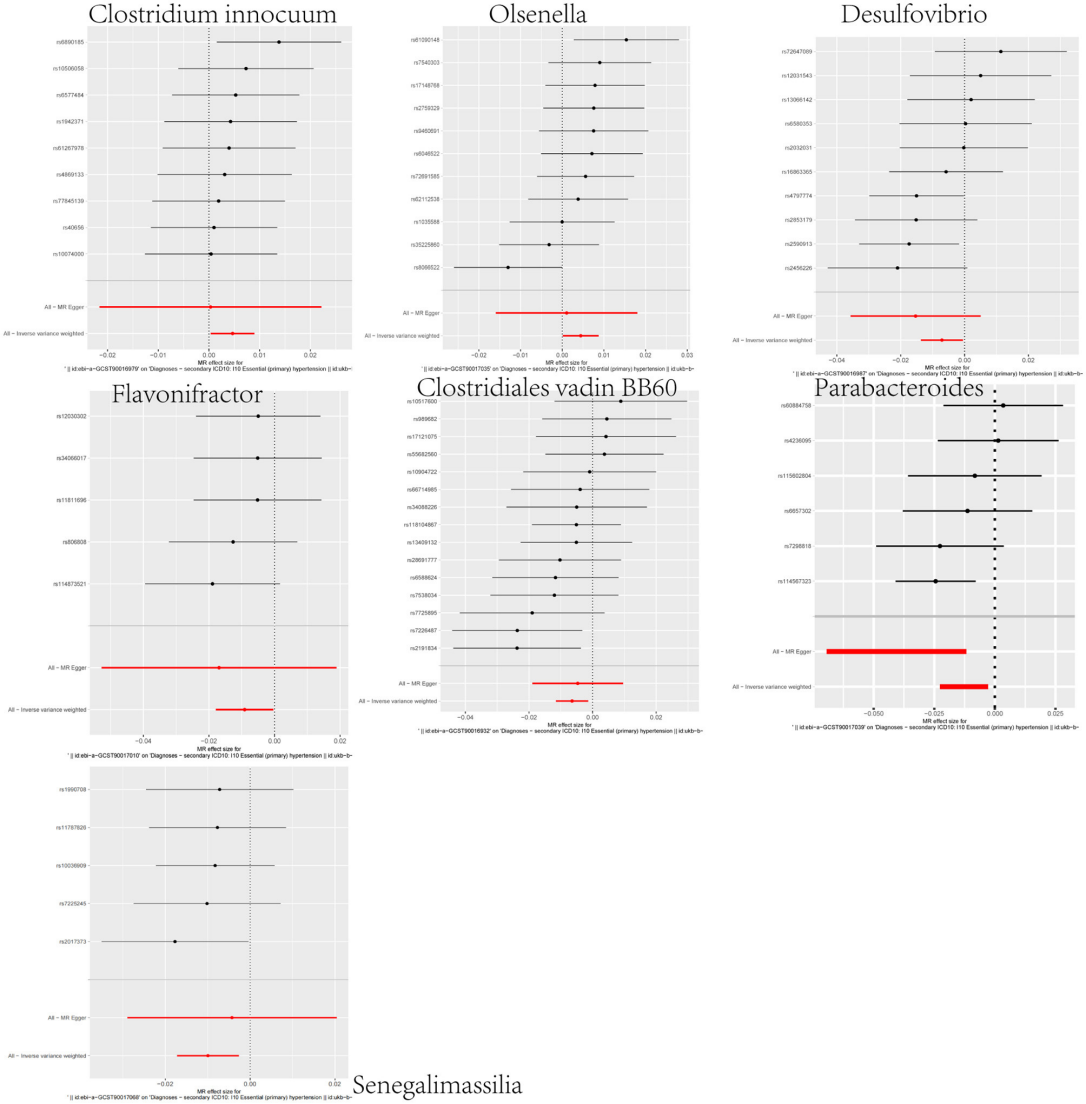
Forest plot representing the causal relationship between gut microbiota and hypertension.

In our investigation of the effects of microbial taxa on hypertension, we identified a positive association between hypertension and two gut microbiota taxa—the *genus Clostridium innocuum group* (OR = 1.0047, 95% CI: 1.0004–1.0090, *p* = 0.0336) and the *genus Olsenella* (OR = 1.0044, 95%CI: 1.0001–1.0088, *p* = 0.0430). The results suggest their potential to heighten hypertension risk (Figure 2; Supplementary Figures S3–S5). Conversely, five gut microbiota taxa, including the *genus Senegalimassilia* (OR = 0.9901, 95%CI: 0.9830–0.9973, *p* = 0.0072), the *genus Parabacteroides* (OR = 0.9874, 95%CI: 0.9776–0.9972, *p* = 0.0121), the *Clostridiales vadin BB60 group family* (OR = 0.9936, 95%CI: 0.9886–0.9986, *p* = 0.0125), the *genus Desulfovibrio* (OR = 0.9929, 95%CI: 0.9864–0.9995, *p* = 0.0342), and the *genus Flavonifractor* (OR = 0.9909, 95%CI: 0.9823–0.9997, *p* = 0.0416), were linked to a diminished hypertension risk, suggesting their possible antihypertensive properties (Figure 2; Supplementary Figures S3–S5). To validate the aforementioned results, we conducted a sensitivity analysis using the MR-Egger, weighted median, simple mode, and weighted mode methods (Supplementary Figure S6).

### 3.3 Effects of interleukin on hypertension

**Figure 3:**
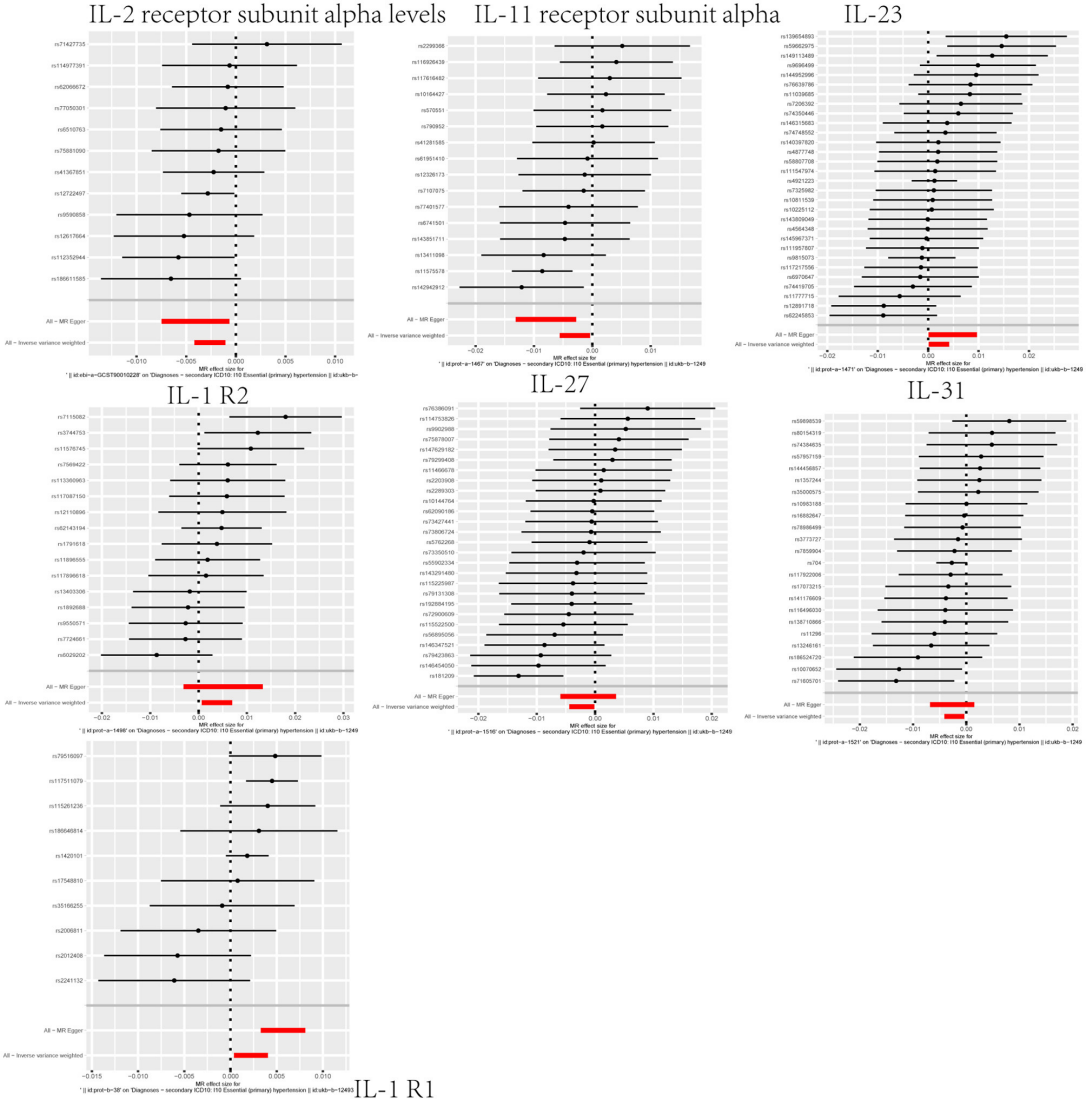
Forest plot highlighting the causal link between the interleukin family and hypertension.

Through our MR analysis, we identified correlations between interleukin-related factors and hypertension risk. Specifically, three factors, namely, IL-1R2 (OR = 1.0038, 95%CI: 1.0007–1.0070, *p* = 0.0180), interleukin-23 (OR = 1.0021, 95%CI: 1.0001–1.0042, *p* = 0.0381), and interleukin-1 receptor-like 1 (OR = 1.0022, 95%CI: 1.0004–1.0041, *p* = 0.0176), displayed a positive association, suggesting they may heighten the risk of hypertension (Figure 3; Supplementary Figures S7–S9). Four factors, namely, interleukin-2 receptor subunit alpha levels (OR = 0.9974, 95%CI: 0.9958–0.9989, *p* = 0.0010), interleukin-11 receptor subunit alpha (OR = 0.9970, 95%CI: 0.9944–0.9996, *p* = 0.0250), interleukin-27 (OR = 0.9977, 95%CI: 0.9956–0.9999, *p* = 0.0385), and interleukin-31 (OR = 0.9978, 95%CI: 0.9959–0.9996, *p* = 0.0189), were inversely associated, indicating a potential protective effect against hypertension (Figure 3; Supplementary Figures S7–S9). We additionally performed a sensitivity analysis to corroborate this result (Supplementary Figure S10).

### 3.4 Effects of microbial taxa on IL-1R2

**Figure 4:**
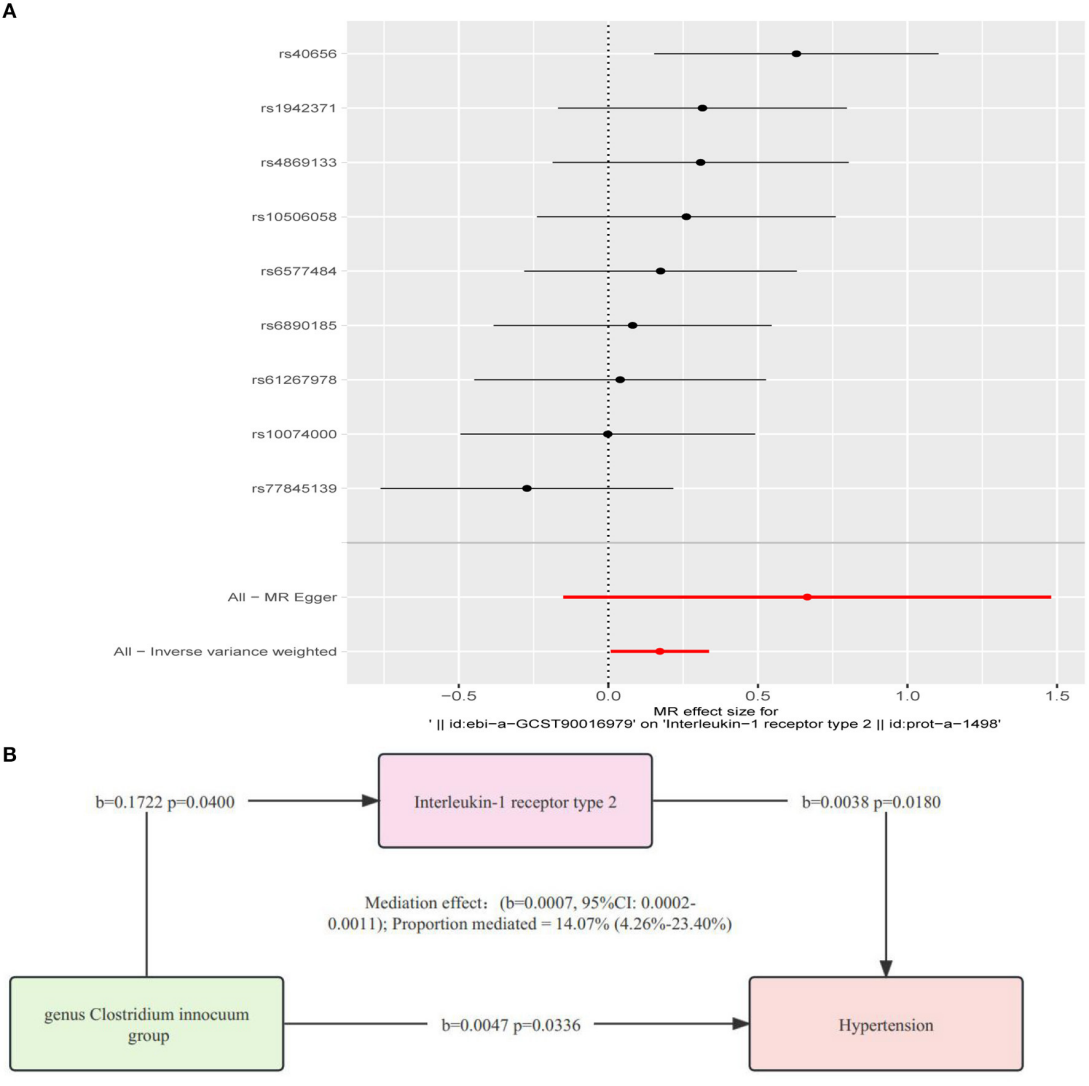
**(A)** Analysis of the causative relationship from *Clostridium innocuum* group to IL-1R2 (*P* < 1.00E-05). **(B)** Depiction of the role of interleukin-1 receptor type 2 in mediating the causal effect of gut microbiota on hypertension, with MR estimates for the connection among interleukin-1 receptor type 2, gut microbiome, and hypertension provided via the inverse variance-weighted approach.

The results revealed a causal effect of the genetically predicted *Clostridium innocuum group* (OR = 1.1879, 95%CI: 1.0079–1.4001, *p* = 0.0400) on IL-1R2 as indicated by the IVW method (Figure 4; Supplementary Figures S11–S13). Given our earlier findings that established connections between “microbial taxa → hypertension” and “microbial taxa → IL-1R2”, we postulated that IL-1R2 may potentially mediate the relationship between microbial taxa and hypertension. We also conducted a sensitivity analysis to reinforce this conclusion (Supplementary Figure S14).

### 3.5 The mediation effect of IL-1R2 in the causal association between the *Clostridium innocuum* group and hypertension

Using the two-step IL-1R2 method, we determined that IL-1R2 acts as a mediator in the causal relationship from the *Clostridium innocuum group* to hypertension (*b* = 0.0007, 95%CI: 0.0002–0.0011). The mediation effect was quantified at 14.07% (4.26–23.40%) (Figure 4).

## 4 Discussion

In this comprehensive mediation MR study, we identified a causal relationship between seven microbial taxa (including the *Clostridium innocuum group*) and hypertension risk. Mediation MR results suggest that IL-1R2 may account for 14.07% of the effect that the *Clostridium innocuum group* has on hypertension. This analysis underscores the link between gut microbiota and hypertension, highlighting the mediating role of IL-1R2.

Gut flora modulates the immune response in two primary ways: direct immune cell recognition, triggering an immune response, and regulation through metabolites like short-chain fatty acids (SCFAs) (26, 27). These SCFAs, such as butyric acid, influence the immune response not only in the intestines but also in the heart, brain, and other mucosal sites (28, 29). Notably, the intestinal bacteria *Faecalibacterium, Ruminococcaceae, Coprococcus*, and *Roseburia* are known producers of butyric acid (30, 31). A broader array, including the *Parabacteroides* and *Clostridiales vadin BB60 group* highlighted in our findings, produces other SCFAs such as acetate, propionate, and hexanoate (32, 33). The protective attributes of the *Parabacteroides* and the *Clostridiales vadin BB60 group* observed in our results may be attributed to their SCFA production. In addition, Shiyun Luo et al. found the protective role of *Flavonifractor* against atherosclerosis, suggesting a potential link between its observed hypertension protection in our study and its anti-atherosclerosis effects.

There is a prevailing consensus from animal model studies that the intestinal flora has a robust association with hypertension (34–38). While research has identified a positive relationship between certain bacterial strains such as *Klebsiella, Anaerotruncus*, and *Eggerthella* and hypertension and a negative relationship between bacterial strains such as *Roseburia* and *Bifidobacterium* and hypertension, the exact causality remains elusive (12, 14, 39–42). Emerging insights point to neuroinflammation as a potential intermediary in the influence of gut flora on hypertension. For instance, hypertensive mice introduced to standard mouse gut flora exhibited reduced neuroinflammation in the hypothalamic paraventricular nucleus and notably decreased blood pressure (43). Furthermore, in angiotensin II-induced hypertensive mice, anti-inflammatory treatments managed to restore their gut microbial balance (44). Wang et al. proposed a bi-directional regulation of gut flora and hypertension via both humoral and neural mechanisms (45), albeit with sparse evidence. Additionally, Santisteban et al. observed that an imbalanced gut flora and its metabolites could stimulate macrophages to release copious inflammatory factors, subsequently elevating blood pressure (46). Despite the limited direct evidence linking inflammation-mediated gut flora to hypertension, these findings illuminate promising avenues for future hypertension research.

Members of the IL-1 family or their receptors serve as therapeutic targets for several conditions, including atherosclerosis, type 2 diabetes, arthritis, and cancer (47, 48). IL-1R2, a primary IL-1 binding protein found in human neutrophils, monocytes, and B cells, plays a crucial role in regulating IL-1-mediated inflammatory responses (49–52). It also acts as a decoy receptor for the IL-1 system (49). Elevated IL-1R2 expression has been observed in various cancers, such as pancreatic ductal adenocarcinoma (53), prostate cancer, and benign prostatic hyperplasia (54). Additionally, Pou et al. established a connection between IL-1R2 and atherosclerotic vasculopathy (55). While the link between hypertension and inflammation is well acknowledged, to the best of our knowledge, no current studies delve into the relationship between IL-1R2 and hypertension. Our findings indicate that IL-1R2 could bridge the causal link between intestinal microorganisms and hypertension, laying the groundwork for exploring the intersection of hypertension and inflammation and potentially inspiring future therapeutic strategies for hypertension.

It is important to note that studies on differences in gut flora among hypertensive patients yield inconsistent results. For instance, both Dan et al. and Huart et al. observed a higher prevalence of *Clostridium* sensu stricto in hypertensive groups (56, 57). Verhaar et al. reported contrary findings (58). Similar inconsistencies are evident in studies exploring *Bacteroides, Clostridiales, Lactobacillaceae*, and *Oscillibacter* (12, 39, 40, 56, 59). Such discrepancies might arise from several factors: the studies might not have accounted for potential confounders such as gender, age, race, diet, and medication use. Furthermore, bacterial classifications might differ between studies. Another consideration is the possible influence of contrasting causal factors in hypertension, though their precise roles remain unclear.

We pioneered the use of mediation MR to investigate the causal relationship among the gut microbiome, inflammation, and hypertension. Not only did we utilize various common sensitivity analyses, but we also ruled out the influence of confounding factors and reverse causality. Our preliminary findings suggest a causal relationship between the gut microbiome and hypertension and its intermediaries, offering further theoretical support for hypertension treatment and prevention and presenting new approaches for its management. For instance, one could initially regulate specific gut microbes through diet, medication, or other means to manage hypertension. Additionally, researching targeted drugs against interleukin-related receptors might lead to significant breakthroughs in hypertension prevention and treatment.

Despite the many strengths of our study, such as a large sample size, the use of multiple sensitivity analysis methods to ensure research reliability, ruling out confounding factors, and clarifying causality, there are unavoidable limitations. Despite the significant sample size of the study, it is worth noting that participants were exclusively of European descent. Hence, our findings may not be entirely applicable to other regions or ethnicities. Furthermore, our results remain theoretical and have not been validated through clinical or animal experiments, leaving the specific mechanisms unclear. Further cellular, animal, and clinical experiments are needed to elucidate these mechanisms. Finally, our exposure data selected over 200 gut microbial species from the research of Kurilshikov et al. (17). In reality, there often exists more than one dataset for a single microbial species. We did not investigate all the data for each microbial species, which might lead to false positives or negatives or limit the applicability of our findings.

## 5 Conclusion

Our mediation MR research suggests potential causal relationships among the gut microbiome, inflammation, and hypertension. Specifically, the IL-1R2 pathway mediates a regulatory effect of the gut microbiome on blood pressure. Our findings provide genetic evidence of the connection between the gut microbiome, IL-1R2, and hypertension. This sheds light on new possibilities for hypertension prevention and treatment in the future, possibly by modulating specific gut microbial communities or interleukin-related targets.

## Data availability statement

The original contributions presented in the study are included in the article/Supplementary material, further inquiries can be directed to the corresponding author.

## Author contributions

SZ: Data curation, Formal analysis, Software, Supervision, Writing – original draft, Writing – review & editing. JZ: Conceptualization, Methodology, Writing – review & editing. FD: Supervision, Validation, Writing – review & editing. SS: Supervision, Writing – review & editing.
