# Supplementary material for: Relationships among gut microbes, the interleukin family, and hypertension: a mediation Mendelian randomization study
Source: Front Nutr. 2023 Nov 28;10:1293170. doi: 10.3389/fnut.2023.1293170 (PMC10714948; doi:10.3389/fnut.2023.1293170)
Supplement: Supplementary file 1 [file Data_Sheet_1.docx]

| data | id |
| --- | --- |
| family Clostridiales vadin BB60 group id.11286 | ebi-a-GCST90016932 |
| genus Clostridium innocuum group id.14397 | ebi-a-GCST90016979 |
| genus Desulfovibrio id.3173 | ebi-a-GCST90016987 |
| genus Flavonifractor id.2059 | ebi-a-GCST90017010 |
| genus Olsenella id.822 | ebi-a-GCST90017035 |
| genus Parabacteroides id.954 | ebi-a-GCST90017039 |
| genus Senegalimassilia id.11160 | ebi-a-GCST90017068 |
| Interleukin-2 receptor subunit alpha levels | ebi-a-GCST90010228 |
| Interleukin 1 receptor like 1 | prot-b-38 |
| Interleukin-1 receptor type 2 | prot-a-1498 |
| Interleukin-31 | prot-a-1521 |
| Interleukin-11 receptor subunit alpha | prot-a-1467 |
| Interleukin-23 | prot-a-1471 |
| Interleukin-27 | prot-a-1516 |
| hypertension | ukb-b-12493 |

**S1. The ids of the data used in this study.**

**S2. Single-nucleotide polymorphisms associated with exposure and outcome.**

| exposure | id.exposure | outcome | id.outcome | SNP | effect_allele.exposure | other_allele.exposure | effect_allele.outcome | other_allele.outcome | beta.exposure | se.exposure | pval.exposure | beta.outcome | se.outcome | pval.outcome | F |
| --- | --- | --- | --- | --- | --- | --- | --- | --- | --- | --- | --- | --- | --- | --- | --- |
| family Clostridiales vadin BB60 group id.11286 | ebi-a-GCST90016932 | hypertension | ukb-b-12493 | rs10517600 | G | T | G | T | -0.0626993 | 0.0139364 | 6.82763E-06 | -0.000556749 | 0.000667103 | 0.4 | 20.23780744 |
| family Clostridiales vadin BB60 group id.11286 | ebi-a-GCST90016932 | hypertension | ukb-b-12493 | rs10904722 | C | T | C | T | -0.0672314 | 0.0147123 | 5.04836E-06 | 6.14421E-05 | 0.000716314 | 0.93 | 20.87961308 |
| family Clostridiales vadin BB60 group id.11286 | ebi-a-GCST90016932 | hypertension | ukb-b-12493 | rs118104867 | C | T | C | T | 0.214464 | 0.0455098 | 3.43598E-06 | -0.00108933 | 0.00152926 | 0.48 | 22.2043585 |
| family Clostridiales vadin BB60 group id.11286 | ebi-a-GCST90016932 | hypertension | ukb-b-12493 | rs13409132 | A | G | A | G | -0.165419 | 0.0352154 | 4.3723E-06 | 0.000841681 | 0.00147921 | 0.57 | 22.0619965 |
| family Clostridiales vadin BB60 group id.11286 | ebi-a-GCST90016932 | hypertension | ukb-b-12493 | rs17121075 | G | A | G | A | 0.0769254 | 0.0172234 | 7.91425E-06 | 0.000325102 | 0.00086369 | 0.709999 | 19.94532105 |
| family Clostridiales vadin BB60 group id.11286 | ebi-a-GCST90016932 | hypertension | ukb-b-12493 | rs2191834 | T | G | T | G | -0.0746375 | 0.0159136 | 2.50196E-06 | 0.00177235 | 0.000763973 | 0.02 | 21.99462562 |
| family Clostridiales vadin BB60 group id.11286 | ebi-a-GCST90016932 | hypertension | ukb-b-12493 | rs28691777 | C | T | C | T | 0.137134 | 0.0266996 | 6.95697E-07 | -0.00140512 | 0.0013447 | 0.3 | 26.37667563 |
| family Clostridiales vadin BB60 group id.11286 | ebi-a-GCST90016932 | hypertension | ukb-b-12493 | rs34088226 | A | G | A | G | -0.117807 | 0.026924 | 7.66214E-06 | 0.000587516 | 0.00132623 | 0.66 | 19.14266108 |
| family Clostridiales vadin BB60 group id.11286 | ebi-a-GCST90016932 | hypertension | ukb-b-12493 | rs55682560 | C | T | C | T | -0.131519 | 0.0261319 | 4.97038E-07 | -0.000491678 | 0.00124705 | 0.69 | 25.32644898 |
| family Clostridiales vadin BB60 group id.11286 | ebi-a-GCST90016932 | hypertension | ukb-b-12493 | rs6588624 | A | G | A | G | 0.0662317 | 0.0138147 | 1.79287E-06 | -0.000772455 | 0.000670082 | 0.25 | 22.98201846 |
| family Clostridiales vadin BB60 group id.11286 | ebi-a-GCST90016932 | hypertension | ukb-b-12493 | rs66714985 | A | C | A | C | 0.116908 | 0.0252447 | 4.85333E-06 | -0.000456042 | 0.00129573 | 0.719999 | 21.44308734 |
| family Clostridiales vadin BB60 group id.11286 | ebi-a-GCST90016932 | hypertension | ukb-b-12493 | rs7226487 | A | G | A | G | -0.0643682 | 0.0138701 | 3.58286E-06 | 0.00152393 | 0.000671654 | 0.0230001 | 21.53390662 |
| family Clostridiales vadin BB60 group id.11286 | ebi-a-GCST90016932 | hypertension | ukb-b-12493 | rs7538034 | T | G | T | G | -0.078598 | 0.0165982 | 2.36706E-06 | 0.000943361 | 0.000806273 | 0.24 | 22.4202414 |
| family Clostridiales vadin BB60 group id.11286 | ebi-a-GCST90016932 | hypertension | ukb-b-12493 | rs7725895 | A | G | A | G | -0.116224 | 0.0240367 | 3.94357E-06 | 0.00220676 | 0.00134853 | 0.1 | 23.37659382 |
| family Clostridiales vadin BB60 group id.11286 | ebi-a-GCST90016932 | hypertension | ukb-b-12493 | rs989682 | A | G | A | G | 0.070194 | 0.0155364 | 6.84715E-06 | 0.000315619 | 0.000728047 | 0.66 | 20.40978714 |
| genus Clostridium innocuum group id.14397 | ebi-a-GCST90016979 | hypertension | ukb-b-12493 | rs10074000 | T | C | T | C | -0.102648 | 0.0227508 | 6.99939E-06 | -0.00004245 | 0.000683568 | 0.95 | 20.3538664 |
| genus Clostridium innocuum group id.14397 | ebi-a-GCST90016979 | hypertension | ukb-b-12493 | rs10506058 | A | G | A | G | 0.0997048 | 0.0221926 | 8.92442E-06 | 0.000729018 | 0.000678704 | 0.28 | 20.18157439 |
| genus Clostridium innocuum group id.14397 | ebi-a-GCST90016979 | hypertension | ukb-b-12493 | rs1942371 | G | A | G | A | -0.157938 | 0.034187 | 4.0634E-06 | -0.000673102 | 0.00105305 | 0.52 | 21.33981082 |
| genus Clostridium innocuum group id.14397 | ebi-a-GCST90016979 | hypertension | ukb-b-12493 | rs40656 | C | T | C | T | 0.142664 | 0.0311021 | 8.61529E-06 | 0.000142305 | 0.00090616 | 0.88 | 21.03723446 |
| genus Clostridium innocuum group id.14397 | ebi-a-GCST90016979 | hypertension | ukb-b-12493 | rs4869133 | G | A | G | A | -0.180591 | 0.0409505 | 7.24453E-06 | -0.000559178 | 0.00121955 | 0.649999 | 19.4452293 |
| genus Clostridium innocuum group id.14397 | ebi-a-GCST90016979 | hypertension | ukb-b-12493 | rs61267978 | T | C | T | C | 0.14708 | 0.0320875 | 5.58509E-06 | 0.000583671 | 0.000982613 | 0.55 | 21.00751875 |
| genus Clostridium innocuum group id.14397 | ebi-a-GCST90016979 | hypertension | ukb-b-12493 | rs6577484 | G | A | G | A | 0.160425 | 0.0360857 | 8.40601E-06 | 0.000846295 | 0.00102794 | 0.41 | 19.76119065 |
| genus Clostridium innocuum group id.14397 | ebi-a-GCST90016979 | hypertension | ukb-b-12493 | rs6890185 | C | T | C | T | -0.113424 | 0.0233137 | 1.12243E-06 | -0.00156705 | 0.000709024 | 0.0269998 | 23.66610524 |
| genus Clostridium innocuum group id.14397 | ebi-a-GCST90016979 | hypertension | ukb-b-12493 | rs77845139 | A | G | A | G | -0.114993 | 0.0257186 | 8.40621E-06 | -0.000219375 | 0.000768058 | 0.780001 | 19.98883308 |
| genus Desulfovibrio id.3173 | ebi-a-GCST90016987 | hypertension | ukb-b-12493 | rs12031543 | T | C | T | C | -0.127185 | 0.0281887 | 6.54757E-06 | -0.000639975 | 0.00143483 | 0.66 | 20.3545251 |
| genus Desulfovibrio id.3173 | ebi-a-GCST90016987 | hypertension | ukb-b-12493 | rs13066142 | G | A | G | A | 0.119141 | 0.0250891 | 3.78669E-06 | 0.000243449 | 0.00121224 | 0.84 | 22.54714707 |
| genus Desulfovibrio id.3173 | ebi-a-GCST90016987 | hypertension | ukb-b-12493 | rs16863365 | A | G | A | G | 0.109402 | 0.0226964 | 1.79449E-06 | -0.000633164 | 0.000993848 | 0.52 | 23.23142501 |
| genus Desulfovibrio id.3173 | ebi-a-GCST90016987 | hypertension | ukb-b-12493 | rs2032031 | G | A | G | A | 0.0654931 | 0.0148681 | 9.14282E-06 | -1.55288E-05 | 0.000670406 | 0.98 | 19.40079063 |
| genus Desulfovibrio id.3173 | ebi-a-GCST90016987 | hypertension | ukb-b-12493 | rs2456226 | A | C | A | C | 0.0854927 | 0.0172749 | 8.5487E-07 | -0.00179521 | 0.000952936 | 0.0599998 | 24.48872999 |
| genus Desulfovibrio id.3173 | ebi-a-GCST90016987 | hypertension | ukb-b-12493 | rs2590913 | A | G | A | G | -0.154496 | 0.0339396 | 6.65426E-06 | 0.00268166 | 0.00123303 | 0.0299999 | 20.71859596 |
| genus Desulfovibrio id.3173 | ebi-a-GCST90016987 | hypertension | ukb-b-12493 | rs2853179 | T | C | T | C | -0.0811971 | 0.0174234 | 2.42438E-06 | 0.00123147 | 0.000793029 | 0.12 | 21.71473348 |
| genus Desulfovibrio id.3173 | ebi-a-GCST90016987 | hypertension | ukb-b-12493 | rs4797774 | A | G | A | G | -0.212582 | 0.0470044 | 5.64313E-06 | 0.00319777 | 0.00160535 | 0.0460002 | 20.45103214 |
| genus Desulfovibrio id.3173 | ebi-a-GCST90016987 | hypertension | ukb-b-12493 | rs6580353 | T | C | T | C | 0.0770979 | 0.0169735 | 4.93856E-06 | 2.42496E-05 | 0.000814727 | 0.98 | 20.62916118 |
| genus Desulfovibrio id.3173 | ebi-a-GCST90016987 | hypertension | ukb-b-12493 | rs72647089 | T | G | T | G | -0.106594 | 0.0239337 | 8.29755E-06 | -0.00121134 | 0.001123 | 0.28 | 19.8328494 |
| genus Flavonifractor id.2059 | ebi-a-GCST90017010 | hypertension | ukb-b-12493 | rs114873521 | C | T | C | T | -0.130067 | 0.0294087 | 7.12525E-06 | 0.00246011 | 0.00136479 | 0.0710003 | 19.55788433 |
| genus Flavonifractor id.2059 | ebi-a-GCST90017010 | hypertension | ukb-b-12493 | rs11811696 | T | C | T | C | -0.116063 | 0.0241012 | 2.07348E-06 | 0.000600403 | 0.0011524 | 0.6 | 23.18726532 |
| genus Flavonifractor id.2059 | ebi-a-GCST90017010 | hypertension | ukb-b-12493 | rs12030302 | G | A | G | A | 0.0692276 | 0.0137467 | 5.60841E-07 | -0.000343476 | 0.000669745 | 0.61 | 25.35717816 |
| genus Flavonifractor id.2059 | ebi-a-GCST90017010 | hypertension | ukb-b-12493 | rs34066017 | A | G | A | G | 0.0764294 | 0.0159789 | 1.52086E-06 | -0.000392257 | 0.000760562 | 0.61 | 22.87528026 |
| genus Flavonifractor id.2059 | ebi-a-GCST90017010 | hypertension | ukb-b-12493 | rs806808 | C | T | C | T | -0.0667272 | 0.0136557 | 1.17972E-06 | 0.000842432 | 0.000666315 | 0.21 | 23.873558 |
| genus Olsenella id.822 | ebi-a-GCST90017035 | hypertension | ukb-b-12493 | rs1035588 | A | G | A | G | -0.108148 | 0.0236848 | 4.85501E-06 | 2.85274E-06 | 0.000693825 | 1 | 20.84667625 |
| genus Olsenella id.822 | ebi-a-GCST90017035 | hypertension | ukb-b-12493 | rs17148768 | G | A | G | A | 0.140434 | 0.0295602 | 2.19842E-06 | 0.00110602 | 0.000853853 | 0.2 | 22.56675307 |
| genus Olsenella id.822 | ebi-a-GCST90017035 | hypertension | ukb-b-12493 | rs2759329 | A | G | A | G | 0.111131 | 0.0237218 | 3.43202E-06 | 0.000841002 | 0.000687203 | 0.22 | 21.94393182 |
| genus Olsenella id.822 | ebi-a-GCST90017035 | hypertension | ukb-b-12493 | rs35225860 | A | G | A | G | -0.223604 | 0.0482391 | 3.87044E-06 | 0.000705481 | 0.00136726 | 0.61 | 21.48325207 |
| genus Olsenella id.822 | ebi-a-GCST90017035 | hypertension | ukb-b-12493 | rs6046522 | C | T | C | T | 0.122938 | 0.0269928 | 4.48456E-06 | 0.000876136 | 0.000764631 | 0.25 | 20.74033153 |
| genus Olsenella id.822 | ebi-a-GCST90017035 | hypertension | ukb-b-12493 | rs61090148 | A | G | A | G | -0.104783 | 0.0231343 | 6.43724E-06 | -0.00161227 | 0.000673707 | 0.017 | 20.51200961 |
| genus Olsenella id.822 | ebi-a-GCST90017035 | hypertension | ukb-b-12493 | rs62112538 | C | T | C | T | -0.19943 | 0.0407033 | 1.1882E-06 | -0.000761651 | 0.00121802 | 0.53 | 24.00275087 |
| genus Olsenella id.822 | ebi-a-GCST90017035 | hypertension | ukb-b-12493 | rs72691585 | C | A | C | A | -0.249079 | 0.0520814 | 2.95291E-06 | -0.00139634 | 0.00147978 | 0.35 | 22.86905441 |
| genus Olsenella id.822 | ebi-a-GCST90017035 | hypertension | ukb-b-12493 | rs7540303 | C | T | C | T | 0.108044 | 0.023638 | 5.32023E-06 | 0.000973575 | 0.000679849 | 0.15 | 20.88907132 |
| genus Olsenella id.822 | ebi-a-GCST90017035 | hypertension | ukb-b-12493 | rs8066522 | A | G | A | G | 0.106529 | 0.0240379 | 9.70085E-06 | -0.00138576 | 0.000704058 | 0.0490004 | 19.63730715 |
| genus Olsenella id.822 | ebi-a-GCST90017035 | hypertension | ukb-b-12493 | rs9460691 | C | A | C | A | 0.119966 | 0.0268645 | 7.2835E-06 | 0.00090336 | 0.000802597 | 0.26 | 19.93875853 |
| genus Parabacteroides id.954 | ebi-a-GCST90017039 | hypertension | ukb-b-12493 | rs114567323 | T | C | T | C | 0.186478 | 0.0405199 | 5.65484E-06 | -0.0045568 | 0.00157258 | 0.00379997 | 21.17667426 |
| genus Parabacteroides id.954 | ebi-a-GCST90017039 | hypertension | ukb-b-12493 | rs115602804 | G | A | G | A | 0.10308 | 0.0222736 | 1.93059E-06 | -0.000852154 | 0.00144913 | 0.56 | 21.41446694 |
| genus Parabacteroides id.954 | ebi-a-GCST90017039 | hypertension | ukb-b-12493 | rs4236095 | A | G | A | G | -0.0761961 | 0.0157042 | 1.93143E-06 | -0.000106071 | 0.000968734 | 0.91 | 23.53819367 |
| genus Parabacteroides id.954 | ebi-a-GCST90017039 | hypertension | ukb-b-12493 | rs60884758 | C | T | C | T | -0.0702667 | 0.0142249 | 5.7069E-07 | -0.000242716 | 0.000883763 | 0.780001 | 24.39719809 |
| genus Parabacteroides id.954 | ebi-a-GCST90017039 | hypertension | ukb-b-12493 | rs6657302 | T | C | T | C | -0.10452 | 0.0225521 | 9.75708E-06 | 0.00117901 | 0.0014233 | 0.41 | 21.47652964 |
| genus Parabacteroides id.954 | ebi-a-GCST90017039 | hypertension | ukb-b-12493 | rs7298818 | C | T | C | T | 0.0888832 | 0.0200902 | 8.53631E-06 | -0.00201361 | 0.00119354 | 0.0920005 | 19.57086963 |
| genus Senegalimassilia id.11160 | ebi-a-GCST90017068 | hypertension | ukb-b-12493 | rs10036909 | C | T | C | T | 0.185519 | 0.0400881 | 8.05434E-06 | -0.00152921 | 0.00131994 | 0.25 | 21.41337506 |
| genus Senegalimassilia id.11160 | ebi-a-GCST90017068 | hypertension | ukb-b-12493 | rs11787826 | C | A | C | A | 0.081327 | 0.0171154 | 2.63463E-06 | -0.000626243 | 0.000667742 | 0.35 | 22.57535969 |
| genus Senegalimassilia id.11160 | ebi-a-GCST90017068 | hypertension | ukb-b-12493 | rs1990708 | A | C | A | C | -0.109619 | 0.0247785 | 8.90574E-06 | 0.000787185 | 0.000969265 | 0.42 | 19.56865281 |
| genus Senegalimassilia id.11160 | ebi-a-GCST90017068 | hypertension | ukb-b-12493 | rs2017373 | C | T | C | T | 0.0782258 | 0.0176841 | 9.50298E-06 | -0.00138496 | 0.00068992 | 0.0449997 | 19.56470857 |
| genus Senegalimassilia id.11160 | ebi-a-GCST90017068 | hypertension | ukb-b-12493 | rs7225245 | A | G | A | G | -0.079173 | 0.0170421 | 4.18013E-06 | 0.000803819 | 0.000697624 | 0.25 | 21.57979252 |
| Interleukin-2 receptor subunit alpha levels | ebi-a-GCST90010228 | hypertension | ukb-b-12493 | rs112352944 | A | G | A | G | -0.511796 | 0.108514 | 3.56016E-06 | 0.00295901 | 0.00147713 | 0.0449997 | 22.21085121 |
| Interleukin-2 receptor subunit alpha levels | ebi-a-GCST90010228 | hypertension | ukb-b-12493 | rs114977391 | T | C | T | C | -0.625262 | 0.124889 | 7.82943E-07 | 0.000396577 | 0.0021676 | 0.85 | 25.02756903 |
| Interleukin-2 receptor subunit alpha levels | ebi-a-GCST90010228 | hypertension | ukb-b-12493 | rs12617664 | G | T | G | T | 0.304482 | 0.0607014 | 7.83917E-07 | -0.00159184 | 0.00109759 | 0.15 | 25.12284422 |
| Interleukin-2 receptor subunit alpha levels | ebi-a-GCST90010228 | hypertension | ukb-b-12493 | rs12722497 | A | C | A | C | 0.86916 | 0.0558773 | 7.46621E-43 | -0.00245618 | 0.00118058 | 0.0369999 | 241.585915 |
| Interleukin-2 receptor subunit alpha levels | ebi-a-GCST90010228 | hypertension | ukb-b-12493 | rs186611585 | A | C | A | C | -0.541309 | 0.118836 | 8.27466E-06 | 0.00353689 | 0.00194748 | 0.0690001 | 20.71750341 |
| Interleukin-2 receptor subunit alpha levels | ebi-a-GCST90010228 | hypertension | ukb-b-12493 | rs41367851 | T | C | T | C | 0.508328 | 0.0957316 | 1.73996E-07 | -0.00113866 | 0.00132287 | 0.39 | 28.15272274 |
| Interleukin-2 receptor subunit alpha levels | ebi-a-GCST90010228 | hypertension | ukb-b-12493 | rs62066672 | A | G | A | G | -0.299369 | 0.0600122 | 8.58183E-07 | 0.000242335 | 0.000858504 | 0.780001 | 24.8472043 |
| Interleukin-2 receptor subunit alpha levels | ebi-a-GCST90010228 | hypertension | ukb-b-12493 | rs6510763 | C | T | C | T | 0.233449 | 0.0460545 | 6.3947E-07 | -0.000346798 | 0.000728076 | 0.630001 | 25.65564077 |
| Interleukin-2 receptor subunit alpha levels | ebi-a-GCST90010228 | hypertension | ukb-b-12493 | rs71427735 | T | C | T | C | 0.220765 | 0.0462698 | 2.48949E-06 | 0.000691318 | 0.000848228 | 0.42 | 22.73045725 |
| Interleukin-2 receptor subunit alpha levels | ebi-a-GCST90010228 | hypertension | ukb-b-12493 | rs75881090 | T | G | T | G | -0.449537 | 0.0951948 | 3.11652E-06 | 0.000784558 | 0.00154263 | 0.61 | 22.26626676 |
| Interleukin-2 receptor subunit alpha levels | ebi-a-GCST90010228 | hypertension | ukb-b-12493 | rs77050301 | T | C | T | C | 0.928976 | 0.2061 | 8.72308E-06 | -0.000957691 | 0.00332406 | 0.77 | 20.28597931 |
| Interleukin-2 receptor subunit alpha levels | ebi-a-GCST90010228 | hypertension | ukb-b-12493 | rs9590858 | A | G | A | G | 0.458435 | 0.100883 | 6.97927E-06 | -0.00214346 | 0.00171944 | 0.21 | 20.61875939 |
| Interleukin-11 receptor subunit alpha | prot-a-1467 | hypertension | ukb-b-12493 | rs10164427 | A | C | A | C | 0.2585 | 0.0483 | 8.51138E-08 | 0.00060715 | 0.0013278 | 0.649999 | 28.62619495 |
| Interleukin-11 receptor subunit alpha | prot-a-1467 | hypertension | ukb-b-12493 | rs11575578 | A | G | A | G | 0.5065 | 0.0479 | 4.36516E-26 | -0.00433899 | 0.00134165 | 0.0012 | 111.7441159 |
| Interleukin-11 receptor subunit alpha | prot-a-1467 | hypertension | ukb-b-12493 | rs116926439 | A | G | A | G | 0.1666 | 0.0307 | 5.7544E-08 | 0.000687123 | 0.000822485 | 0.4 | 29.4313399 |
| Interleukin-11 receptor subunit alpha | prot-a-1467 | hypertension | ukb-b-12493 | rs117616482 | T | C | T | C | -0.4044 | 0.0911 | 9.12011E-06 | -0.00121604 | 0.00253219 | 0.630001 | 19.69347387 |
| Interleukin-11 receptor subunit alpha | prot-a-1467 | hypertension | ukb-b-12493 | rs12326173 | G | A | G | A | 0.1165 | 0.0253 | 4.36516E-06 | -0.000150818 | 0.000674829 | 0.82 | 21.19081205 |
| Interleukin-11 receptor subunit alpha | prot-a-1467 | hypertension | ukb-b-12493 | rs13411098 | T | C | T | C | -0.1611 | 0.0338 | 1.86209E-06 | 0.0013418 | 0.000877279 | 0.13 | 22.70358666 |
| Interleukin-11 receptor subunit alpha | prot-a-1467 | hypertension | ukb-b-12493 | rs142942912 | A | C | A | C | 0.3739 | 0.0785 | 1.94984E-06 | -0.00452372 | 0.00202985 | 0.0259998 | 22.67296972 |
| Interleukin-11 receptor subunit alpha | prot-a-1467 | hypertension | ukb-b-12493 | rs143851711 | T | G | T | G | 0.4214 | 0.0925 | 5.24807E-06 | -0.0019791 | 0.00238627 | 0.41 | 20.74160638 |
| Interleukin-11 receptor subunit alpha | prot-a-1467 | hypertension | ukb-b-12493 | rs2299366 | G | T | G | T | 0.1184 | 0.0259 | 4.89779E-06 | 0.000608136 | 0.000700397 | 0.39 | 20.88529759 |
| Interleukin-11 receptor subunit alpha | prot-a-1467 | hypertension | ukb-b-12493 | rs41281585 | G | A | G | A | -0.4268 | 0.0906 | 2.45471E-06 | -9.78397E-05 | 0.0022849 | 0.97 | 22.17834921 |
| Interleukin-11 receptor subunit alpha | prot-a-1467 | hypertension | ukb-b-12493 | rs570551 | A | G | A | G | -0.161 | 0.0363 | 9.33254E-06 | -0.000280531 | 0.000967981 | 0.77 | 19.65962788 |
| Interleukin-11 receptor subunit alpha | prot-a-1467 | hypertension | ukb-b-12493 | rs61951410 | T | C | T | C | -0.1087 | 0.0244 | 8.12831E-06 | 8.81631E-05 | 0.000669793 | 0.9 | 19.83427027 |
| Interleukin-11 receptor subunit alpha | prot-a-1467 | hypertension | ukb-b-12493 | rs6741501 | C | A | C | A | 0.1596 | 0.0337 | 2.13796E-06 | -0.00074354 | 0.000907974 | 0.41 | 22.4152075 |
| Interleukin-11 receptor subunit alpha | prot-a-1467 | hypertension | ukb-b-12493 | rs7107075 | G | C | G | C | -0.2643 | 0.0553 | 1.7378E-06 | 0.00038825 | 0.00141519 | 0.780001 | 22.82868286 |
| Interleukin-11 receptor subunit alpha | prot-a-1467 | hypertension | ukb-b-12493 | rs77401577 | A | G | A | G | 0.2646 | 0.0595 | 8.51138E-06 | -0.00107512 | 0.00160415 | 0.5 | 19.76435015 |
| Interleukin-11 receptor subunit alpha | prot-a-1467 | hypertension | ukb-b-12493 | rs790952 | T | A | T | A | 0.1204 | 0.0267 | 6.76083E-06 | 0.000207162 | 0.000695008 | 0.77 | 20.32203721 |
| Interleukin-23 | prot-a-1471 | hypertension | ukb-b-12493 | rs10225112 | T | C | T | C | 0.2594 | 0.0584 | 9.12011E-06 | 0.000184623 | 0.00162657 | 0.91 | 19.71746327 |
| Interleukin-23 | prot-a-1471 | hypertension | ukb-b-12493 | rs10811539 | A | G | A | G | -0.1682 | 0.0379 | 8.91251E-06 | -0.000149984 | 0.00101121 | 0.88 | 19.68386393 |
| Interleukin-23 | prot-a-1471 | hypertension | ukb-b-12493 | rs11039685 | A | G | A | G | 0.1685 | 0.0327 | 2.5704E-07 | 0.0013948 | 0.000879581 | 0.11 | 26.53634448 |
| Interleukin-23 | prot-a-1471 | hypertension | ukb-b-12493 | rs111547974 | A | G | A | G | -0.4134 | 0.088 | 2.69153E-06 | -0.000576625 | 0.00254246 | 0.82 | 22.05527065 |
| Interleukin-23 | prot-a-1471 | hypertension | ukb-b-12493 | rs111957807 | T | C | T | C | -0.5499 | 0.1173 | 2.75423E-06 | 0.000635266 | 0.00313666 | 0.84 | 21.96383671 |
| Interleukin-23 | prot-a-1471 | hypertension | ukb-b-12493 | rs117217556 | A | G | A | G | 0.4679 | 0.1002 | 3.01995E-06 | -0.000666092 | 0.00268079 | 0.8 | 21.79251926 |
| Interleukin-23 | prot-a-1471 | hypertension | ukb-b-12493 | rs11777715 | T | G | T | G | -0.2294 | 0.0516 | 8.70964E-06 | 0.00129751 | 0.00141789 | 0.36 | 19.75259754 |
| Interleukin-23 | prot-a-1471 | hypertension | ukb-b-12493 | rs12891718 | A | C | A | C | -0.1325 | 0.026 | 3.54813E-07 | 0.00116843 | 0.000703635 | 0.0969996 | 25.95504892 |
| Interleukin-23 | prot-a-1471 | hypertension | ukb-b-12493 | rs139654893 | G | A | G | A | -0.2244 | 0.0507 | 9.54993E-06 | -0.00347653 | 0.00137562 | 0.0109999 | 19.57792127 |
| Interleukin-23 | prot-a-1471 | hypertension | ukb-b-12493 | rs140397820 | G | A | G | A | -0.3559 | 0.0771 | 3.89045E-06 | -0.000720478 | 0.00223886 | 0.75 | 21.29531218 |
| Interleukin-23 | prot-a-1471 | hypertension | ukb-b-12493 | rs143809049 | A | G | A | G | -0.4336 | 0.0894 | 1.23027E-06 | 3.78748E-05 | 0.00259956 | 0.99 | 23.50933262 |
| Interleukin-23 | prot-a-1471 | hypertension | ukb-b-12493 | rs144952996 | G | A | G | A | 0.5192 | 0.1144 | 5.62341E-06 | 0.00494677 | 0.00327568 | 0.13 | 20.5851535 |
| Interleukin-23 | prot-a-1471 | hypertension | ukb-b-12493 | rs145967371 | A | T | A | T | -0.3329 | 0.0728 | 4.7863E-06 | 0.00011249 | 0.00191099 | 0.95 | 20.89785074 |
| Interleukin-23 | prot-a-1471 | hypertension | ukb-b-12493 | rs146315683 | G | A | G | A | -0.4138 | 0.0934 | 9.33254E-06 | -0.00156209 | 0.00269335 | 0.56 | 19.61661242 |
| Interleukin-23 | prot-a-1471 | hypertension | ukb-b-12493 | rs149113489 | G | T | G | T | 0.4677 | 0.1048 | 8.12831E-06 | 0.0059419 | 0.00263253 | 0.0239999 | 19.90439427 |
| Interleukin-23 | prot-a-1471 | hypertension | ukb-b-12493 | rs4564348 | C | T | C | T | -0.1102 | 0.0246 | 7.58578E-06 | 1.21714E-05 | 0.000668775 | 0.99 | 20.05532786 |
| Interleukin-23 | prot-a-1471 | hypertension | ukb-b-12493 | rs4877748 | T | C | T | C | 0.1992 | 0.0444 | 7.24436E-06 | 0.000394635 | 0.00118984 | 0.74 | 20.11636556 |
| Interleukin-23 | prot-a-1471 | hypertension | ukb-b-12493 | rs4921223 | G | A | G | A | 0.3111 | 0.0261 | 8.31764E-33 | 0.000387157 | 0.000713646 | 0.59 | 141.989359 |
| Interleukin-23 | prot-a-1471 | hypertension | ukb-b-12493 | rs58807708 | C | T | C | T | -0.2215 | 0.05 | 9.54993E-06 | -0.00040306 | 0.00134362 | 0.760001 | 19.61300972 |
| Interleukin-23 | prot-a-1471 | hypertension | ukb-b-12493 | rs59662975 | A | G | A | G | -0.1679 | 0.0347 | 1.28825E-06 | -0.00244895 | 0.000923433 | 0.008 | 23.39802679 |
| Interleukin-23 | prot-a-1471 | hypertension | ukb-b-12493 | rs62245853 | T | C | T | C | -0.1568 | 0.0306 | 2.88403E-07 | 0.00139671 | 0.000852708 | 0.1 | 26.24134282 |
| Interleukin-23 | prot-a-1471 | hypertension | ukb-b-12493 | rs6970647 | T | G | T | G | -0.1173 | 0.0261 | 6.76083E-06 | 0.000183761 | 0.00069453 | 0.79 | 20.1860712 |
| Interleukin-23 | prot-a-1471 | hypertension | ukb-b-12493 | rs7206392 | A | G | A | G | 0.2481 | 0.0541 | 4.46684E-06 | 0.00161141 | 0.00154379 | 0.3 | 21.01821303 |
| Interleukin-23 | prot-a-1471 | hypertension | ukb-b-12493 | rs7325982 | A | G | A | G | -0.1178 | 0.0255 | 3.89045E-06 | -0.000132633 | 0.000693403 | 0.85 | 21.32784674 |
| Interleukin-23 | prot-a-1471 | hypertension | ukb-b-12493 | rs74350446 | T | A | T | A | 0.4045 | 0.0896 | 6.30957E-06 | 0.00241776 | 0.00223007 | 0.28 | 20.36844261 |
| Interleukin-23 | prot-a-1471 | hypertension | ukb-b-12493 | rs74419705 | T | C | T | C | -0.3641 | 0.0816 | 8.12831E-06 | 0.00110368 | 0.00216824 | 0.61 | 19.89746876 |
| Interleukin-23 | prot-a-1471 | hypertension | ukb-b-12493 | rs74748552 | A | G | A | G | 0.3264 | 0.0675 | 1.31826E-06 | 0.00111702 | 0.00168728 | 0.51 | 23.36843055 |
| Interleukin-23 | prot-a-1471 | hypertension | ukb-b-12493 | rs76639786 | T | C | T | C | -0.4416 | 0.0987 | 7.76247E-06 | -0.00370773 | 0.00276425 | 0.18 | 20.00601616 |
| Interleukin-23 | prot-a-1471 | hypertension | ukb-b-12493 | rs9696499 | G | C | G | C | 0.2217 | 0.0473 | 2.75423E-06 | 0.00218844 | 0.00130074 | 0.0920005 | 21.95562963 |
| Interleukin-23 | prot-a-1471 | hypertension | ukb-b-12493 | rs9815073 | A | C | A | C | 0.2146 | 0.0277 | 8.70964E-15 | -0.000268099 | 0.000729422 | 0.709999 | 59.98417476 |
| Interleukin-1 receptor type 2 | prot-a-1498 | hypertension | ukb-b-12493 | rs113360963 | T | C | T | C | -0.2285 | 0.0499 | 4.67735E-06 | -0.00137997 | 0.00138735 | 0.32 | 20.95598644 |
| Interleukin-1 receptor type 2 | prot-a-1498 | hypertension | ukb-b-12493 | rs11576745 | T | C | T | C | -0.1206 | 0.0256 | 2.45471E-06 | -0.0013055 | 0.000678244 | 0.0539995 | 22.17948594 |
| Interleukin-1 receptor type 2 | prot-a-1498 | hypertension | ukb-b-12493 | rs117087150 | G | C | G | C | -0.2837 | 0.0625 | 5.62341E-06 | -0.00166508 | 0.0017289 | 0.34 | 20.59185295 |
| Interleukin-1 receptor type 2 | prot-a-1498 | hypertension | ukb-b-12493 | rs117896618 | C | G | C | G | 0.5236 | 0.1151 | 5.37032E-06 | 0.000809872 | 0.00318594 | 0.8 | 20.68166122 |
| Interleukin-1 receptor type 2 | prot-a-1498 | hypertension | ukb-b-12493 | rs11896555 | T | C | T | C | 0.1347 | 0.0272 | 7.76247E-07 | 0.00025206 | 0.000746109 | 0.74 | 24.50948437 |
| Interleukin-1 receptor type 2 | prot-a-1498 | hypertension | ukb-b-12493 | rs12110896 | A | G | A | G | 0.3499 | 0.0747 | 2.81838E-06 | 0.00172661 | 0.00236352 | 0.47 | 21.92721488 |
| Interleukin-1 receptor type 2 | prot-a-1498 | hypertension | ukb-b-12493 | rs13403306 | G | A | G | A | -0.1127 | 0.0252 | 7.76247E-06 | 0.000204609 | 0.000675058 | 0.760001 | 19.9886536 |
| Interleukin-1 receptor type 2 | prot-a-1498 | hypertension | ukb-b-12493 | rs1791618 | T | C | T | C | 0.1299 | 0.0279 | 3.16228E-06 | 0.000493689 | 0.000757096 | 0.51 | 21.66440106 |
| Interleukin-1 receptor type 2 | prot-a-1498 | hypertension | ukb-b-12493 | rs1892688 | G | T | G | T | 0.2759 | 0.0608 | 5.7544E-06 | -0.00059954 | 0.00164653 | 0.719999 | 20.5794154 |
| Interleukin-1 receptor type 2 | prot-a-1498 | hypertension | ukb-b-12493 | rs3744753 | A | G | A | G | 0.2915 | 0.0644 | 6.16595E-06 | 0.00357532 | 0.00164669 | 0.0299999 | 20.47586109 |
| Interleukin-1 receptor type 2 | prot-a-1498 | hypertension | ukb-b-12493 | rs6029202 | G | A | G | A | 0.123 | 0.0274 | 7.24436E-06 | -0.00106625 | 0.000723791 | 0.14 | 20.13937035 |
| Interleukin-1 receptor type 2 | prot-a-1498 | hypertension | ukb-b-12493 | rs62143194 | G | C | G | C | -0.1956 | 0.0306 | 1.54882E-10 | -0.000930255 | 0.000828362 | 0.26 | 40.83491342 |
| Interleukin-1 receptor type 2 | prot-a-1498 | hypertension | ukb-b-12493 | rs7115082 | T | C | T | C | -0.1154 | 0.0253 | 5.37032E-06 | -0.00207878 | 0.000684021 | 0.00239999 | 20.79253142 |
| Interleukin-1 receptor type 2 | prot-a-1498 | hypertension | ukb-b-12493 | rs7569422 | T | A | T | A | -0.5342 | 0.1186 | 6.60693E-06 | -0.00324789 | 0.00273965 | 0.24 | 20.27566843 |
| Interleukin-1 receptor type 2 | prot-a-1498 | hypertension | ukb-b-12493 | rs7724661 | G | A | G | A | 0.133 | 0.0294 | 6.0256E-06 | -0.000360375 | 0.000792039 | 0.649999 | 20.45245342 |
| Interleukin-1 receptor type 2 | prot-a-1498 | hypertension | ukb-b-12493 | rs9550571 | A | G | A | G | 0.183 | 0.0401 | 4.89779E-06 | -0.000488067 | 0.00110055 | 0.66 | 20.81374481 |
| Interleukin-27 | prot-a-1516 | hypertension | ukb-b-12493 | rs10144764 | G | A | G | A | 0.1215 | 0.0269 | 6.16595E-06 | -2.85449E-05 | 0.000721292 | 0.97 | 20.38847707 |
| Interleukin-27 | prot-a-1516 | hypertension | ukb-b-12493 | rs11466678 | G | A | G | A | -0.2489 | 0.0539 | 3.80189E-06 | -0.000368884 | 0.00148464 | 0.8 | 21.31125639 |
| Interleukin-27 | prot-a-1516 | hypertension | ukb-b-12493 | rs114753826 | C | T | C | T | -0.6138 | 0.1321 | 3.38844E-06 | -0.00344534 | 0.00360815 | 0.34 | 21.57669508 |
| Interleukin-27 | prot-a-1516 | hypertension | ukb-b-12493 | rs115225987 | C | G | C | G | 0.6003 | 0.1276 | 2.5704E-06 | -0.00226743 | 0.00389495 | 0.56 | 22.11933821 |
| Interleukin-27 | prot-a-1516 | hypertension | ukb-b-12493 | rs115522500 | C | T | C | T | -0.5364 | 0.1049 | 3.16228E-07 | 0.00291284 | 0.00301906 | 0.33 | 26.13144064 |
| Interleukin-27 | prot-a-1516 | hypertension | ukb-b-12493 | rs143291480 | C | T | C | T | -0.5593 | 0.1176 | 1.99526E-06 | 0.00178437 | 0.00345487 | 0.61 | 22.60537867 |
| Interleukin-27 | prot-a-1516 | hypertension | ukb-b-12493 | rs146347521 | C | T | C | T | 0.5366 | 0.1193 | 6.76083E-06 | -0.00465106 | 0.00281223 | 0.0980009 | 20.21888679 |
| Interleukin-27 | prot-a-1516 | hypertension | ukb-b-12493 | rs146454050 | G | T | G | T | 0.2651 | 0.0555 | 1.77828E-06 | -0.0025714 | 0.00155523 | 0.0980009 | 22.80186029 |
| Interleukin-27 | prot-a-1516 | hypertension | ukb-b-12493 | rs147629182 | T | C | T | C | 0.3384 | 0.0736 | 4.36516E-06 | 0.00116566 | 0.00196873 | 0.55 | 21.12719648 |
| Interleukin-27 | prot-a-1516 | hypertension | ukb-b-12493 | rs181209 | T | G | T | G | -0.1809 | 0.0265 | 8.70964E-12 | 0.00237312 | 0.000707995 | 0.0008 | 46.57170921 |
| Interleukin-27 | prot-a-1516 | hypertension | ukb-b-12493 | rs192884195 | G | A | G | A | -0.5558 | 0.1203 | 3.80189E-06 | 0.00222736 | 0.00293934 | 0.450001 | 21.33254259 |
| Interleukin-27 | prot-a-1516 | hypertension | ukb-b-12493 | rs2203908 | G | A | G | A | -0.1117 | 0.0252 | 9.33254E-06 | -0.000118467 | 0.000673893 | 0.86 | 19.63550412 |
| Interleukin-27 | prot-a-1516 | hypertension | ukb-b-12493 | rs2289303 | C | A | C | A | 0.1902 | 0.0406 | 2.75423E-06 | 0.000175072 | 0.00107793 | 0.87 | 21.93338941 |
| Interleukin-27 | prot-a-1516 | hypertension | ukb-b-12493 | rs55902334 | T | C | T | C | -0.2291 | 0.0501 | 4.7863E-06 | 0.00070722 | 0.00135834 | 0.6 | 20.89832688 |
| Interleukin-27 | prot-a-1516 | hypertension | ukb-b-12493 | rs56895056 | A | G | A | G | -0.1447 | 0.031 | 3.16228E-06 | 0.00100307 | 0.000863487 | 0.25 | 21.77461404 |
| Interleukin-27 | prot-a-1516 | hypertension | ukb-b-12493 | rs5762268 | A | G | A | G | -0.1813 | 0.0335 | 5.88844E-08 | 0.000169817 | 0.000918874 | 0.85 | 29.2713522 |
| Interleukin-27 | prot-a-1516 | hypertension | ukb-b-12493 | rs62090186 | C | T | C | T | 0.1484 | 0.0303 | 9.33254E-07 | -7.08991E-05 | 0.000801539 | 0.93 | 23.97283168 |
| Interleukin-27 | prot-a-1516 | hypertension | ukb-b-12493 | rs72900609 | G | A | G | A | -0.2234 | 0.0481 | 3.38844E-06 | 0.00100521 | 0.00126115 | 0.43 | 21.5582238 |
| Interleukin-27 | prot-a-1516 | hypertension | ukb-b-12493 | rs73350510 | C | A | C | A | -0.4627 | 0.1022 | 5.88844E-06 | 0.000913214 | 0.0029177 | 0.75 | 20.48490709 |
| Interleukin-27 | prot-a-1516 | hypertension | ukb-b-12493 | rs73427441 | T | C | T | C | -0.1571 | 0.0336 | 3.01995E-06 | 9.21402E-05 | 0.000910442 | 0.92 | 21.84794564 |
| Interleukin-27 | prot-a-1516 | hypertension | ukb-b-12493 | rs73806724 | T | A | T | A | 0.3844 | 0.0824 | 3.0903E-06 | -0.000260356 | 0.00234212 | 0.91 | 21.74949241 |
| Interleukin-27 | prot-a-1516 | hypertension | ukb-b-12493 | rs75878007 | A | G | A | G | -0.2549 | 0.057 | 7.76247E-06 | -0.00104268 | 0.00155129 | 0.5 | 19.98603993 |
| Interleukin-27 | prot-a-1516 | hypertension | ukb-b-12493 | rs76386091 | G | A | G | A | -0.2915 | 0.0627 | 3.38844E-06 | -0.00263023 | 0.00172311 | 0.13 | 21.60124724 |
| Interleukin-27 | prot-a-1516 | hypertension | ukb-b-12493 | rs79131308 | T | C | T | C | 0.3133 | 0.0698 | 7.07946E-06 | -0.00124357 | 0.00199139 | 0.53 | 20.13477291 |
| Interleukin-27 | prot-a-1516 | hypertension | ukb-b-12493 | rs79299408 | T | C | T | C | 0.4531 | 0.0982 | 3.98107E-06 | 0.00135105 | 0.00234161 | 0.56 | 21.27658585 |
| Interleukin-27 | prot-a-1516 | hypertension | ukb-b-12493 | rs79423863 | C | A | C | A | -0.2585 | 0.0581 | 8.51138E-06 | 0.0024062 | 0.00159527 | 0.13 | 19.78361361 |
| Interleukin-27 | prot-a-1516 | hypertension | ukb-b-12493 | rs9902988 | C | T | C | T | 0.5445 | 0.1205 | 6.16595E-06 | 0.00286808 | 0.00358162 | 0.42 | 20.40602741 |
| Interleukin-31 | prot-a-1521 | hypertension | ukb-b-12493 | rs10070652 | T | C | T | C | -0.1112 | 0.0249 | 7.76247E-06 | 0.00140125 | 0.000670892 | 0.0369999 | 19.93185282 |
| Interleukin-31 | prot-a-1521 | hypertension | ukb-b-12493 | rs10983188 | A | G | A | G | -0.2549 | 0.0572 | 8.31764E-06 | -0.000012264 | 0.00148717 | 0.99 | 19.84652176 |
| Interleukin-31 | prot-a-1521 | hypertension | ukb-b-12493 | rs11296 | C | T | C | T | 0.2452 | 0.0531 | 3.89045E-06 | -0.00146411 | 0.00147506 | 0.32 | 21.31025668 |
| Interleukin-31 | prot-a-1521 | hypertension | ukb-b-12493 | rs116496030 | T | C | T | C | -0.4394 | 0.0985 | 8.12831E-06 | 0.001728 | 0.00284866 | 0.54 | 19.88769429 |
| Interleukin-31 | prot-a-1521 | hypertension | ukb-b-12493 | rs117922006 | A | G | A | G | 0.5581 | 0.1048 | 0.0000001 | -0.00163436 | 0.00277743 | 0.56 | 28.34250755 |
| Interleukin-31 | prot-a-1521 | hypertension | ukb-b-12493 | rs13246161 | A | C | A | C | -0.1796 | 0.0373 | 1.44544E-06 | 0.00118254 | 0.00100297 | 0.24 | 23.17030722 |
| Interleukin-31 | prot-a-1521 | hypertension | ukb-b-12493 | rs1357244 | C | G | C | G | 0.2024 | 0.0457 | 9.33254E-06 | 0.000500231 | 0.0012042 | 0.68 | 19.60312943 |
| Interleukin-31 | prot-a-1521 | hypertension | ukb-b-12493 | rs138710866 | T | C | T | C | 0.3871 | 0.0862 | 7.07946E-06 | -0.00155303 | 0.0023501 | 0.51 | 20.154341 |
| Interleukin-31 | prot-a-1521 | hypertension | ukb-b-12493 | rs141176609 | A | T | A | T | 0.5279 | 0.1176 | 7.07946E-06 | -0.00201109 | 0.00311748 | 0.52 | 20.1384236 |
| Interleukin-31 | prot-a-1521 | hypertension | ukb-b-12493 | rs144456857 | A | C | A | C | 0.4141 | 0.0877 | 2.34423E-06 | 0.00107813 | 0.00239063 | 0.649999 | 22.28168681 |
| Interleukin-31 | prot-a-1521 | hypertension | ukb-b-12493 | rs16882647 | G | A | G | A | -0.2284 | 0.0486 | 2.63027E-06 | 8.49169E-05 | 0.00129414 | 0.95 | 22.07275038 |
| Interleukin-31 | prot-a-1521 | hypertension | ukb-b-12493 | rs17073215 | C | T | C | T | 0.3878 | 0.0823 | 2.45471E-06 | -0.00130417 | 0.00234038 | 0.58 | 22.1897664 |
| Interleukin-31 | prot-a-1521 | hypertension | ukb-b-12493 | rs186524720 | T | C | T | C | 0.4571 | 0.1011 | 6.16595E-06 | -0.00414782 | 0.00281344 | 0.14 | 20.42946166 |
| Interleukin-31 | prot-a-1521 | hypertension | ukb-b-12493 | rs35000575 | A | C | A | C | 0.1668 | 0.036 | 3.54813E-06 | 0.000377406 | 0.000962811 | 0.7 | 21.45477094 |
| Interleukin-31 | prot-a-1521 | hypertension | ukb-b-12493 | rs3773727 | A | G | A | G | -0.1385 | 0.031 | 7.76247E-06 | 0.000212851 | 0.000848708 | 0.8 | 19.94862426 |
| Interleukin-31 | prot-a-1521 | hypertension | ukb-b-12493 | rs57957159 | A | G | A | G | 0.1554 | 0.0341 | 5.37032E-06 | 0.000435819 | 0.000929084 | 0.64 | 20.75535004 |
| Interleukin-31 | prot-a-1521 | hypertension | ukb-b-12493 | rs59898539 | C | G | C | G | 0.1676 | 0.0339 | 7.58578E-07 | 0.00135685 | 0.000915261 | 0.14 | 24.42786006 |
| Interleukin-31 | prot-a-1521 | hypertension | ukb-b-12493 | rs704 | A | G | A | G | 0.4487 | 0.0234 | 8.70964E-82 | -0.00121538 | 0.000666065 | 0.0680002 | 367.4660453 |
| Interleukin-31 | prot-a-1521 | hypertension | ukb-b-12493 | rs71605701 | T | G | T | G | 0.2077 | 0.0435 | 1.7378E-06 | -0.00273491 | 0.00115718 | 0.0179999 | 22.78406812 |
| Interleukin-31 | prot-a-1521 | hypertension | ukb-b-12493 | rs74384635 | A | T | A | T | -0.2834 | 0.0621 | 5.12861E-06 | -0.00136461 | 0.00177294 | 0.44 | 20.81389133 |
| Interleukin-31 | prot-a-1521 | hypertension | ukb-b-12493 | rs7859904 | A | G | A | G | -0.2296 | 0.0455 | 4.36516E-07 | 0.000506242 | 0.00126144 | 0.69 | 25.44824079 |
| Interleukin-31 | prot-a-1521 | hypertension | ukb-b-12493 | rs78986499 | G | C | G | C | 0.3014 | 0.0643 | 2.75423E-06 | -0.000209419 | 0.00168317 | 0.9 | 21.95843283 |
| Interleukin-31 | prot-a-1521 | hypertension | ukb-b-12493 | rs80154319 | C | T | C | T | 0.2128 | 0.048 | 9.33254E-06 | 0.00103219 | 0.00129251 | 0.42 | 19.64253627 |
| Interleukin 1 receptor like 1 | prot-b-38 | hypertension | ukb-b-12493 | rs115261236 | G | T | G | T | 0.9216 | 0.2044 | 6.75694E-06 | 0.00371377 | 0.00241597 | 0.12 | 20.31735434 |
| Interleukin 1 receptor like 1 | prot-b-38 | hypertension | ukb-b-12493 | rs117511079 | A | C | A | C | 2.4136 | 0.4459 | 6.61302E-08 | 0.0108543 | 0.00345177 | 0.0017 | 29.28192711 |
| Interleukin 1 receptor like 1 | prot-b-38 | hypertension | ukb-b-12493 | rs1420101 | T | C | T | C | -0.5805 | 0.0227 | 2.0324E-132 | -0.00105309 | 0.000684448 | 0.12 | 653.5769681 |
| Interleukin 1 receptor like 1 | prot-b-38 | hypertension | ukb-b-12493 | rs17548810 | G | A | G | A | 0.4302 | 0.0959 | 7.46603E-06 | 0.000333128 | 0.00181979 | 0.85 | 20.11164541 |
| Interleukin 1 receptor like 1 | prot-b-38 | hypertension | ukb-b-12493 | rs186646814 | T | C | T | C | 0.579 | 0.1271 | 5.41801E-06 | 0.00178038 | 0.00250951 | 0.48 | 20.74006112 |
| Interleukin 1 receptor like 1 | prot-b-38 | hypertension | ukb-b-12493 | rs2006811 | A | G | A | G | -0.1772 | 0.0396 | 8.03193E-06 | 0.000616194 | 0.000760747 | 0.42 | 20.01156569 |
| Interleukin 1 receptor like 1 | prot-b-38 | hypertension | ukb-b-12493 | rs2012408 | A | G | A | G | 0.2652 | 0.0542 | 1.04701E-06 | -0.00151813 | 0.00107429 | 0.16 | 23.92723263 |
| Interleukin 1 receptor like 1 | prot-b-38 | hypertension | ukb-b-12493 | rs2241132 | A | C | A | C | -0.235 | 0.0333 | 2.01604E-12 | 0.00142987 | 0.000983754 | 0.15 | 49.77270719 |
| Interleukin 1 receptor like 1 | prot-b-38 | hypertension | ukb-b-12493 | rs35166255 | A | G | A | G | 0.464 | 0.076 | 1.16399E-09 | -0.000417965 | 0.00185526 | 0.82 | 37.25227344 |
| Interleukin 1 receptor like 1 | prot-b-38 | hypertension | ukb-b-12493 | rs79516097 | G | A | G | A | 1.3212 | 0.2807 | 2.62198E-06 | 0.00639549 | 0.00337052 | 0.0580003 | 22.14095302 |
| genus Clostridium innocuum group id.14397 | ebi-a-GCST90016979 | Interleukin-1 receptor type 2 | prot-a-1498 | rs10074000 | T | C | T | C | -0.102648 | 0.0227508 | 6.99939E-06 | 0.0002 | 0.0258 | 1 | 20.3538664 |
| genus Clostridium innocuum group id.14397 | ebi-a-GCST90016979 | Interleukin-1 receptor type 2 | prot-a-1498 | rs10506058 | A | G | A | G | 0.0997048 | 0.0221926 | 8.92442E-06 | 0.026 | 0.0254 | 0.30903 | 20.18157439 |
| genus Clostridium innocuum group id.14397 | ebi-a-GCST90016979 | Interleukin-1 receptor type 2 | prot-a-1498 | rs1942371 | G | A | G | A | -0.157938 | 0.034187 | 4.0634E-06 | -0.0497 | 0.0389 | 0.199526 | 21.33981082 |
| genus Clostridium innocuum group id.14397 | ebi-a-GCST90016979 | Interleukin-1 receptor type 2 | prot-a-1498 | rs40656 | C | T | C | T | 0.142664 | 0.0311021 | 8.61529E-06 | 0.0897 | 0.0346 | 0.00954993 | 21.03723446 |
| genus Clostridium innocuum group id.14397 | ebi-a-GCST90016979 | Interleukin-1 receptor type 2 | prot-a-1498 | rs4869133 | G | A | G | A | -0.180591 | 0.0409505 | 7.24453E-06 | -0.0557 | 0.0456 | 0.223872 | 19.4452293 |
| genus Clostridium innocuum group id.14397 | ebi-a-GCST90016979 | Interleukin-1 receptor type 2 | prot-a-1498 | rs61267978 | T | C | T | C | 0.14708 | 0.0320875 | 5.58509E-06 | 0.0058 | 0.0366 | 0.870964 | 21.00751875 |
| genus Clostridium innocuum group id.14397 | ebi-a-GCST90016979 | Interleukin-1 receptor type 2 | prot-a-1498 | rs6577484 | G | A | G | A | 0.160425 | 0.0360857 | 8.40601E-06 | 0.028 | 0.0373 | 0.457088 | 19.76119065 |
| genus Clostridium innocuum group id.14397 | ebi-a-GCST90016979 | Interleukin-1 receptor type 2 | prot-a-1498 | rs6890185 | C | T | C | T | -0.113424 | 0.0233137 | 1.12243E-06 | -0.0092 | 0.0269 | 0.74131 | 23.66610524 |
| genus Clostridium innocuum group id.14397 | ebi-a-GCST90016979 | Interleukin-1 receptor type 2 | prot-a-1498 | rs77845139 | A | G | A | G | -0.114993 | 0.0257186 | 8.40621E-06 | 0.0313 | 0.0287 | 0.275423 | 19.98883308 |

**S3. Funnel plot of the causal effect of family Clostridiales vadin BB60 group id.11286 (A), genus Clostridium innocuum group id.14397 (B), genus Desulfovibrio id.3173 (C), genus Flavonifractor id.2059 (D), genus Olsenella id.822 (E), genus Parabacteroides id.954 (F) and genus Senegalimassilia id.11160 (G) on hypertension.**

**A.**
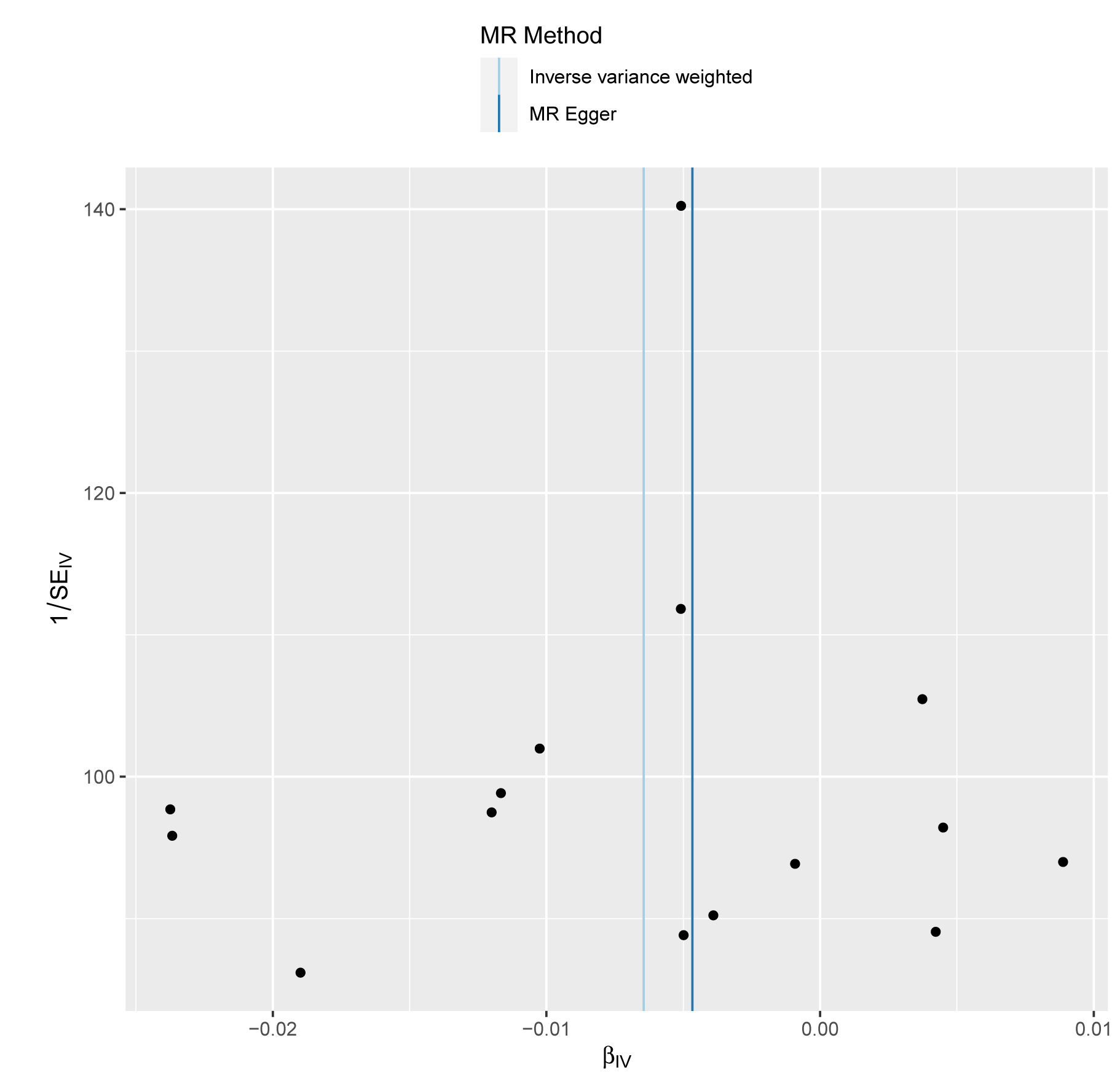
**B.**
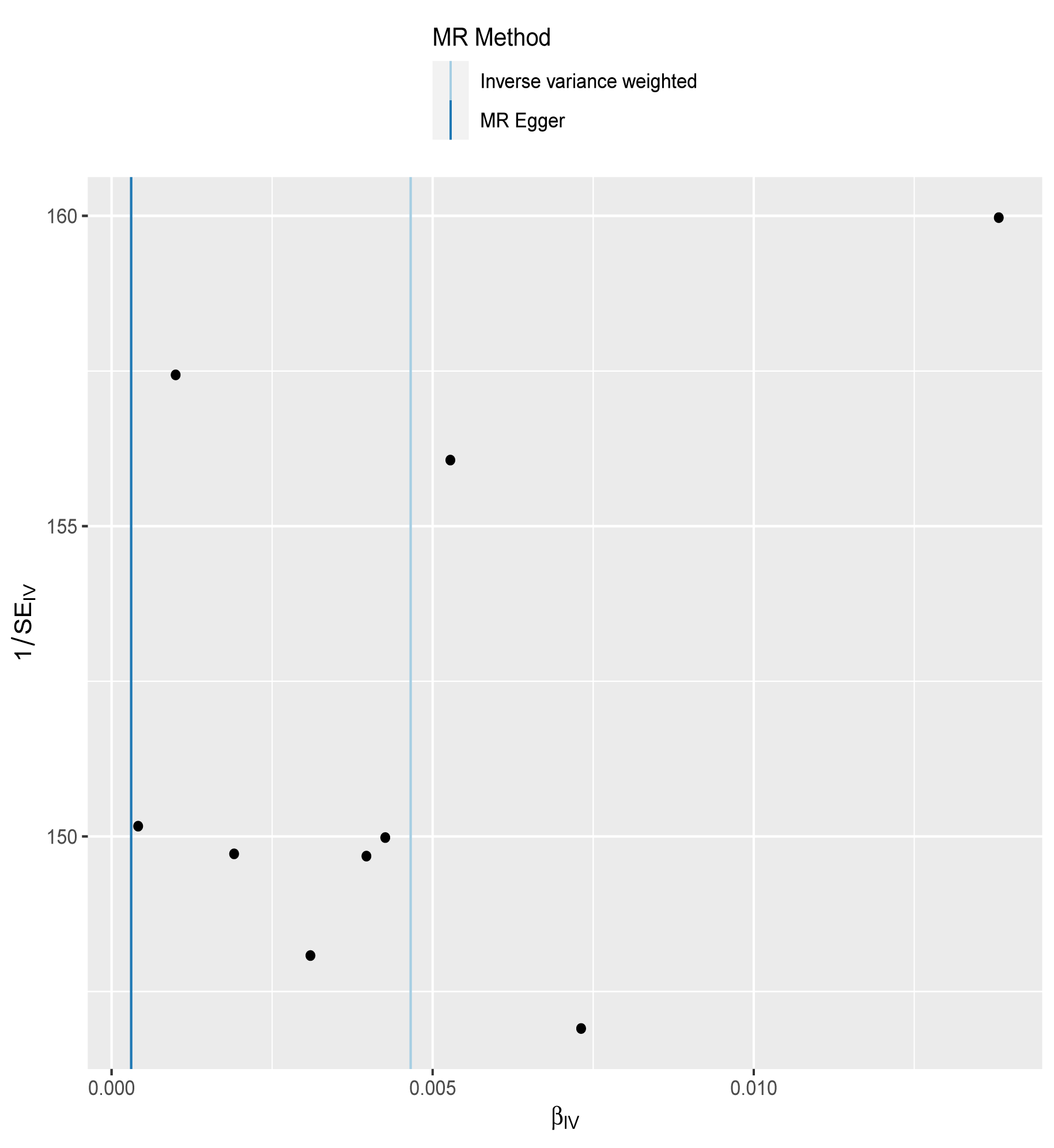
**C.**
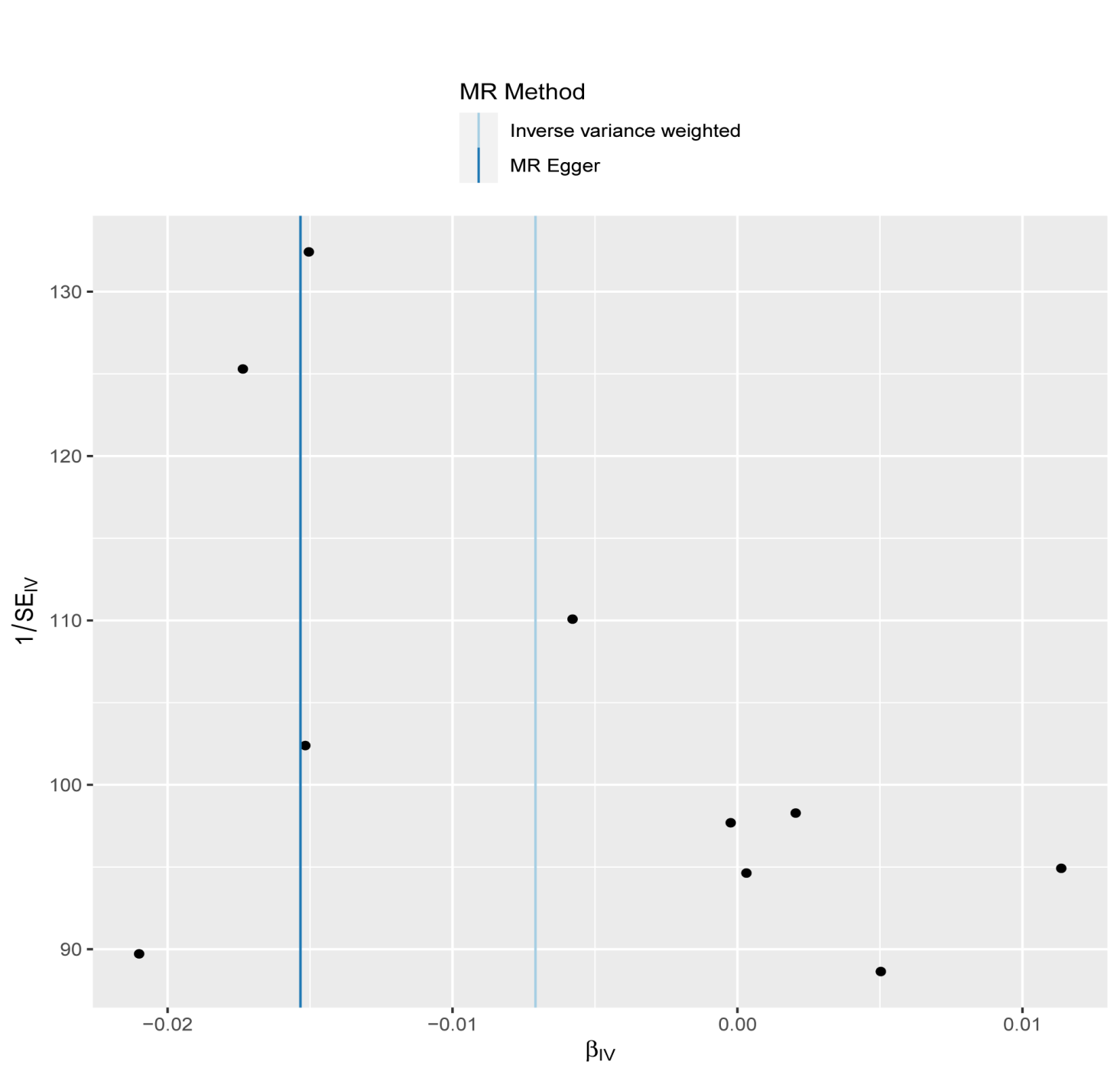


**D.**
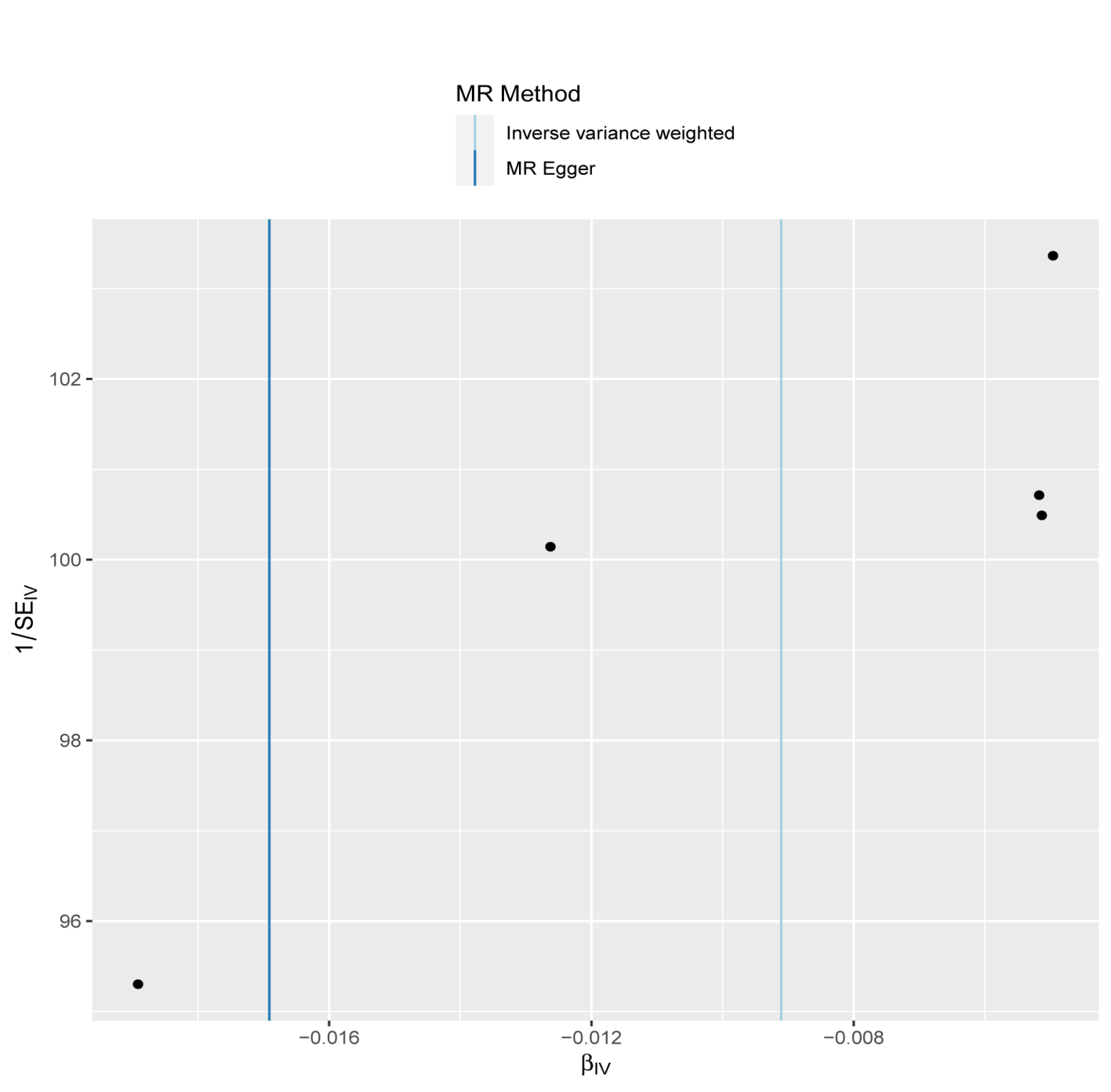
**E.**
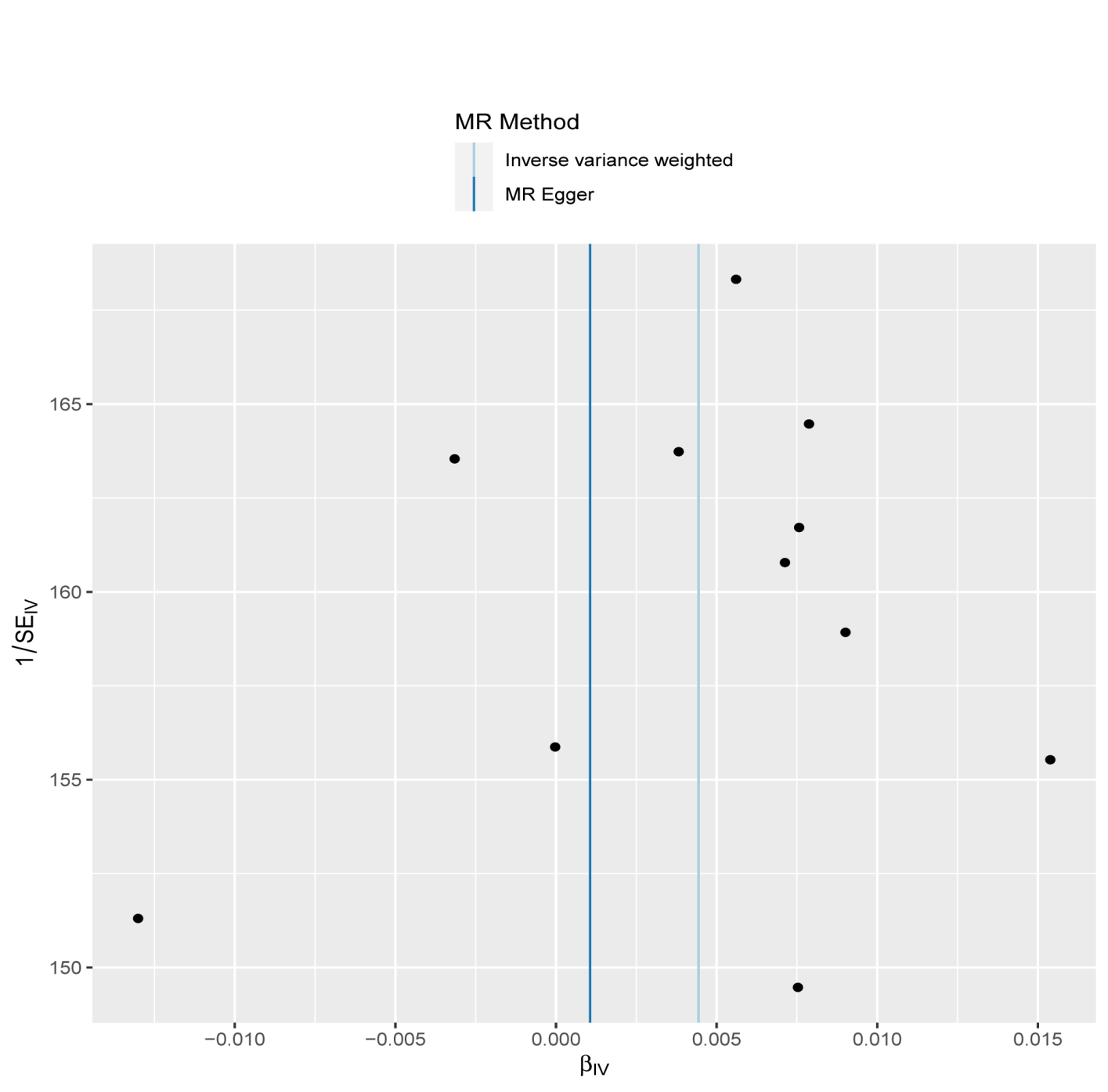
**F.**
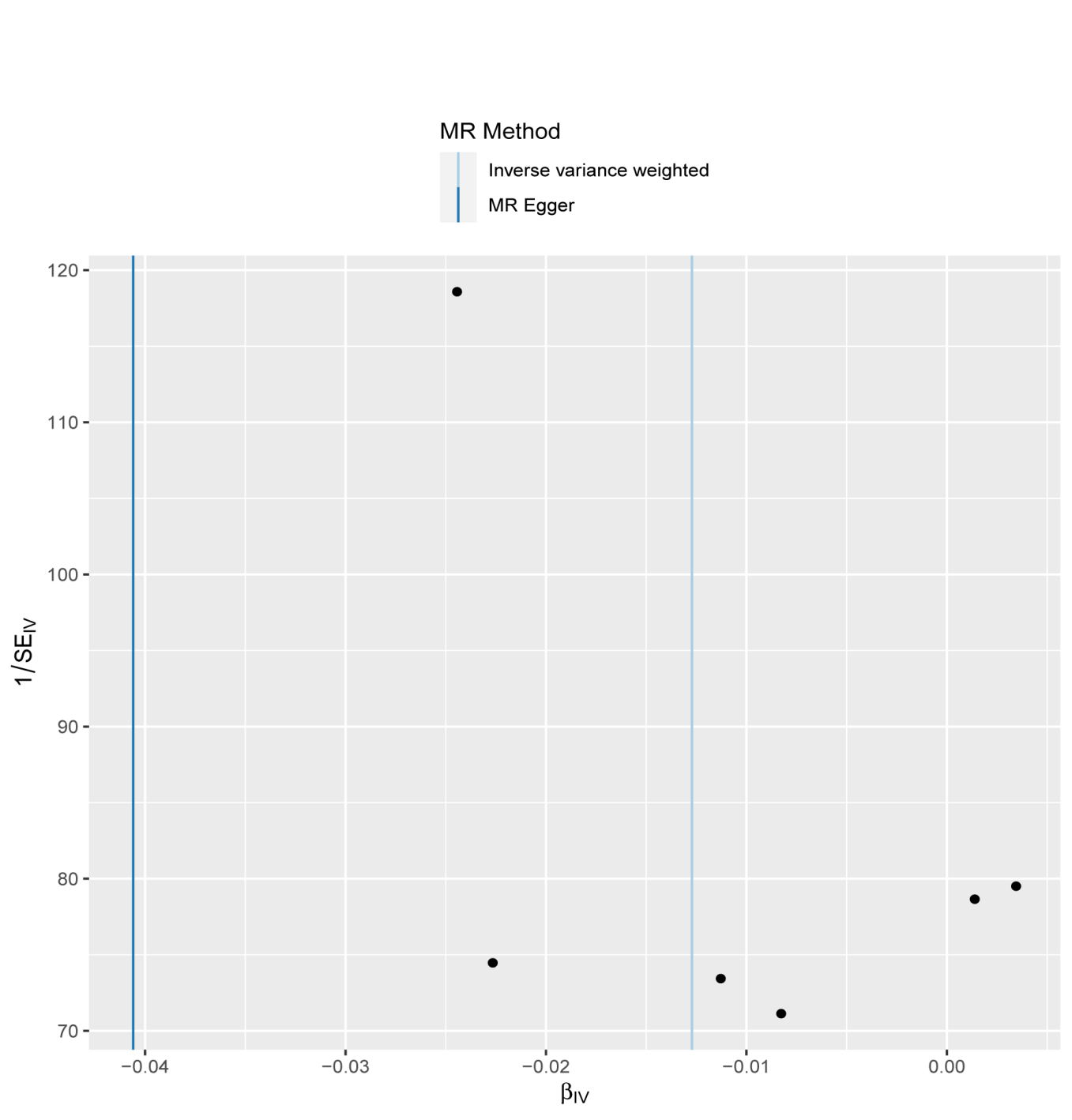


**G.**
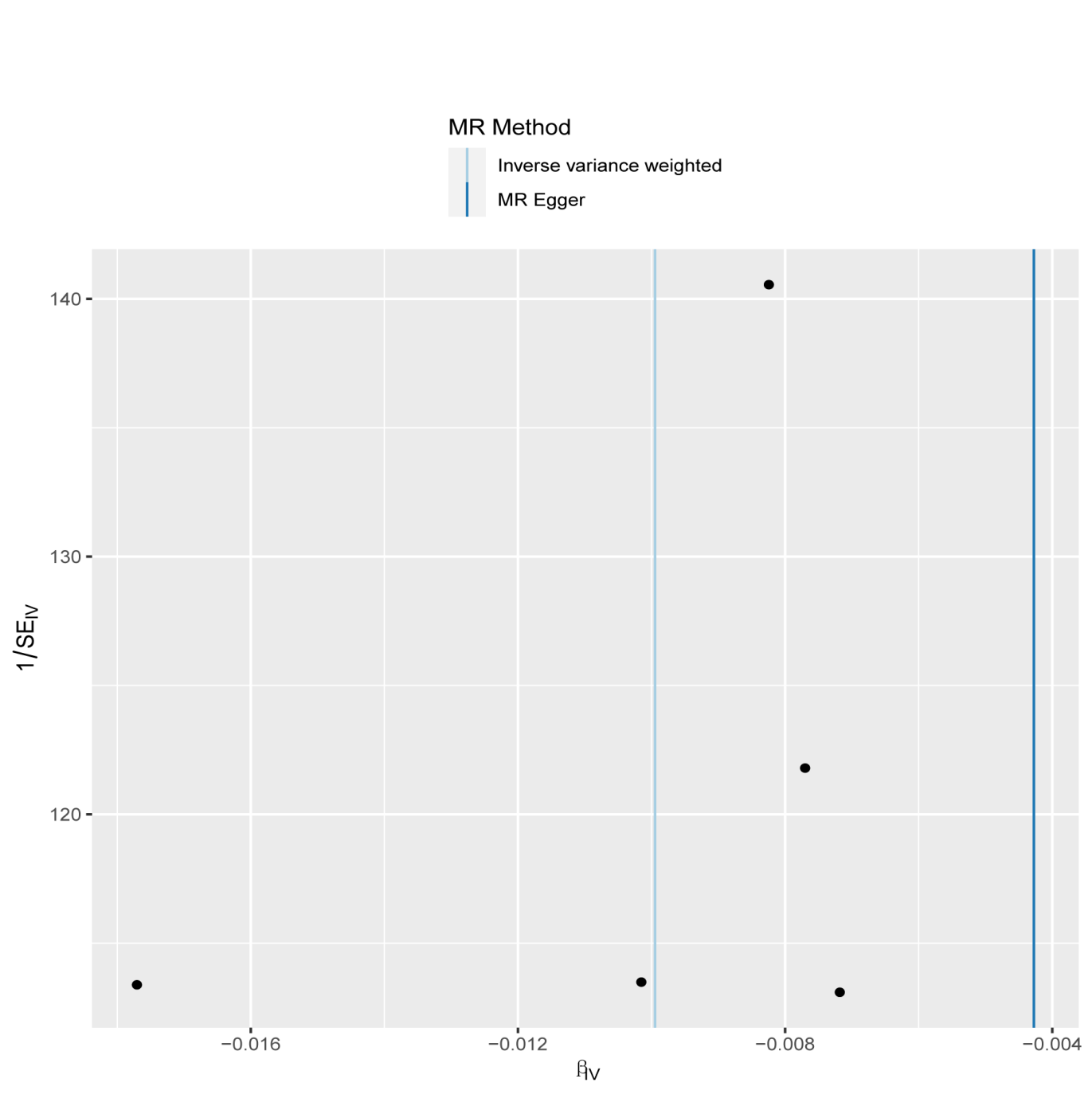


**S4. Scatter plot of the causal effect of family Clostridiales vadin BB60 group id.11286 (A), genus Clostridium innocuum group id.14397 (B), genus Desulfovibrio id.3173 (C), genus Flavonifractor id.2059 (D), genus Olsenella id.822 (E), genus Parabacteroides id.954 (F) and genus Senegalimassilia id.11160 (G) on hypertension.**

**A.**
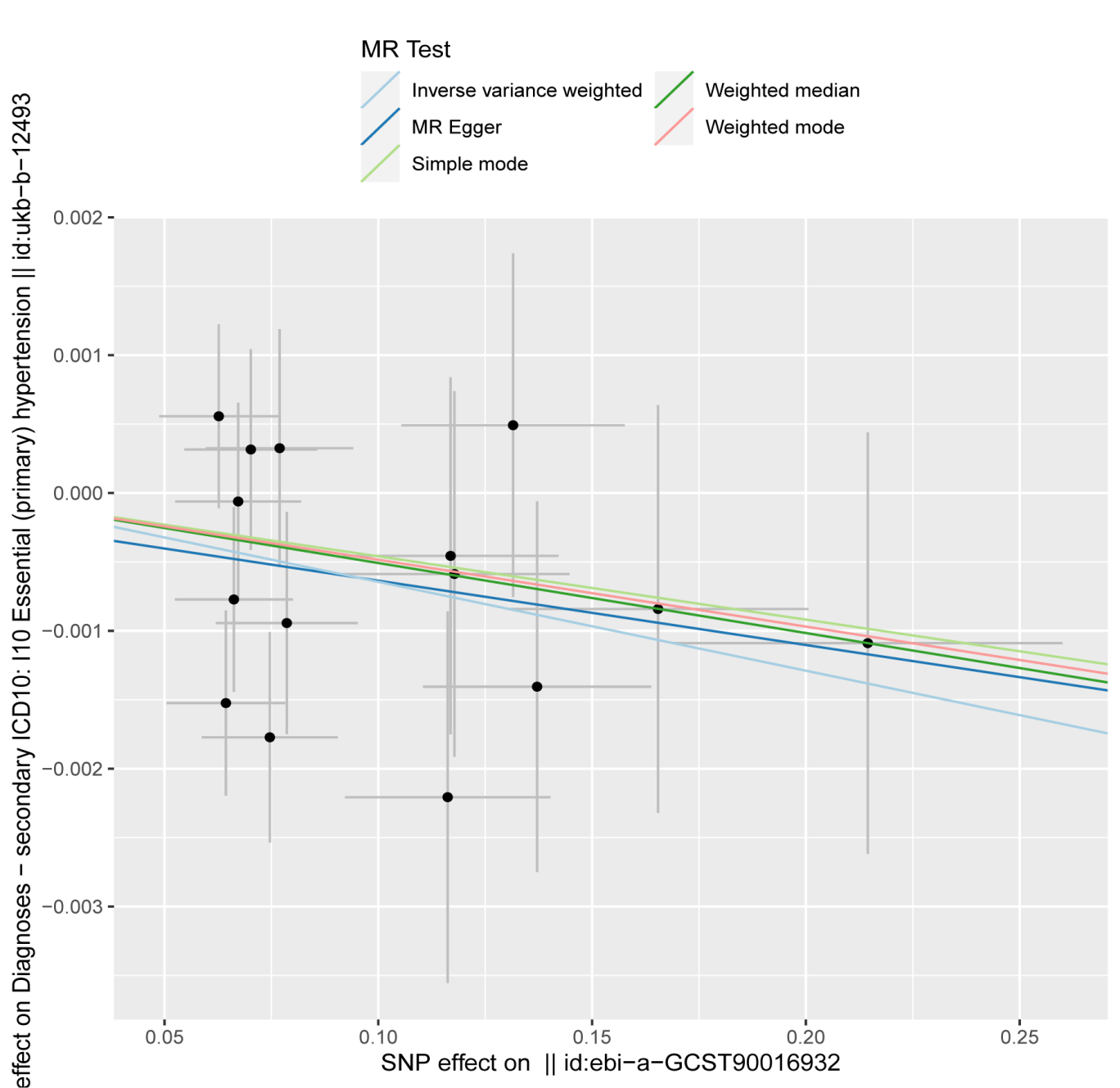
**B.**
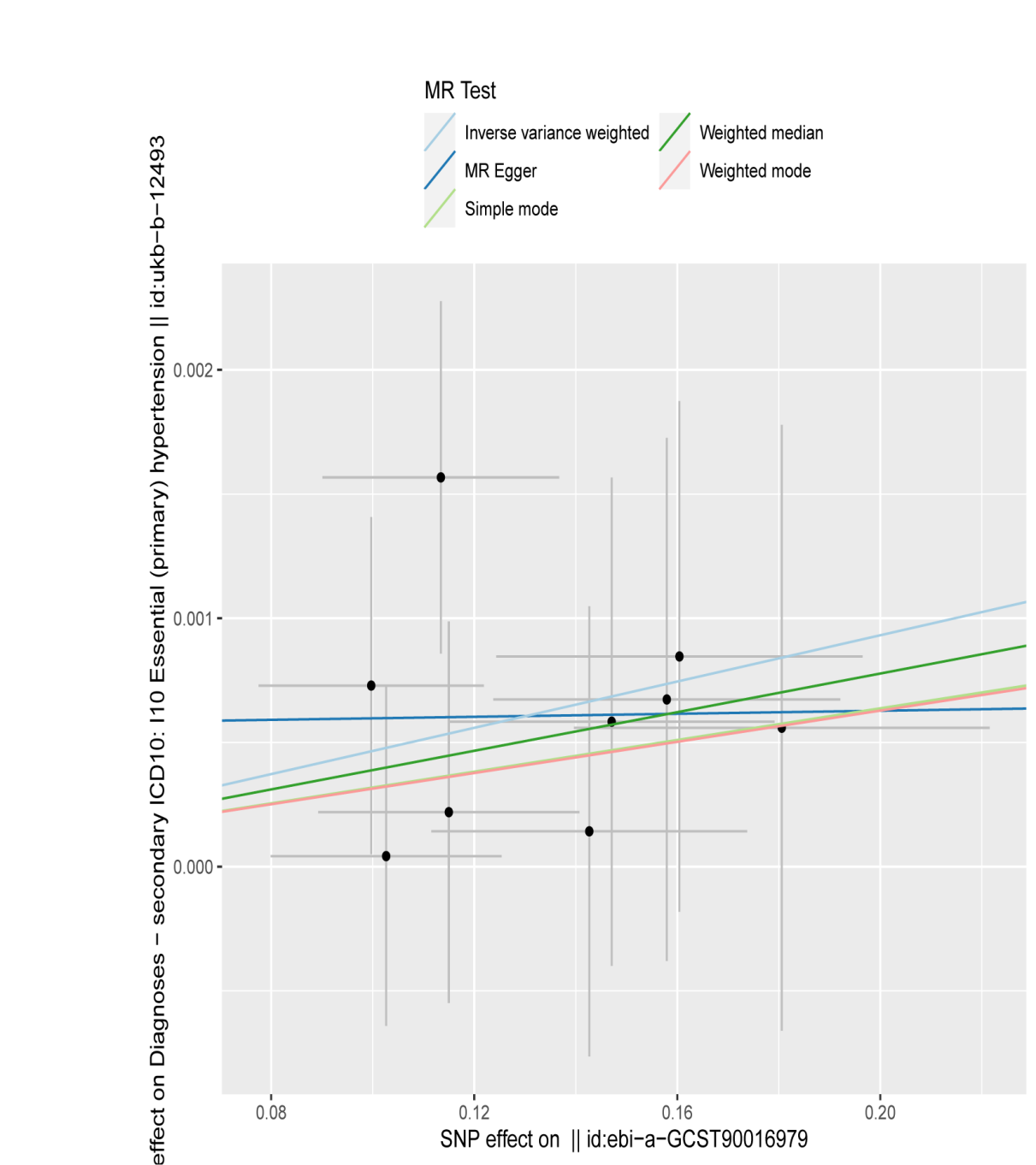
**C.**
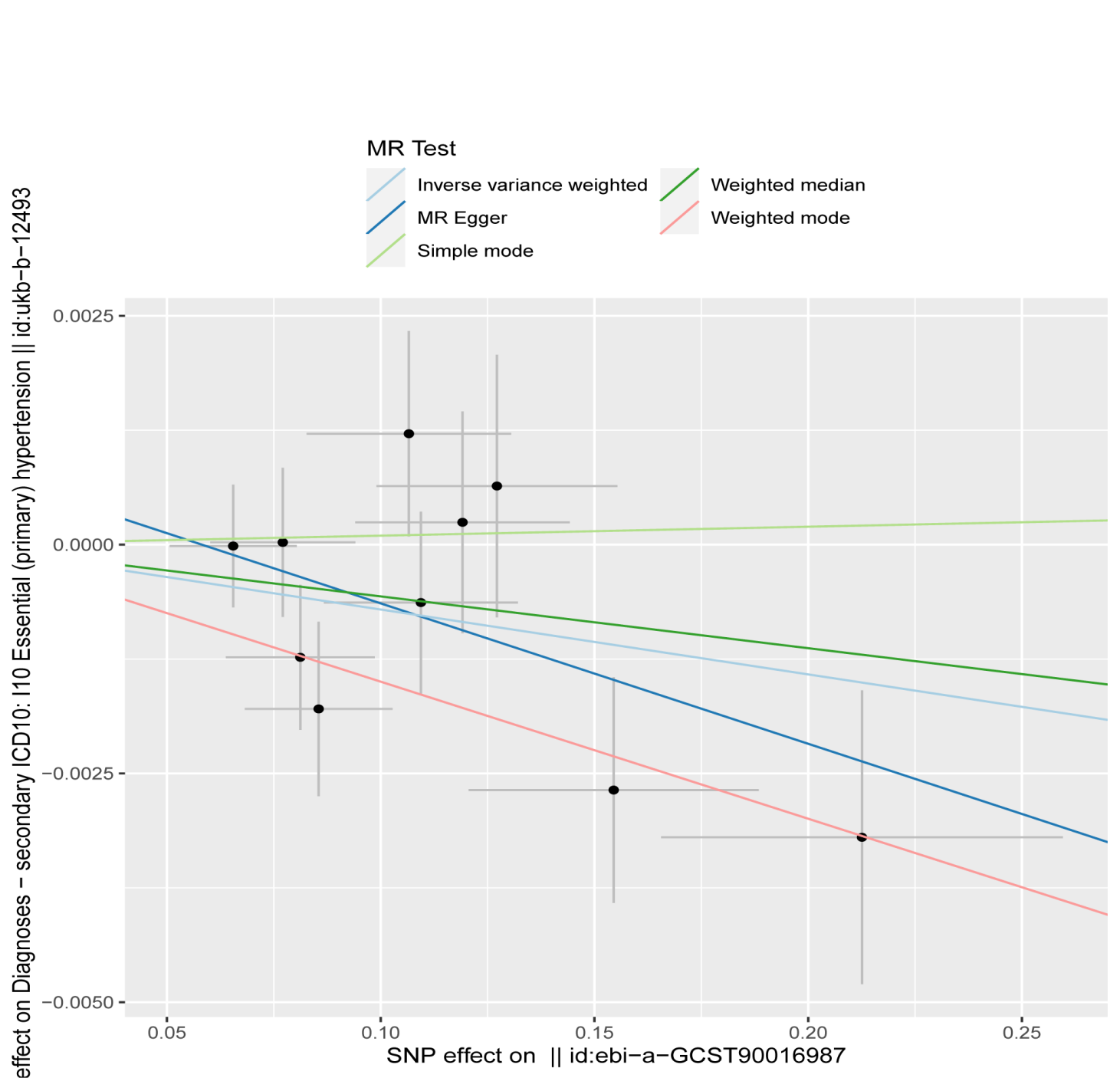


**D.**
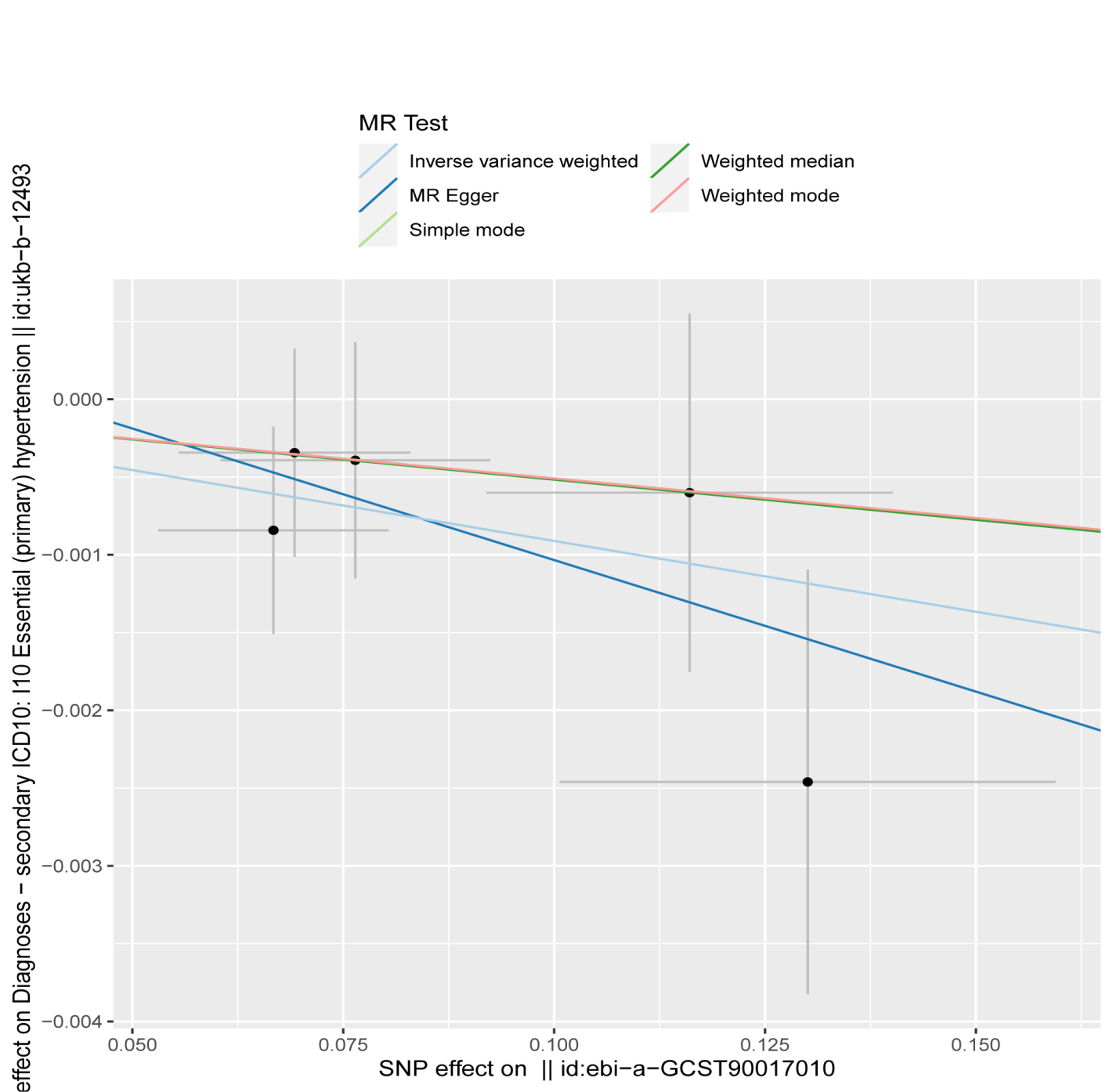
**E.**
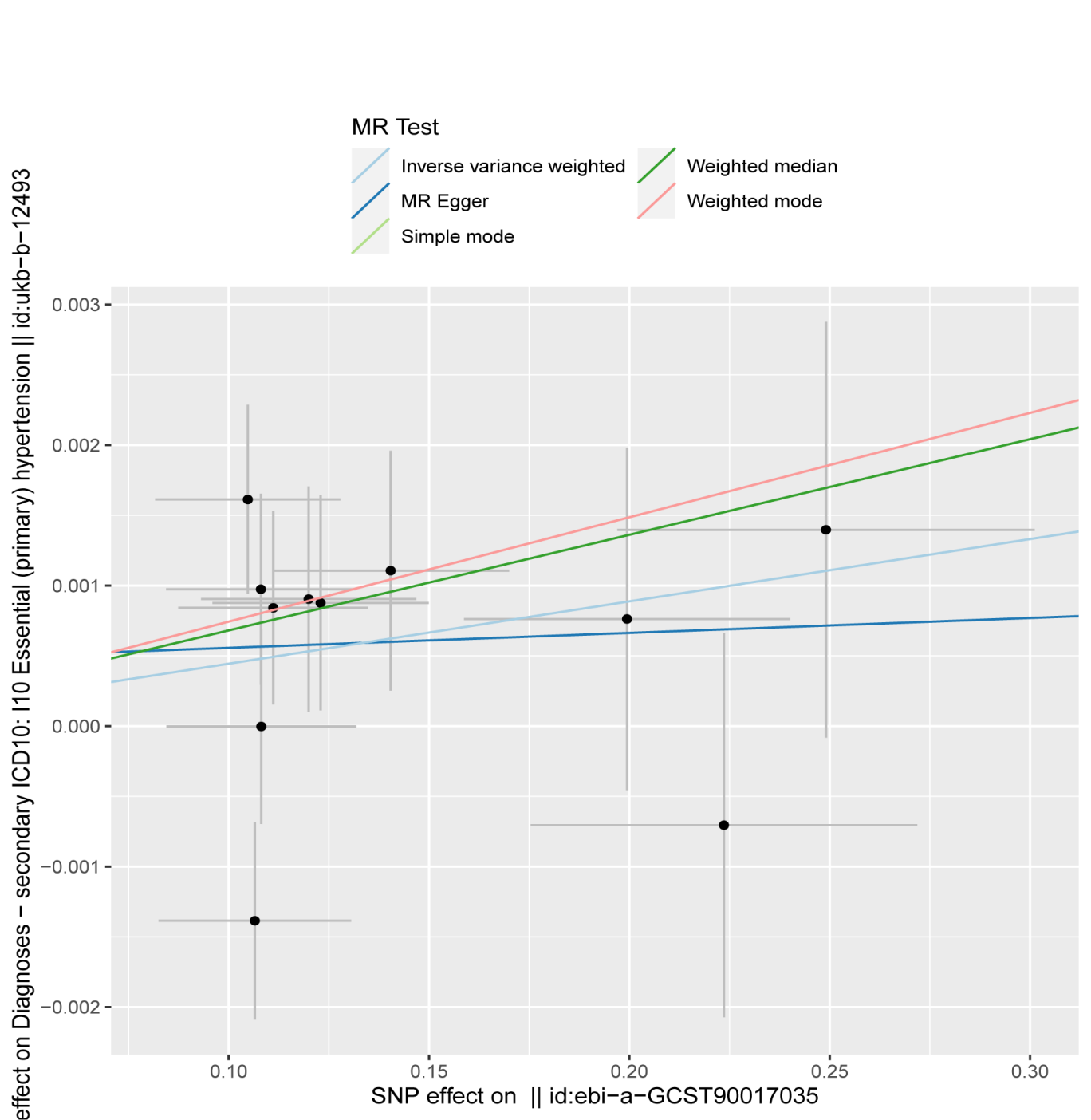
**F.**
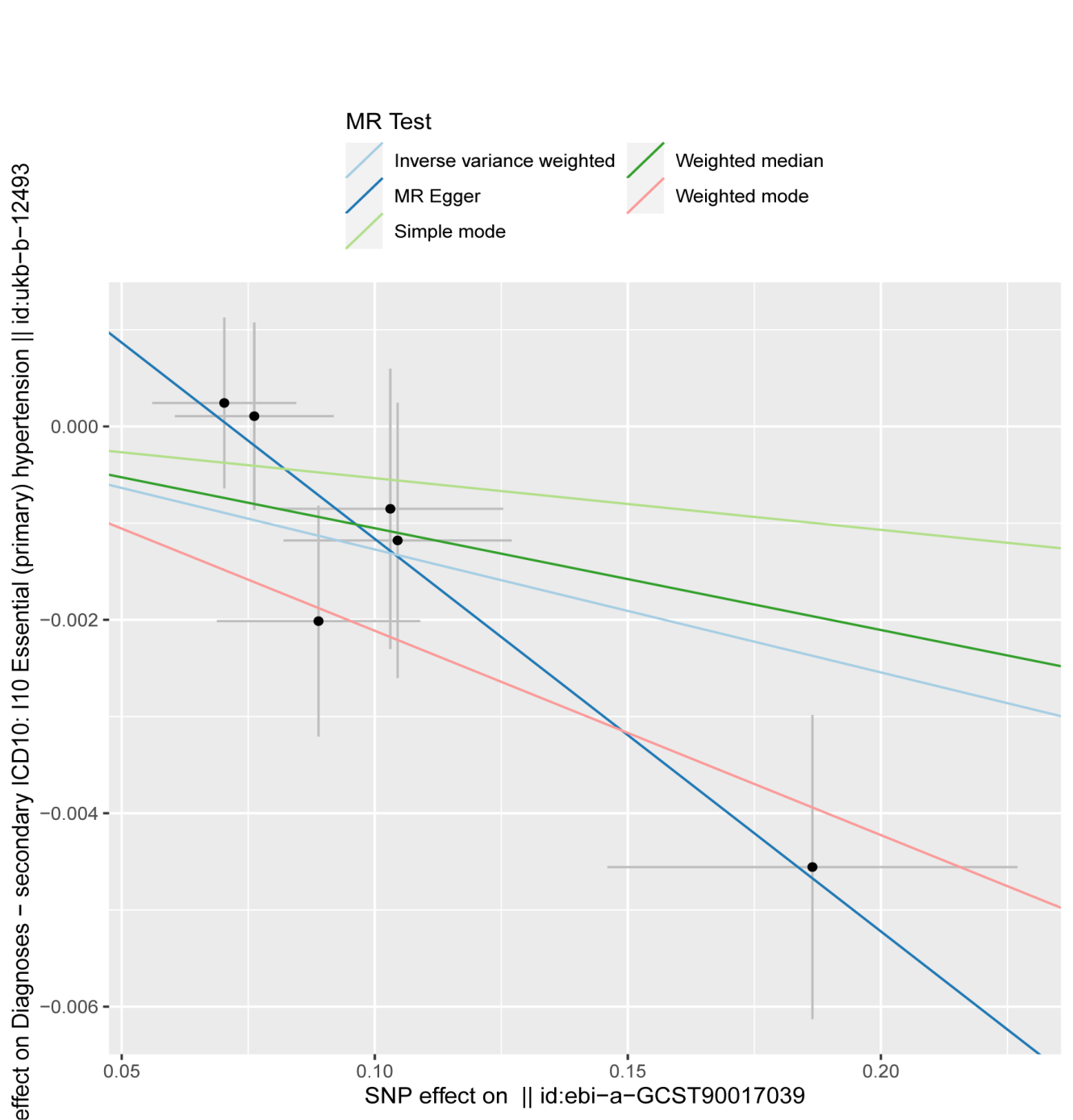


**G.**

**S5. Leave-one-out analysis of the causal effect of family Clostridiales vadin BB60 group id.11286 (A), genus Clostridium innocuum group id.14397 (B), genus Desulfovibrio id.3173 (C), genus Flavonifractor id.2059 (D), genus Olsenella id.822 (E), genus Parabacteroides id.954 (F) and genus Senegalimassilia id.11160 (G) on hypertension.**

**A.
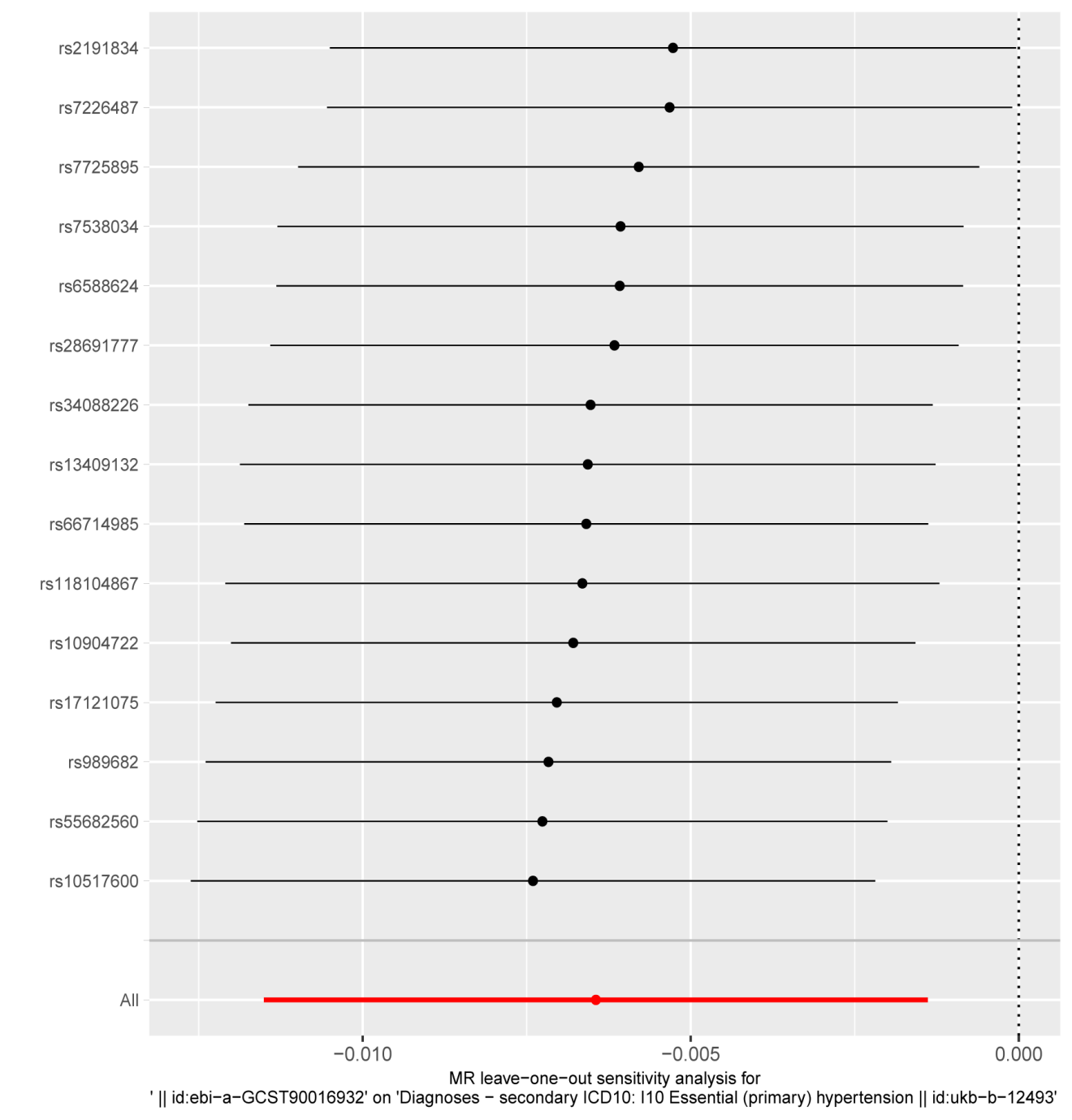
B.
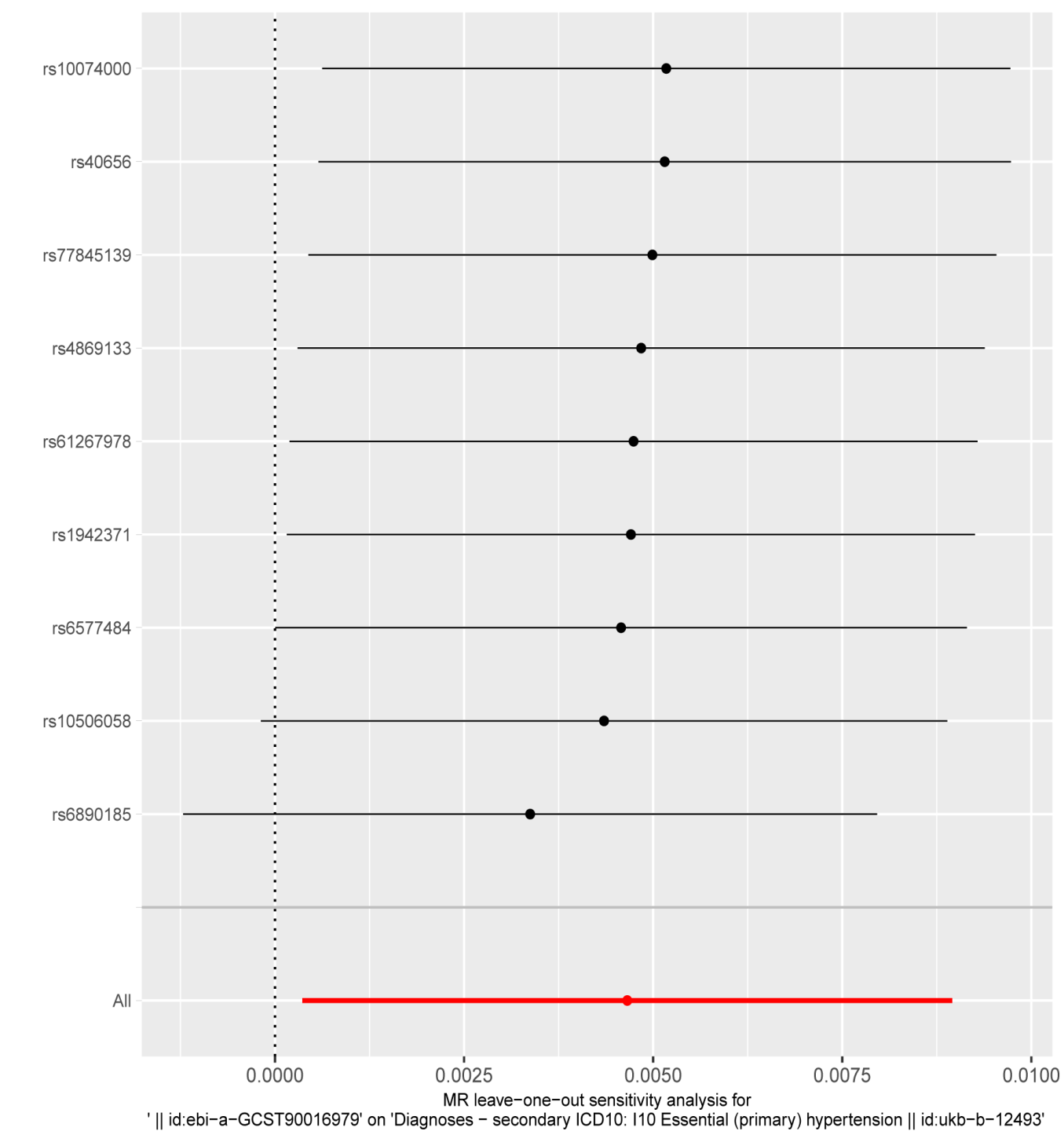
C.
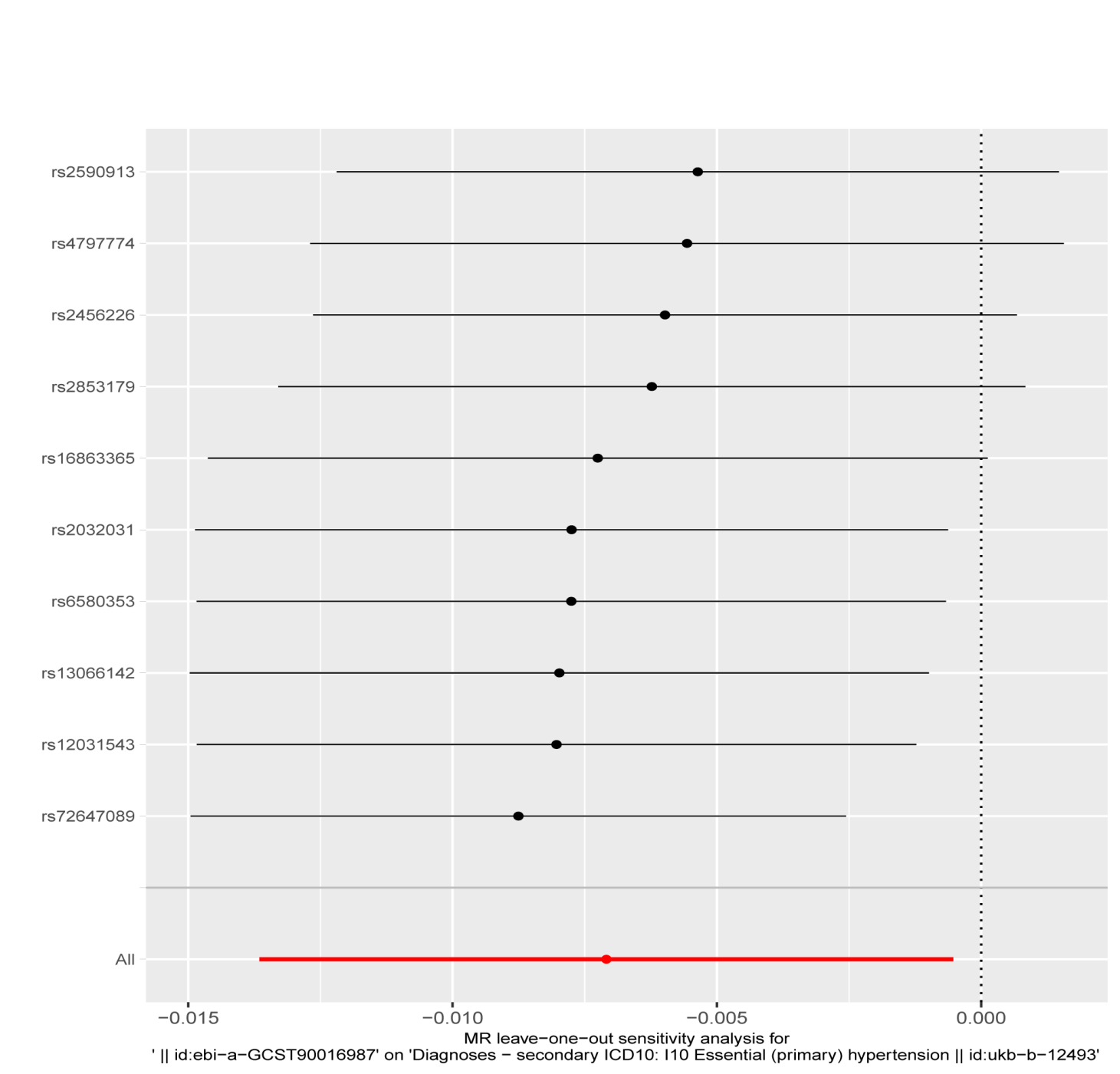
**

**D.**
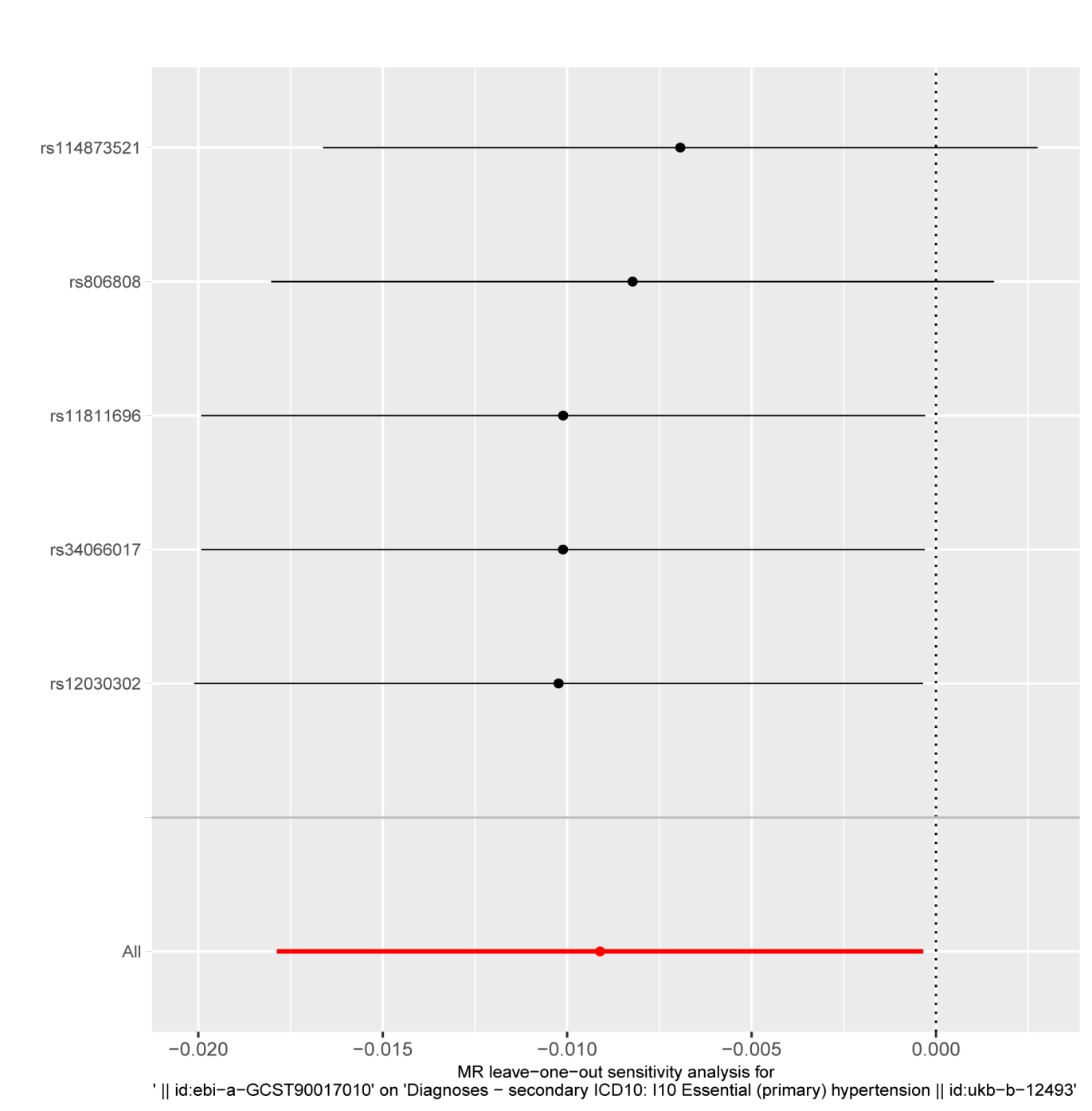
**E.**
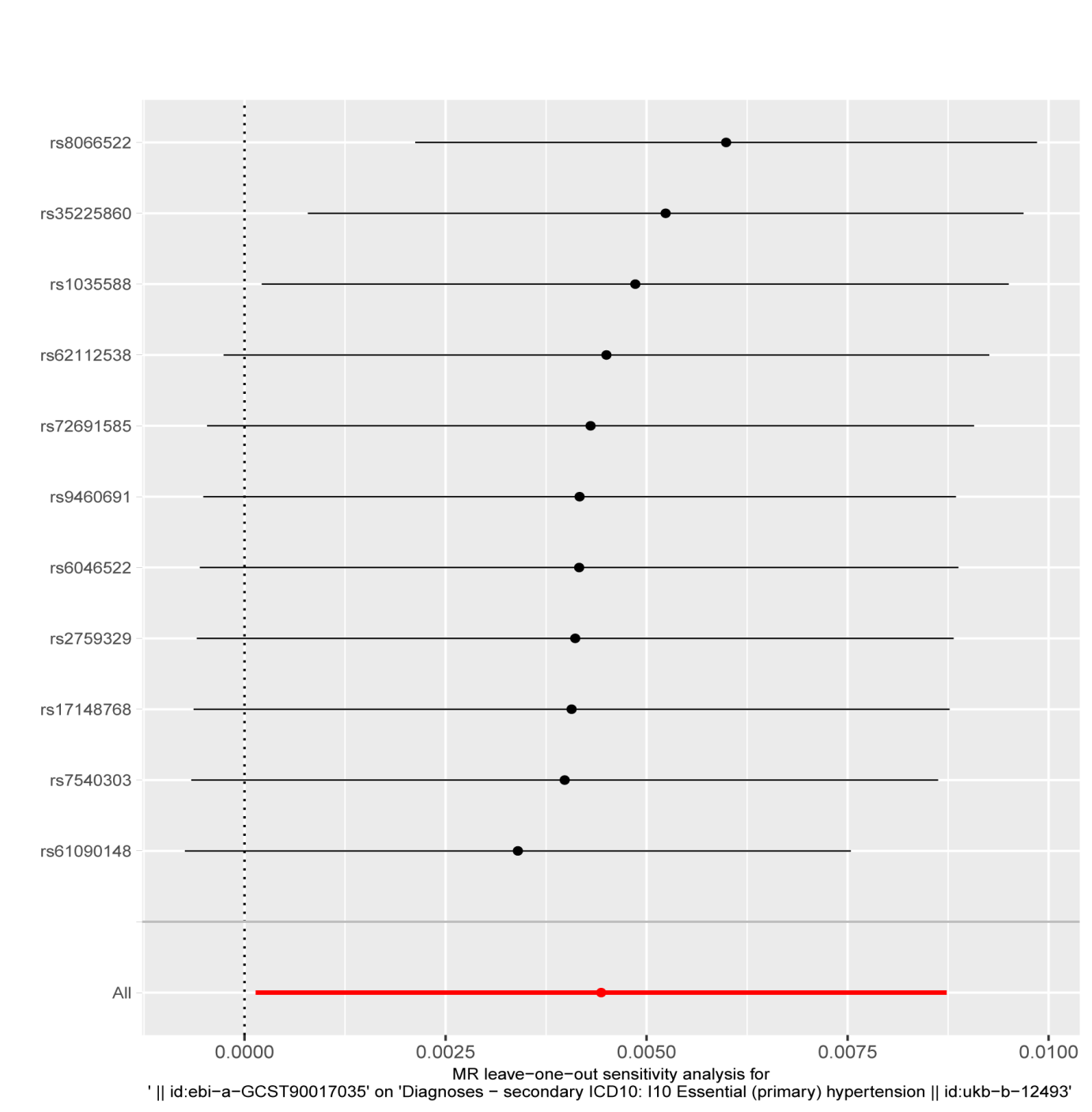
**F.**
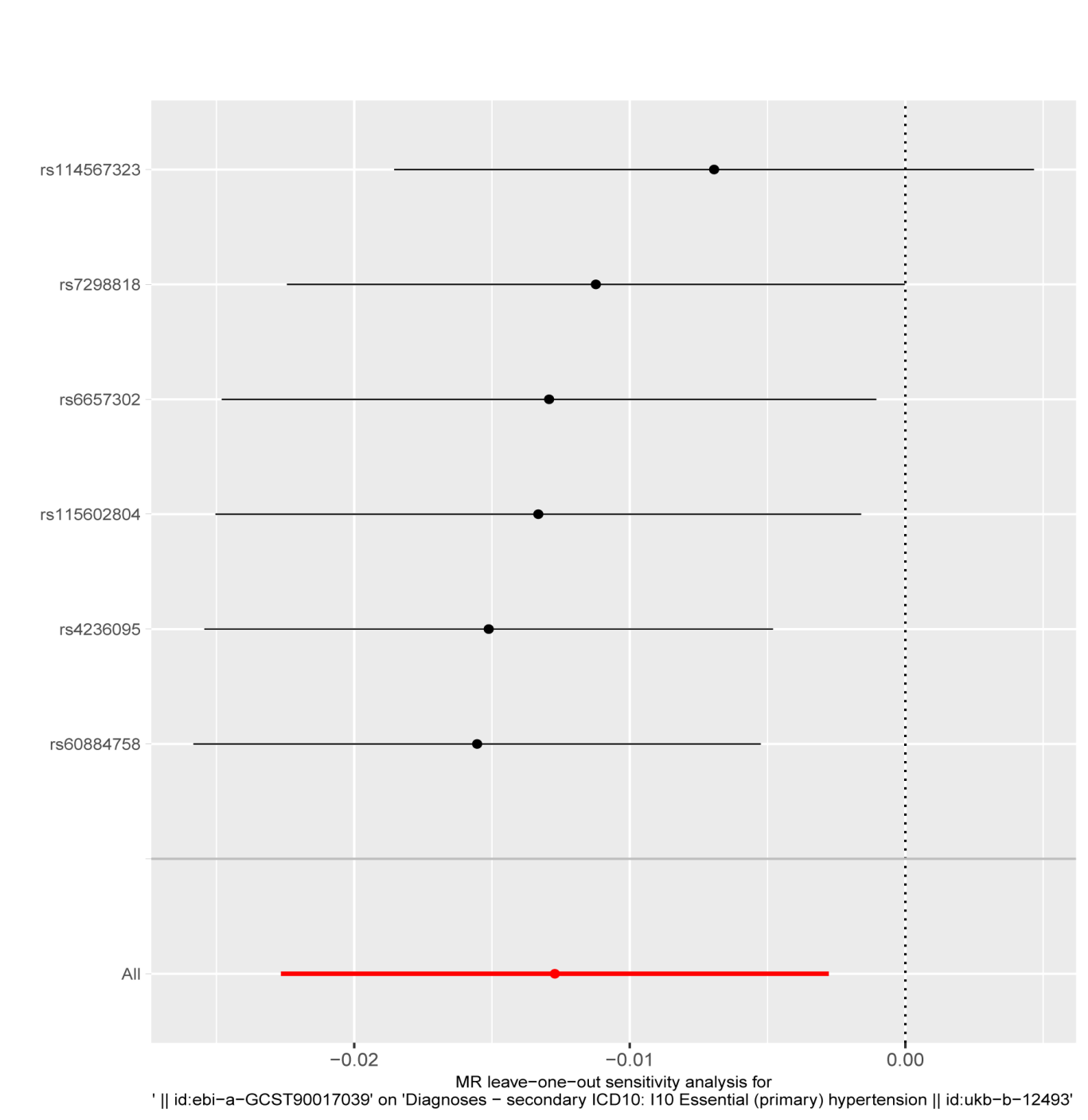


**G.**
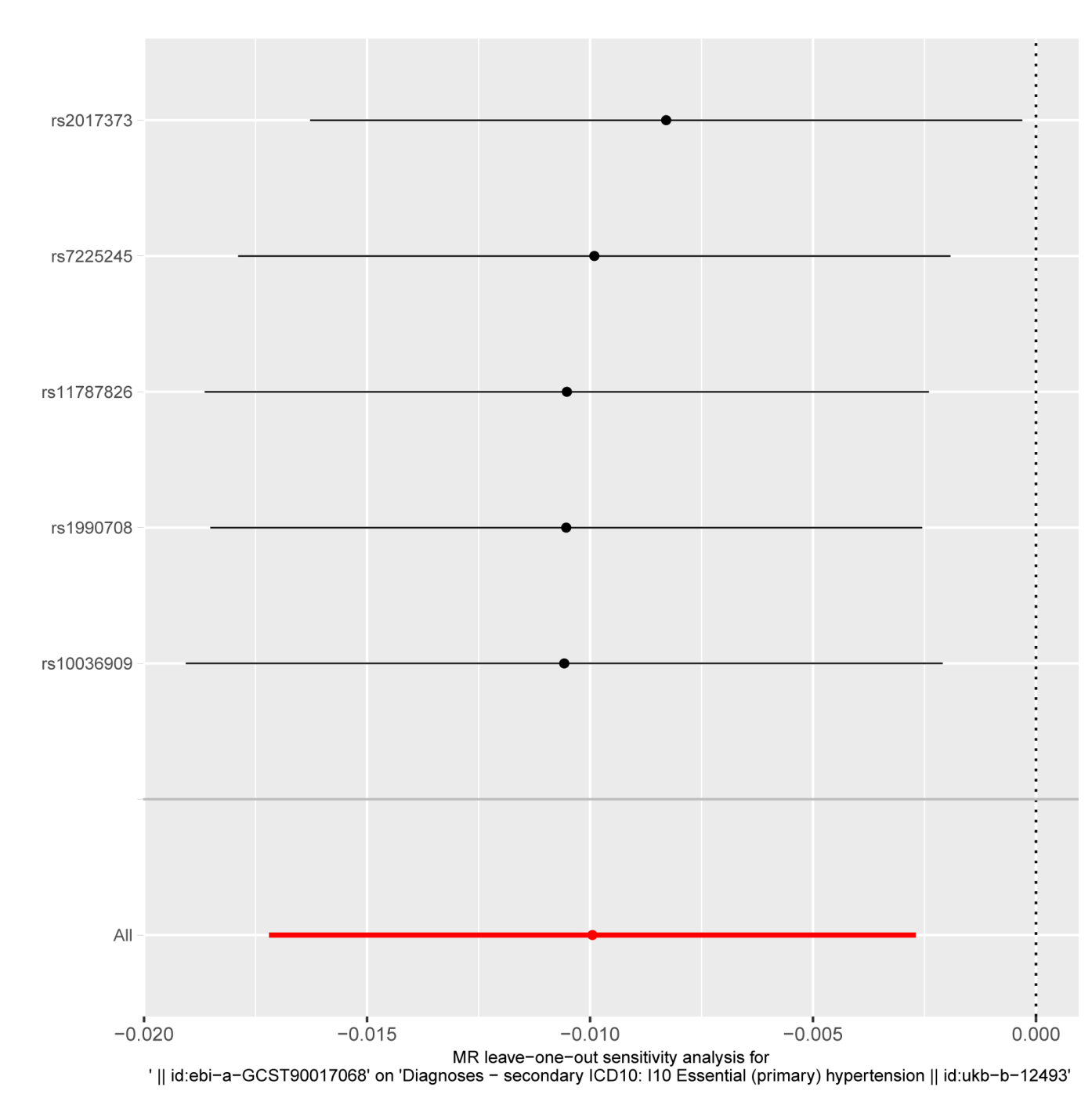


**S6. MR results of causal links between gut microbiome and hypertension.**

| exposure | id.exposure | outcome | id.outcome | method | nsnp | b | se | pval | lo_ci | up_ci |
| --- | --- | --- | --- | --- | --- | --- | --- | --- | --- | --- |
| family Clostridiales vadin BB60 group id.11286 | ebi-a-GCST90016932 | hypertension | ukb-b-12493 | MR Egger | 15 | -0.004666763 | 0.00727956 | 0.532611786 | -0.0189347 | 0.009601175 |
| family Clostridiales vadin BB60 group id.11286 | ebi-a-GCST90016932 | hypertension | ukb-b-12493 | Weighted median | 15 | -0.005079555 | 0.003548194 | 0.152261524 | -0.012034014 | 0.001874905 |
| family Clostridiales vadin BB60 group id.11286 | ebi-a-GCST90016932 | hypertension | ukb-b-12493 | Inverse variance weighted | 15 | -0.00644567 | 0.002581748 | 0.01253797 | -0.011505897 | -0.001385444 |
| family Clostridiales vadin BB60 group id.11286 | ebi-a-GCST90016932 | hypertension | ukb-b-12493 | Simple mode | 15 | -0.004593302 | 0.00664286 | 0.500580131 | -0.017613307 | 0.008426703 |
| family Clostridiales vadin BB60 group id.11286 | ebi-a-GCST90016932 | hypertension | ukb-b-12493 | Weighted mode | 15 | -0.004845739 | 0.005796841 | 0.417234924 | -0.016207547 | 0.006516068 |
| genus Clostridium innocuum group id.14397 | ebi-a-GCST90016979 | hypertension | ukb-b-12493 | MR Egger | 9 | 0.000304991 | 0.011164353 | 0.978968377 | -0.021577141 | 0.022187123 |
| genus Clostridium innocuum group id.14397 | ebi-a-GCST90016979 | hypertension | ukb-b-12493 | Weighted median | 9 | 0.003887166 | 0.002928967 | 0.184460513 | -0.00185361 | 0.009627941 |
| genus Clostridium innocuum group id.14397 | ebi-a-GCST90016979 | hypertension | ukb-b-12493 | Inverse variance weighted | 9 | 0.004657847 | 0.00219208 | 0.033598896 | 0.000361371 | 0.008954324 |
| genus Clostridium innocuum group id.14397 | ebi-a-GCST90016979 | hypertension | ukb-b-12493 | Simple mode | 9 | 0.003187528 | 0.004049991 | 0.453937719 | -0.004750454 | 0.01112551 |
| genus Clostridium innocuum group id.14397 | ebi-a-GCST90016979 | hypertension | ukb-b-12493 | Weighted mode | 9 | 0.003140496 | 0.003974354 | 0.452201325 | -0.004649238 | 0.010930229 |
| genus Desulfovibrio id.3173 | ebi-a-GCST90016987 | hypertension | ukb-b-12493 | MR Egger | 10 | -0.015338501 | 0.010384301 | 0.17790138 | -0.03569173 | 0.005014729 |
| genus Desulfovibrio id.3173 | ebi-a-GCST90016987 | hypertension | ukb-b-12493 | Weighted median | 10 | -0.005658773 | 0.00432035 | 0.190265139 | -0.014126659 | 0.002809112 |
| genus Desulfovibrio id.3173 | ebi-a-GCST90016987 | hypertension | ukb-b-12493 | Inverse variance weighted | 10 | -0.007090343 | 0.003348935 | 0.034243426 | -0.013654256 | -0.000526431 |
| genus Desulfovibrio id.3173 | ebi-a-GCST90016987 | hypertension | ukb-b-12493 | Simple mode | 10 | 0.000977257 | 0.00807604 | 0.906344099 | -0.014851782 | 0.016806295 |
| genus Desulfovibrio id.3173 | ebi-a-GCST90016987 | hypertension | ukb-b-12493 | Weighted mode | 10 | -0.014974468 | 0.007816302 | 0.087644104 | -0.03029442 | 0.000345483 |
| genus Flavonifractor id.2059 | ebi-a-GCST90017010 | hypertension | ukb-b-12493 | MR Egger | 5 | -0.016912785 | 0.018264307 | 0.422745381 | -0.052710826 | 0.018885257 |
| genus Flavonifractor id.2059 | ebi-a-GCST90017010 | hypertension | ukb-b-12493 | Weighted median | 5 | -0.005166209 | 0.005939308 | 0.384391394 | -0.016807253 | 0.006474835 |
| genus Flavonifractor id.2059 | ebi-a-GCST90017010 | hypertension | ukb-b-12493 | Inverse variance weighted | 5 | -0.009107448 | 0.00447048 | 0.041625829 | -0.017869589 | -0.000345307 |
| genus Flavonifractor id.2059 | ebi-a-GCST90017010 | hypertension | ukb-b-12493 | Simple mode | 5 | -0.005090494 | 0.007173439 | 0.517109639 | -0.019150434 | 0.008969445 |
| genus Flavonifractor id.2059 | ebi-a-GCST90017010 | hypertension | ukb-b-12493 | Weighted mode | 5 | -0.005090494 | 0.007811628 | 0.55016935 | -0.020401286 | 0.010220297 |
| genus Olsenella id.822 | ebi-a-GCST90017035 | hypertension | ukb-b-12493 | MR Egger | 11 | 0.001061142 | 0.008667278 | 0.905248114 | -0.015926723 | 0.018049006 |
| genus Olsenella id.822 | ebi-a-GCST90017035 | hypertension | ukb-b-12493 | Weighted median | 11 | 0.006805063 | 0.002601926 | 0.00891243 | 0.001705289 | 0.011904837 |
| genus Olsenella id.822 | ebi-a-GCST90017035 | hypertension | ukb-b-12493 | Inverse variance weighted | 11 | 0.004435551 | 0.00219184 | 0.043004563 | 0.000139545 | 0.008731557 |
| genus Olsenella id.822 | ebi-a-GCST90017035 | hypertension | ukb-b-12493 | Simple mode | 11 | 0.007428939 | 0.003950492 | 0.089448609 | -0.000314024 | 0.015171903 |
| genus Olsenella id.822 | ebi-a-GCST90017035 | hypertension | ukb-b-12493 | Weighted mode | 11 | 0.007428939 | 0.003788767 | 0.078334704 | 2.96E-06 | 0.014854922 |
| genus Parabacteroides id.954 | ebi-a-GCST90017039 | hypertension | ukb-b-12493 | MR Egger | 6 | -0.040591353 | 0.014710359 | 0.050882392 | -0.069423657 | -0.01175905 |
| genus Parabacteroides id.954 | ebi-a-GCST90017039 | hypertension | ukb-b-12493 | Weighted median | 6 | -0.010526165 | 0.006917795 | 0.128107603 | -0.024085044 | 0.003032714 |
| genus Parabacteroides id.954 | ebi-a-GCST90017039 | hypertension | ukb-b-12493 | Inverse variance weighted | 6 | -0.012719793 | 0.005071784 | 0.012143297 | -0.022660489 | -0.002779097 |
| genus Parabacteroides id.954 | ebi-a-GCST90017039 | hypertension | ukb-b-12493 | Simple mode | 6 | -0.005347406 | 0.011568632 | 0.663330737 | -0.028021925 | 0.017327114 |
| genus Parabacteroides id.954 | ebi-a-GCST90017039 | hypertension | ukb-b-12493 | Weighted mode | 6 | -0.02113036 | 0.012011664 | 0.138874709 | -0.044673221 | 0.002412501 |
| genus Senegalimassilia id.11160 | ebi-a-GCST90017068 | hypertension | ukb-b-12493 | MR Egger | 5 | -0.004275426 | 0.012562298 | 0.75603975 | -0.028897531 | 0.020346679 |
| genus Senegalimassilia id.11160 | ebi-a-GCST90017068 | hypertension | ukb-b-12493 | Weighted median | 5 | -0.008179203 | 0.004683073 | 0.080716079 | -0.017358025 | 0.000999619 |
| genus Senegalimassilia id.11160 | ebi-a-GCST90017068 | hypertension | ukb-b-12493 | Inverse variance weighted | 5 | -0.009947169 | 0.003698263 | 0.007151916 | -0.017195765 | -0.002698573 |
| genus Senegalimassilia id.11160 | ebi-a-GCST90017068 | hypertension | ukb-b-12493 | Simple mode | 5 | -0.007790117 | 0.006127535 | 0.272508366 | -0.019800085 | 0.004219852 |
| genus Senegalimassilia id.11160 | ebi-a-GCST90017068 | hypertension | ukb-b-12493 | Weighted mode | 5 | -0.007855419 | 0.005829123 | 0.249051402 | -0.019280499 | 0.003569662 |

**S7. Funnel plot of the causal effect of Interleukin-2 receptor subunit alpha levels (A), Interleukin-11 receptor subunit alpha (B), Interleukin-23 (C), Interleukin-1 receptor type 2 (D), Interleukin-27 (E), Interleukin-31 (F) and Interleukin 1 receptor like 1 (G) on hypertension.**

1. **
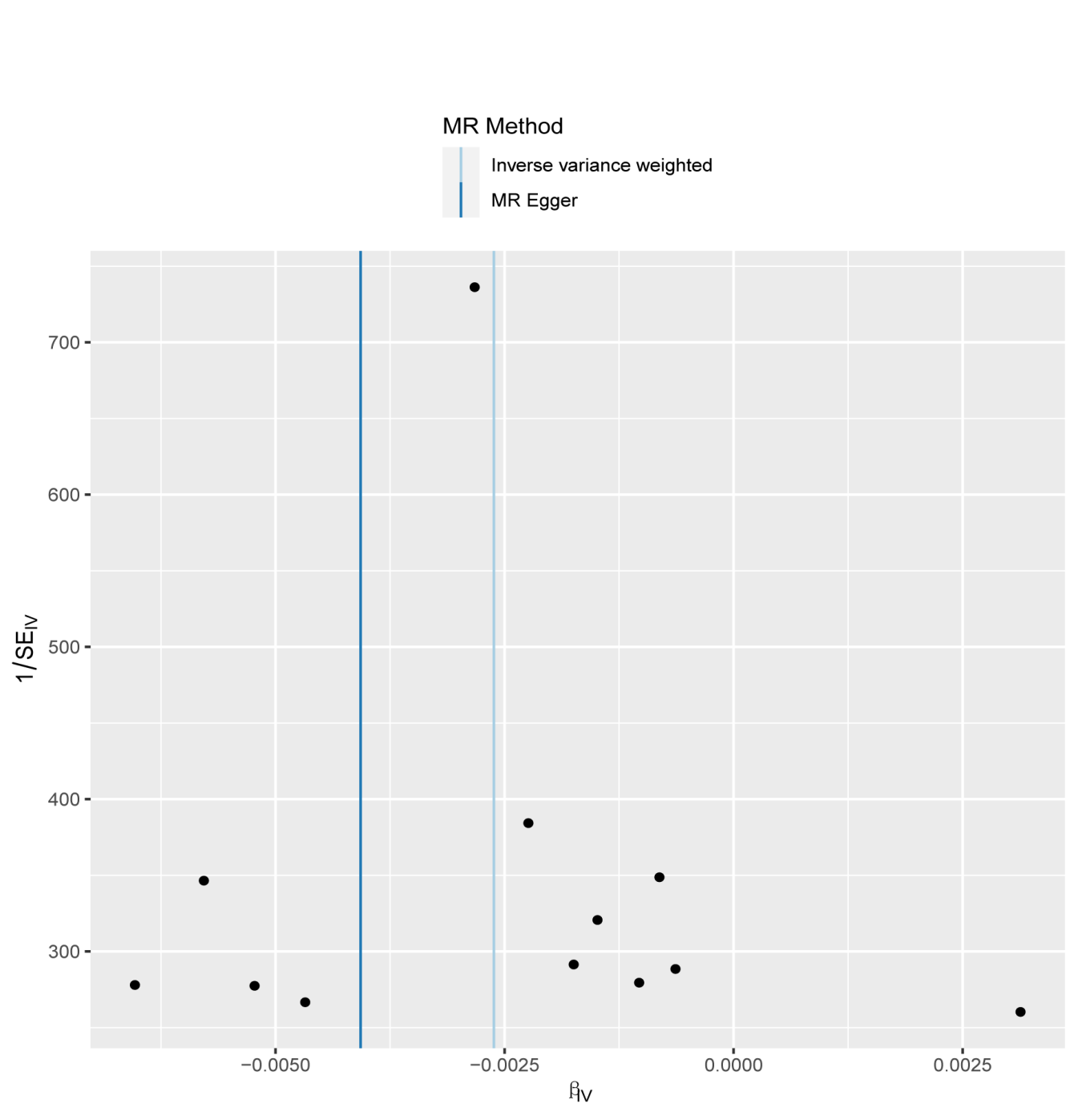
B.
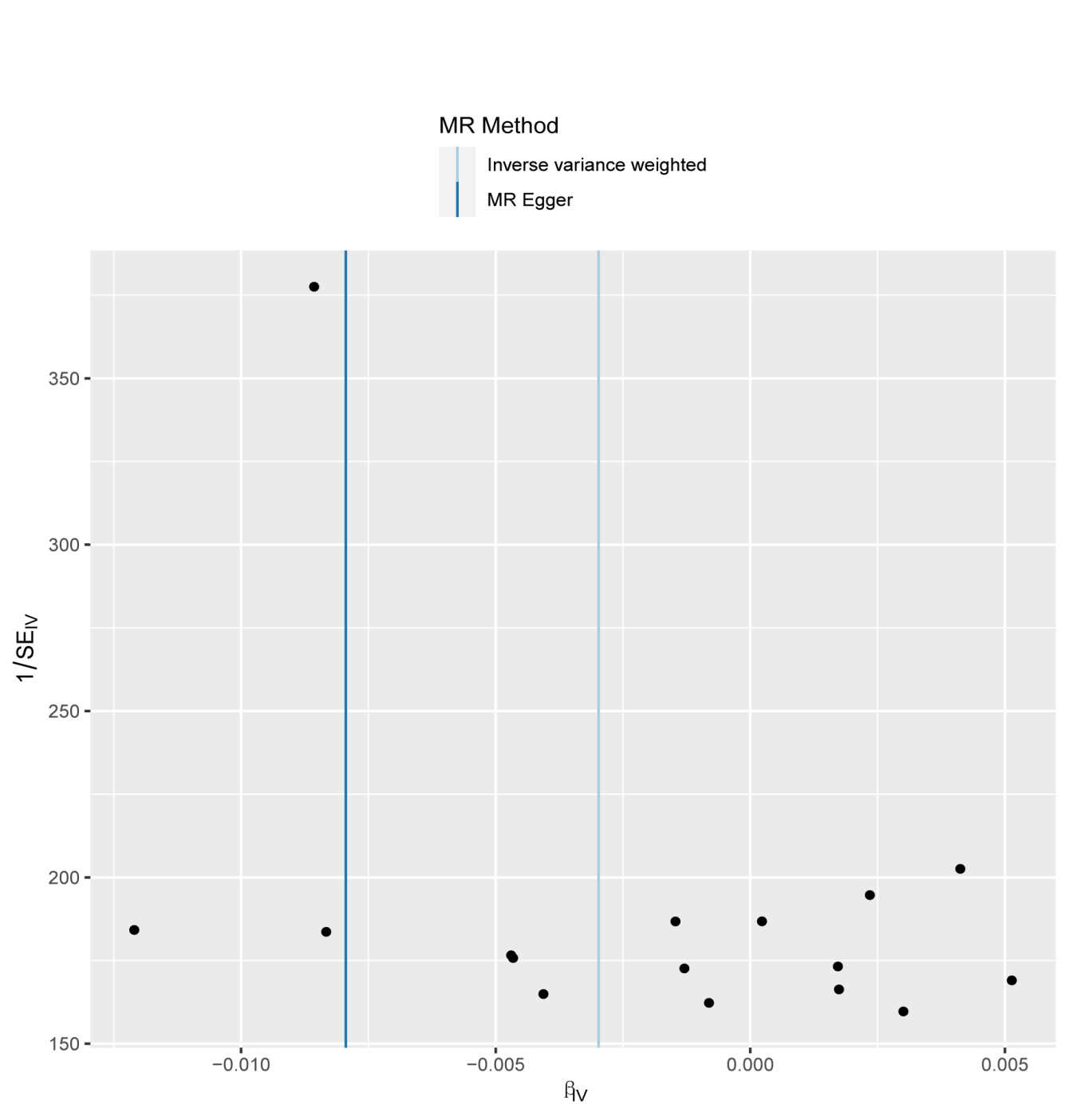
C.
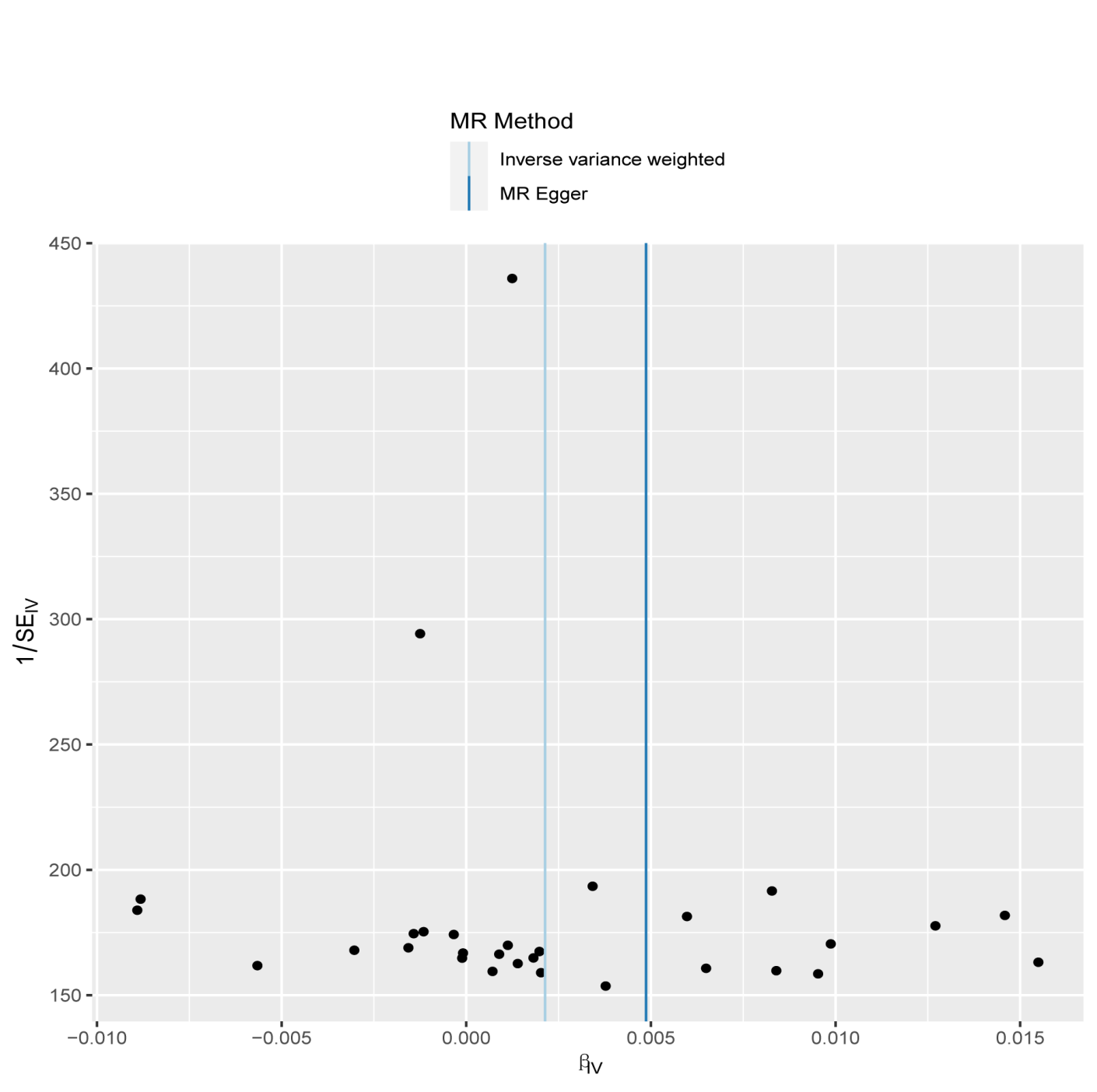
**
2. **
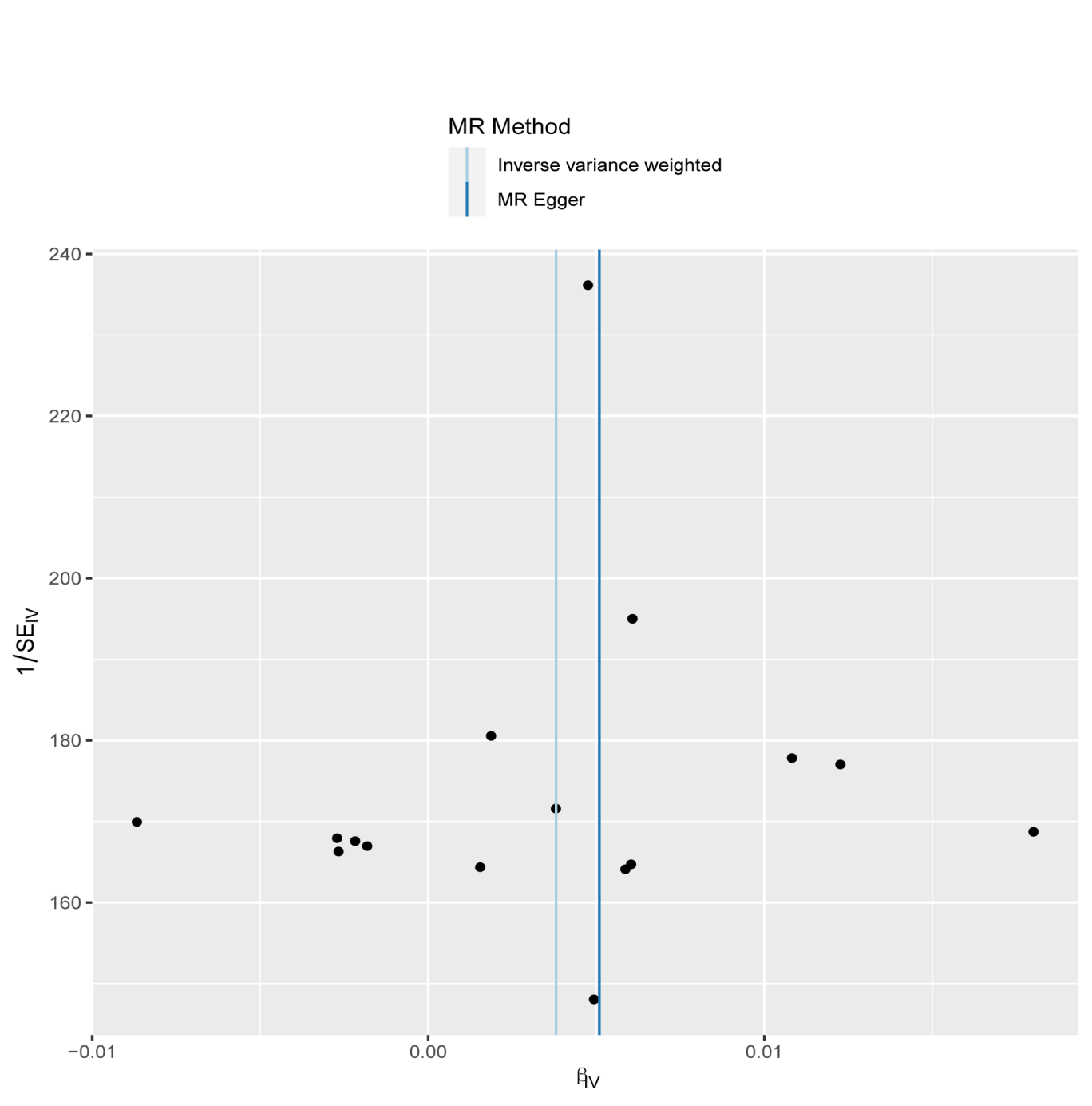
E.
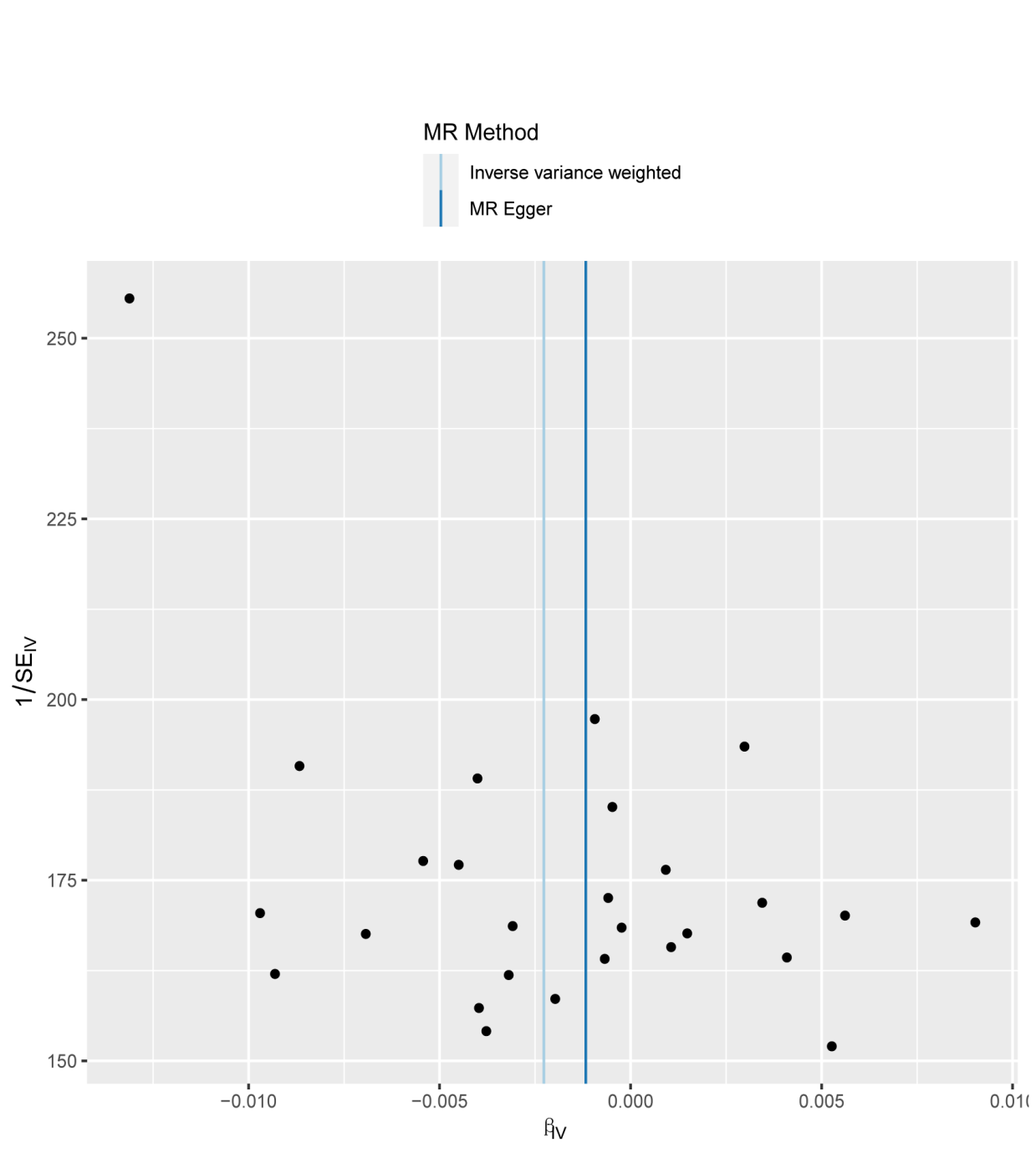
F.
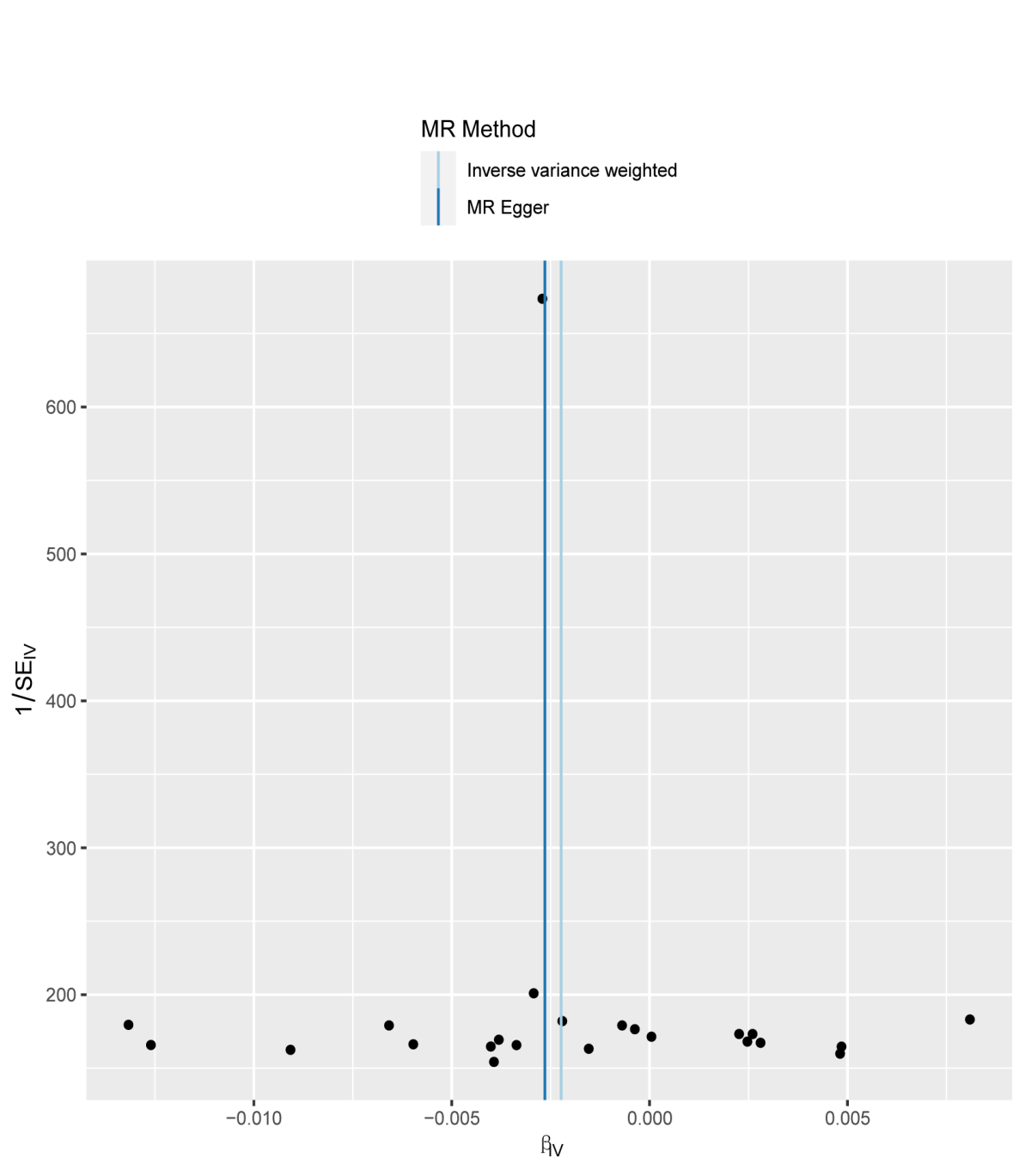
**

**G.
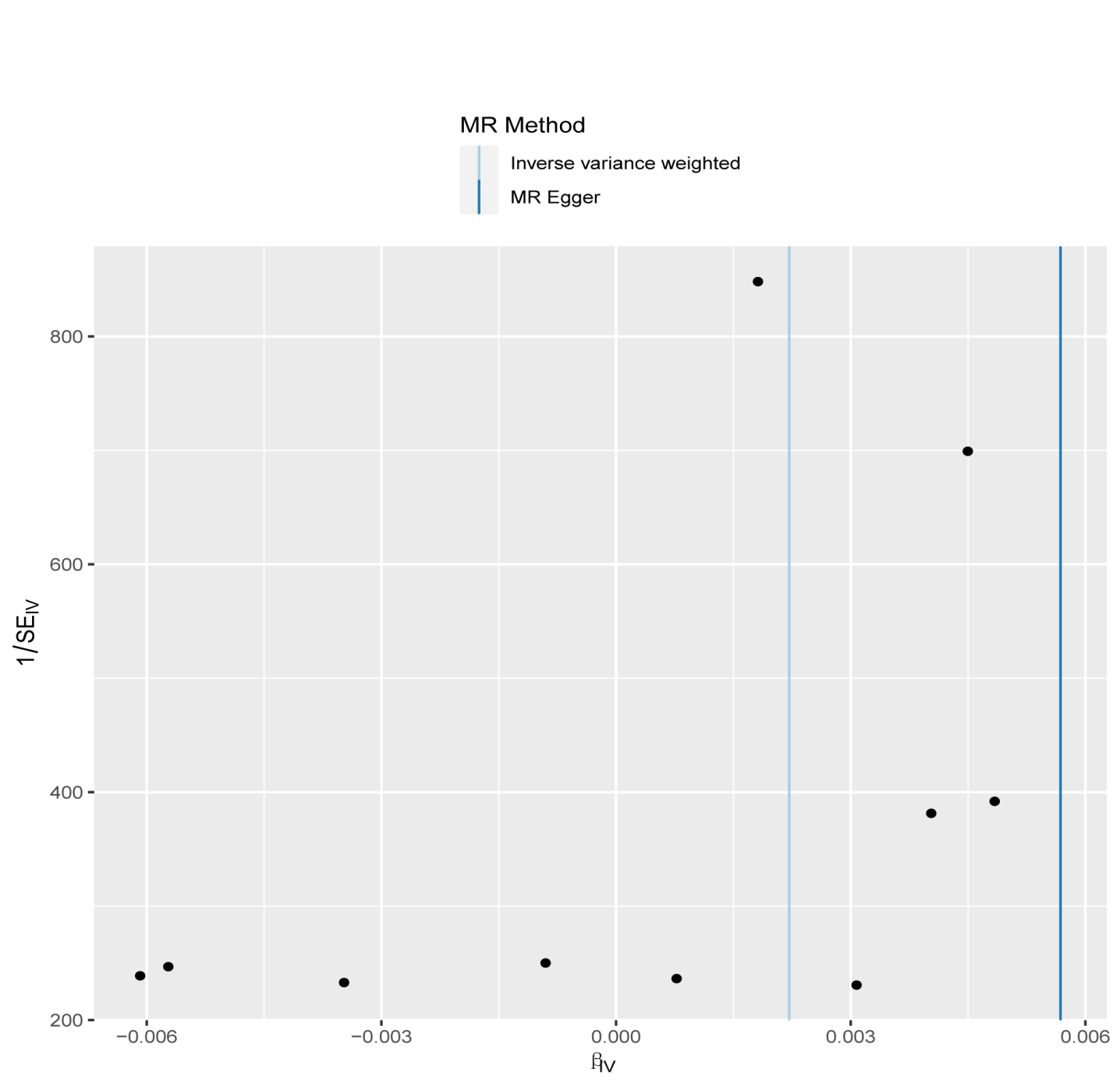
**

**S8. Scatter plot of the causal effect of Interleukin-2 receptor subunit alpha levels (A), Interleukin-11 receptor subunit alpha (B), Interleukin-23 (C), Interleukin-1 receptor type 2 (D), Interleukin-27 (E), Interleukin-31 (F) and Interleukin 1 receptor like 1 (G) on hypertension.**

1. **B.C.
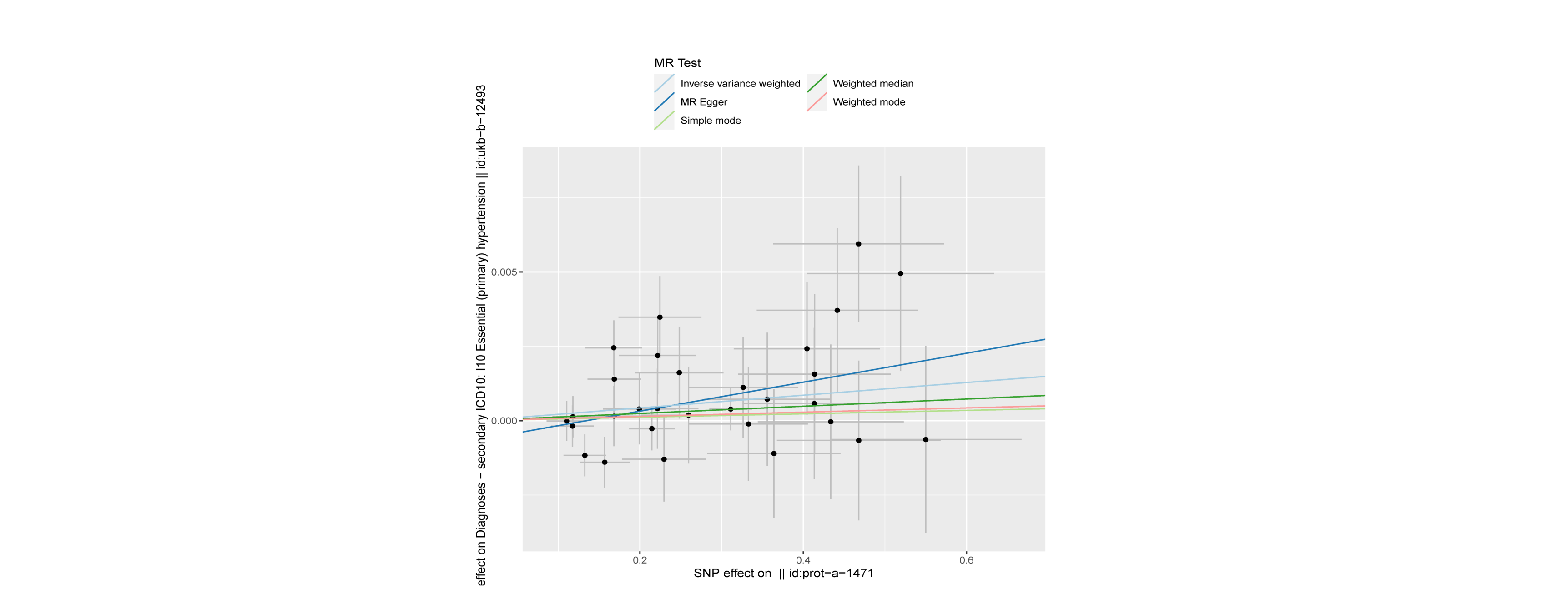
**

**D.
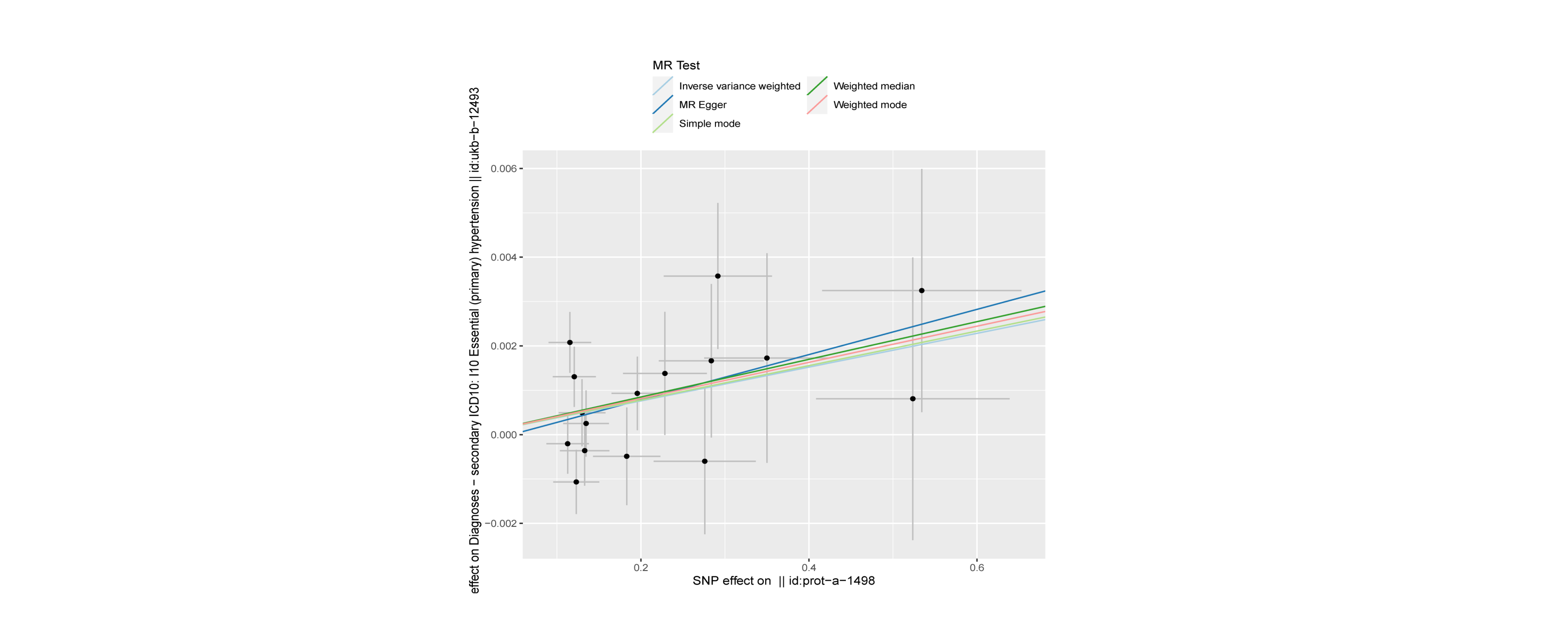
E.**
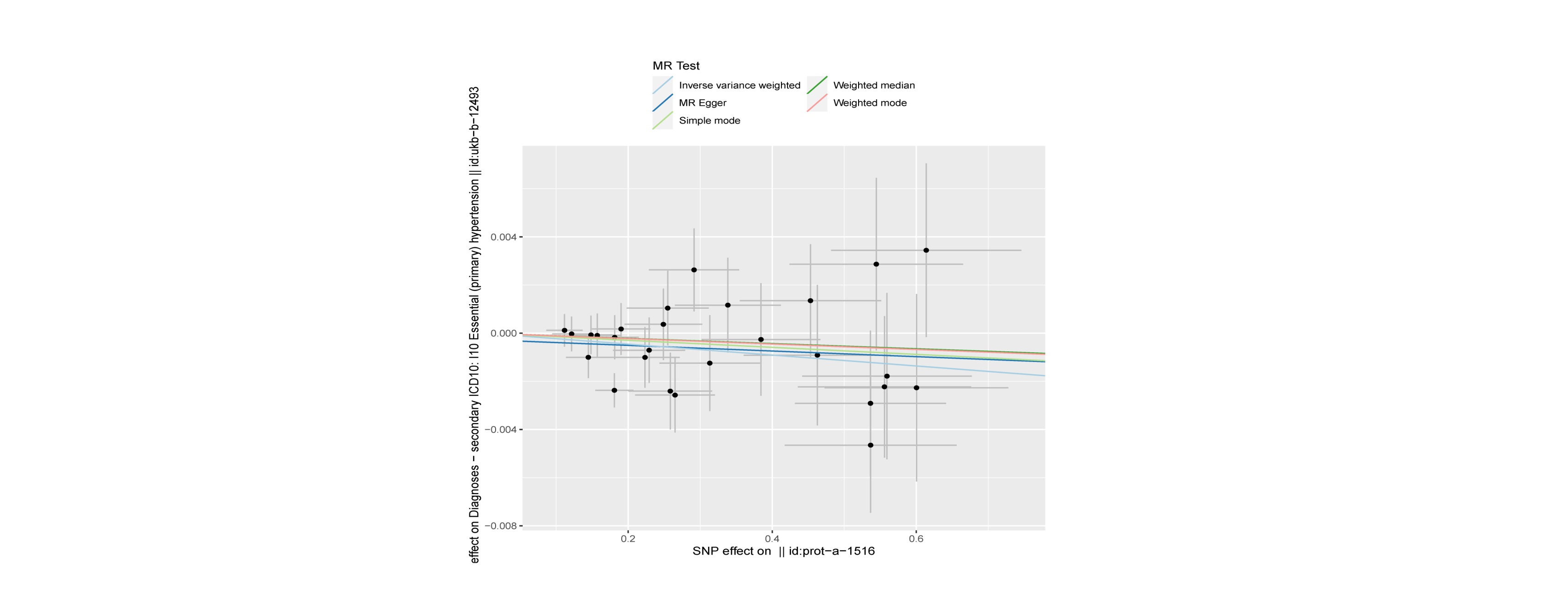
**F.**

**G.**
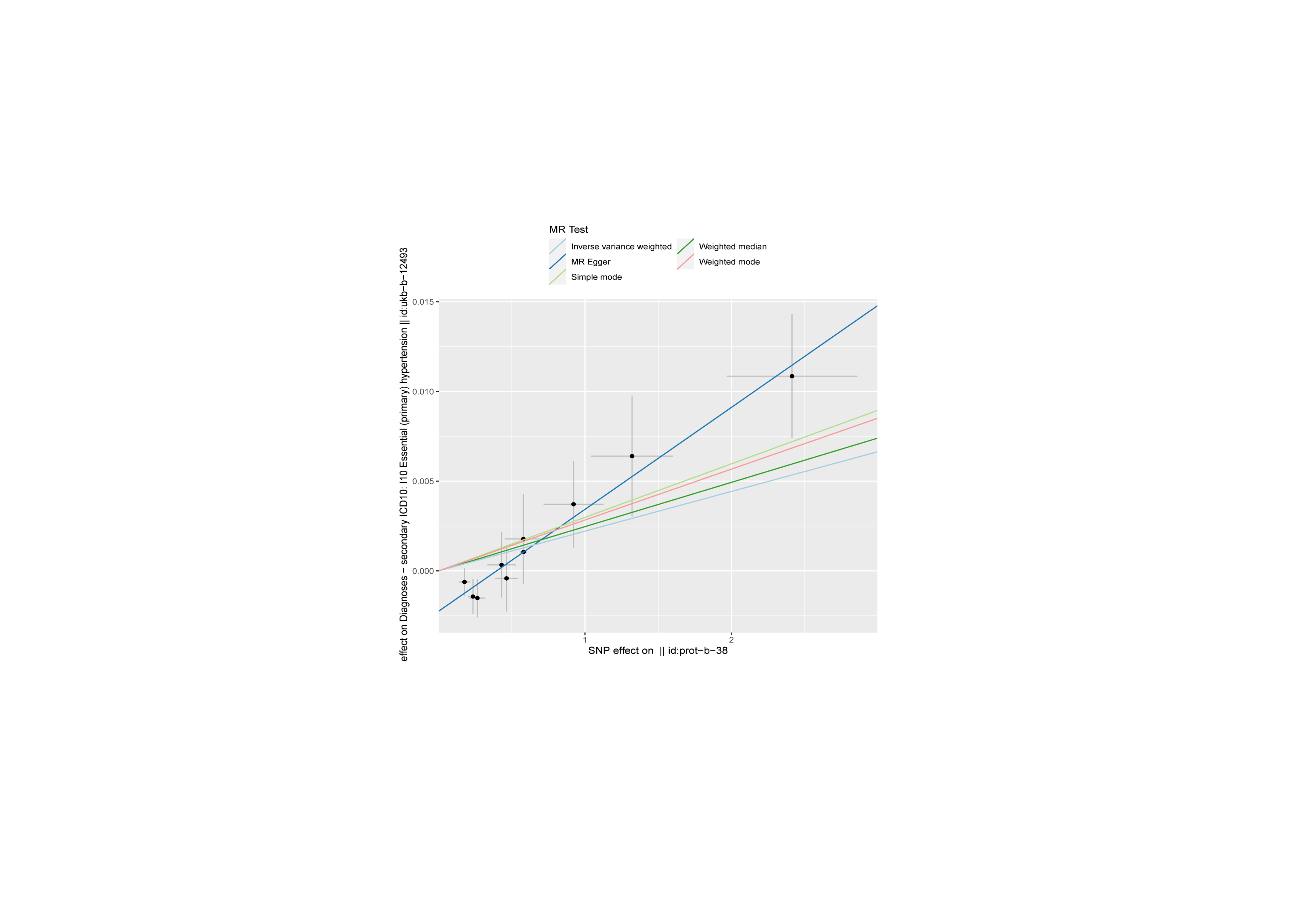


**S9. Leave-one-out analysis of the causal effect of Interleukin-2 receptor subunit alpha levels (A), Interleukin-11 receptor subunit alpha (B), Interleukin-23 (C), Interleukin-1 receptor type 2 (D), Interleukin-27 (E), Interleukin-31 (F) and Interleukin 1 receptor like 1 (G) on hypertension.**

**A.
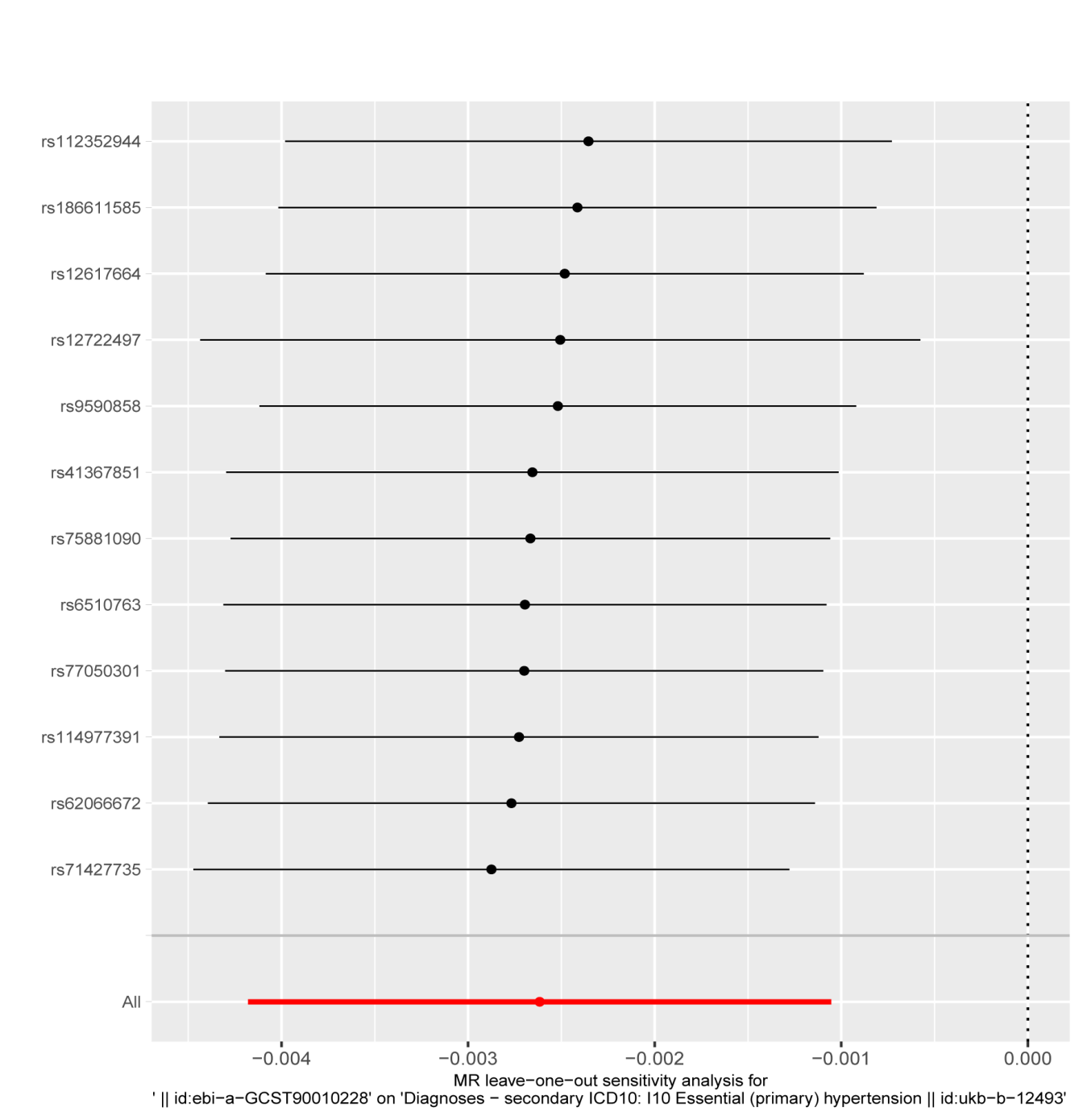
B.
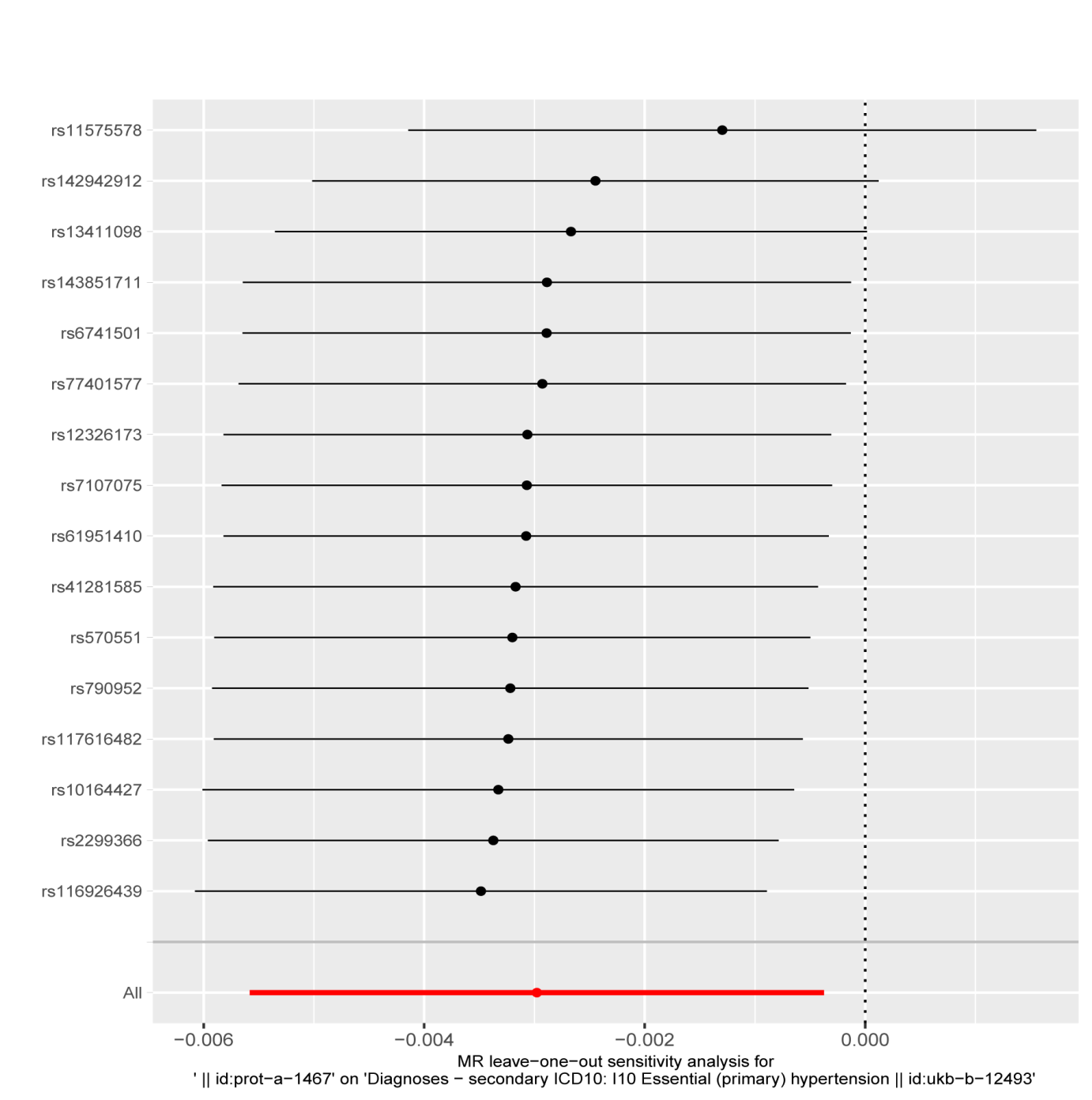
C.
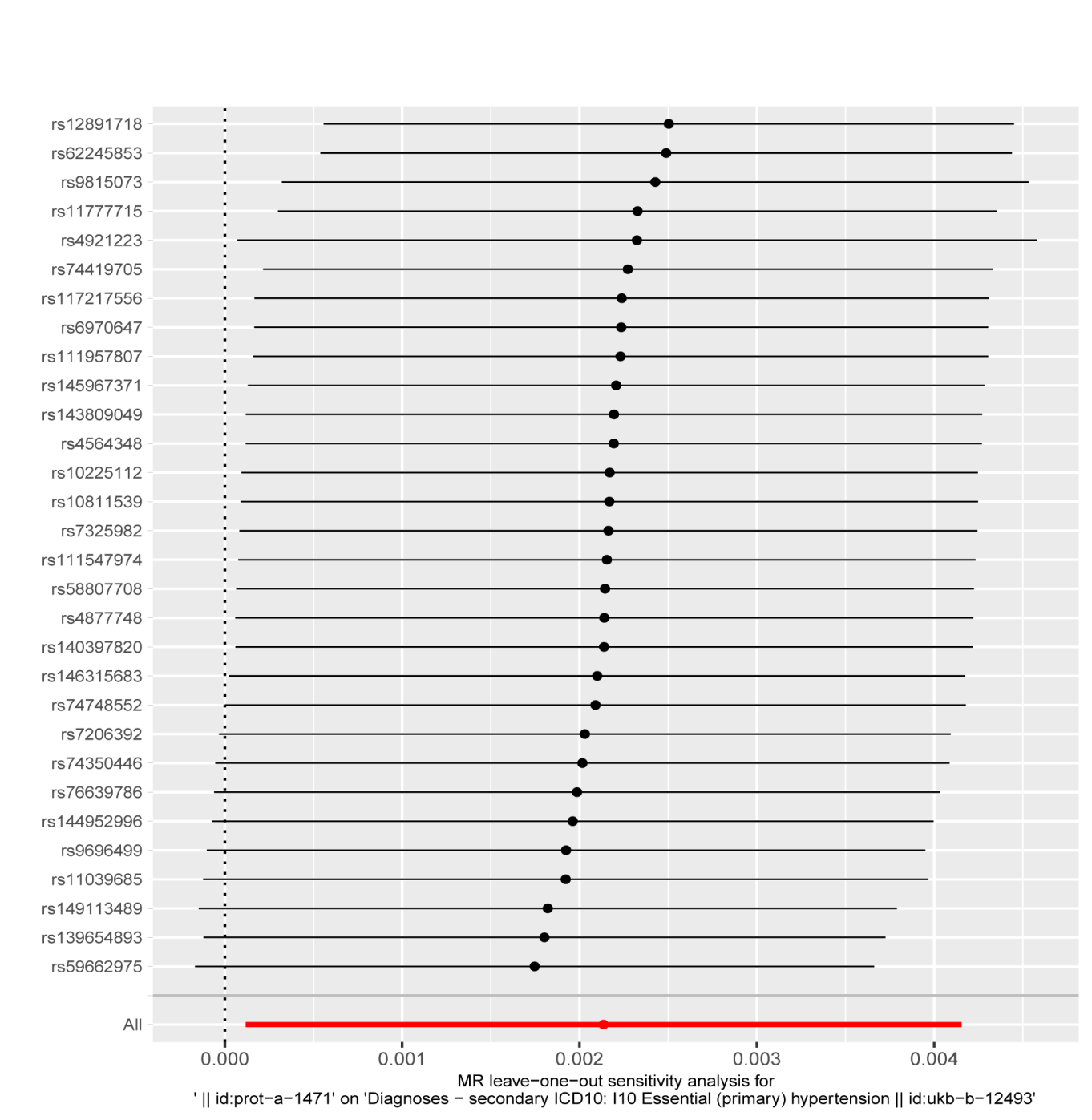
**

**D.
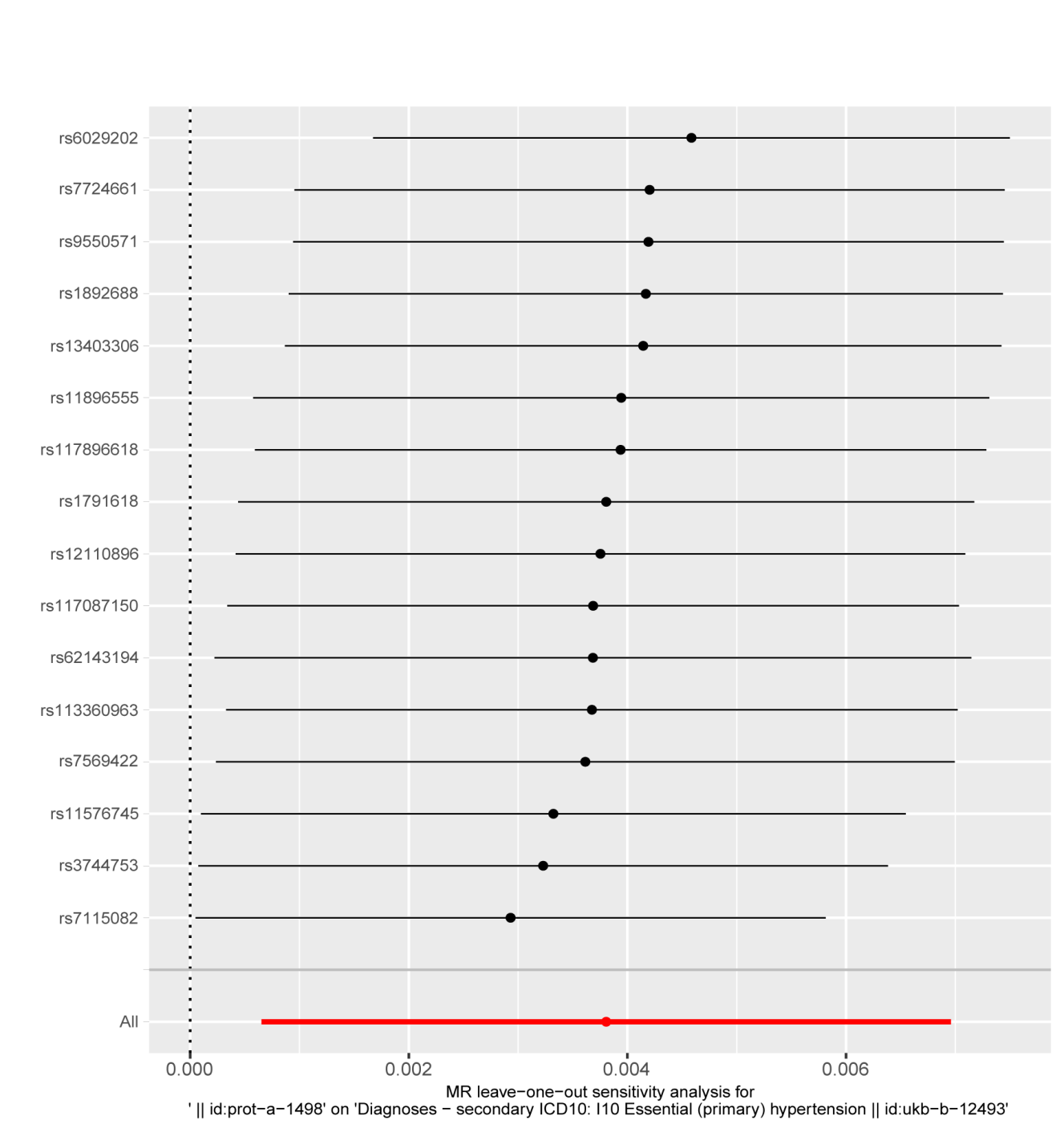
E.
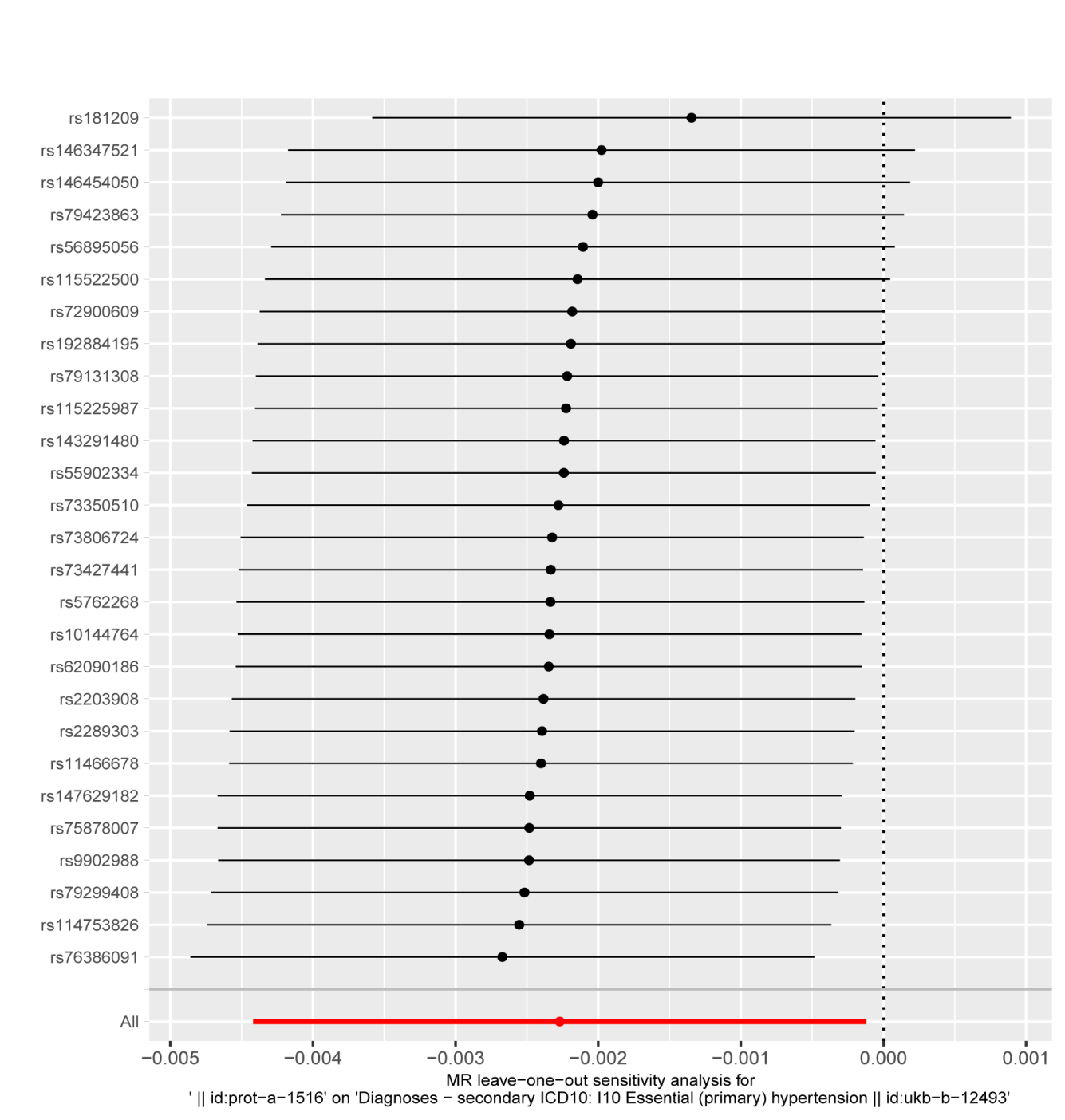
F.
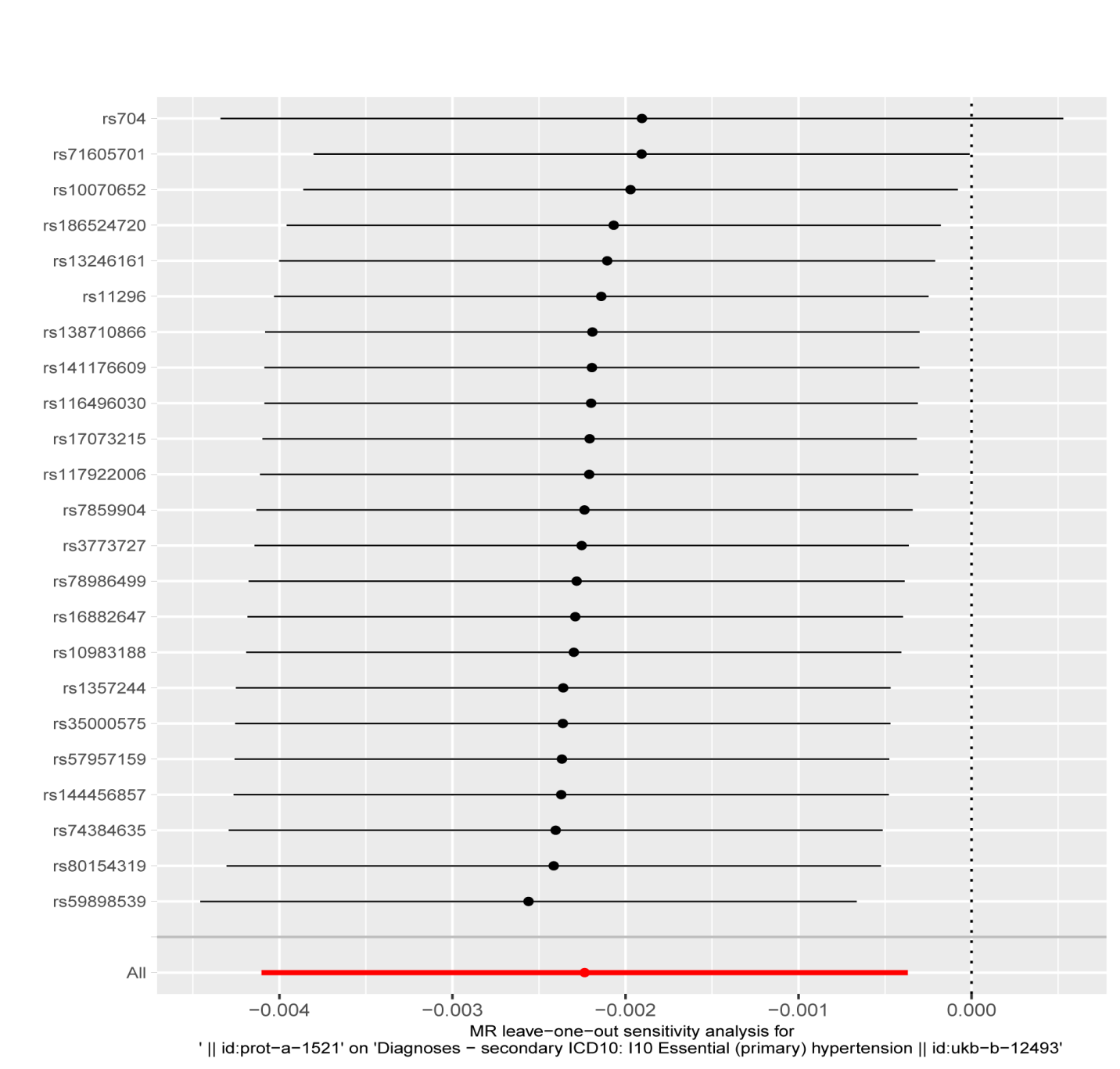
**

**G.
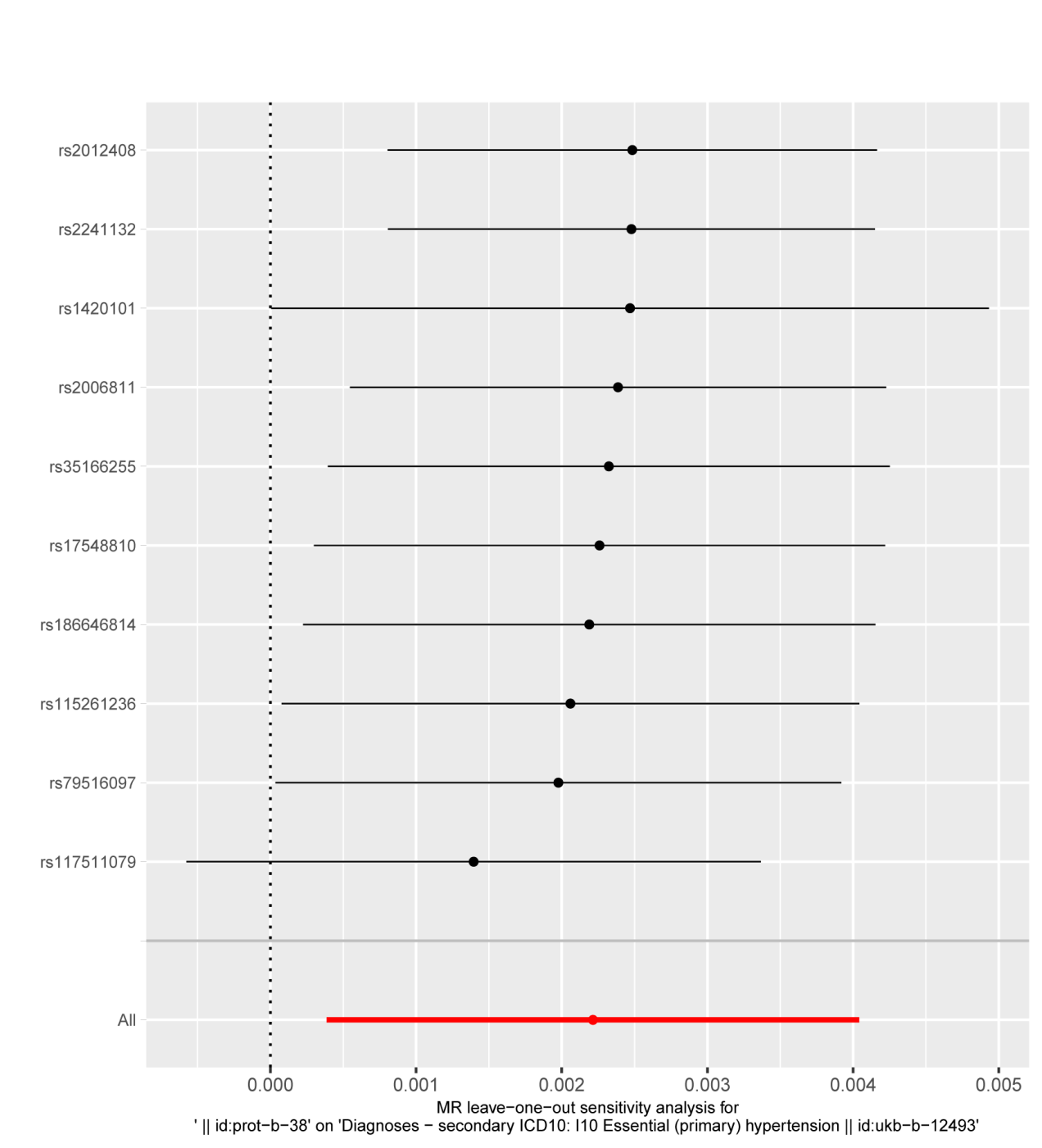
**

**S10. MR results of causal links between interleukin and hypertension.**

| exposure | id.exposure | outcome | id.outcome | method | nsnp | b | se | pval | lo_ci | up_ci |
| --- | --- | --- | --- | --- | --- | --- | --- | --- | --- | --- |
| Interleukin-2 receptor subunit alpha levels | ebi-a-GCST90010228 | hypertension | ukb-b-12493 | MR Egger | 12 | -0.00407 | 0.001742 | 0.041606 | -0.00749 | -0.00066 |
| Interleukin-2 receptor subunit alpha levels | ebi-a-GCST90010228 | hypertension | ukb-b-12493 | Weighted median | 12 | -0.0025 | 0.001112 | 0.024419 | -0.00468 | -0.00032 |
| Interleukin-2 receptor subunit alpha levels | ebi-a-GCST90010228 | hypertension | ukb-b-12493 | Inverse variance weighted | 12 | -0.00262 | 0.000797 | 0.001031 | -0.00418 | -0.00105 |
| Interleukin-2 receptor subunit alpha levels | ebi-a-GCST90010228 | hypertension | ukb-b-12493 | Simple mode | 12 | -0.00144 | 0.001717 | 0.420406 | -0.0048 | 0.001929 |
| Interleukin-2 receptor subunit alpha levels | ebi-a-GCST90010228 | hypertension | ukb-b-12493 | Weighted mode | 12 | -0.00231 | 0.001149 | 0.069913 | -0.00456 | -5.4E-05 |
| Interleukin-11 receptor subunit alpha | prot-a-1467 | hypertension | ukb-b-12493 | MR Egger | 16 | -0.00794 | 0.002643 | 0.009446 | -0.01312 | -0.00276 |
| Interleukin-11 receptor subunit alpha | prot-a-1467 | hypertension | ukb-b-12493 | Weighted median | 16 | -0.00146 | 0.001995 | 0.464319 | -0.00537 | 0.002451 |
| Interleukin-11 receptor subunit alpha | prot-a-1467 | hypertension | ukb-b-12493 | Inverse variance weighted | 16 | -0.00298 | 0.001329 | 0.024997 | -0.00558 | -0.00037 |
| Interleukin-11 receptor subunit alpha | prot-a-1467 | hypertension | ukb-b-12493 | Simple mode | 16 | 0.00083 | 0.003867 | 0.832909 | -0.00675 | 0.00841 |
| Interleukin-11 receptor subunit alpha | prot-a-1467 | hypertension | ukb-b-12493 | Weighted mode | 16 | 0.000958 | 0.003577 | 0.792514 | -0.00605 | 0.00797 |
| Interleukin-23 | prot-a-1471 | hypertension | ukb-b-12493 | MR Egger | 30 | 0.004868 | 0.002444 | 0.056231 | 7.77E-05 | 0.009659 |
| Interleukin-23 | prot-a-1471 | hypertension | ukb-b-12493 | Weighted median | 30 | 0.001212 | 0.001542 | 0.432176 | -0.00181 | 0.004235 |
| Interleukin-23 | prot-a-1471 | hypertension | ukb-b-12493 | Inverse variance weighted | 30 | 0.002136 | 0.00103 | 0.038092 | 0.000117 | 0.004154 |
| Interleukin-23 | prot-a-1471 | hypertension | ukb-b-12493 | Simple mode | 30 | 0.000574 | 0.002675 | 0.831631 | -0.00467 | 0.005816 |
| Interleukin-23 | prot-a-1471 | hypertension | ukb-b-12493 | Weighted mode | 30 | 0.000711 | 0.001943 | 0.716894 | -0.0031 | 0.00452 |
| Interleukin-1 receptor type 2 | prot-a-1498 | hypertension | ukb-b-12493 | MR Egger | 16 | 0.005096 | 0.004181 | 0.243049 | -0.0031 | 0.01329 |
| Interleukin-1 receptor type 2 | prot-a-1498 | hypertension | ukb-b-12493 | Weighted median | 16 | 0.004245 | 0.002097 | 0.04293 | 0.000135 | 0.008355 |
| Interleukin-1 receptor type 2 | prot-a-1498 | hypertension | ukb-b-12493 | Inverse variance weighted | 16 | 0.003806 | 0.001609 | 0.018019 | 0.000652 | 0.00696 |
| Interleukin-1 receptor type 2 | prot-a-1498 | hypertension | ukb-b-12493 | Simple mode | 16 | 0.003891 | 0.00344 | 0.275785 | -0.00285 | 0.010634 |
| Interleukin-1 receptor type 2 | prot-a-1498 | hypertension | ukb-b-12493 | Weighted mode | 16 | 0.004075 | 0.003487 | 0.260817 | -0.00276 | 0.01091 |
| Interleukin-27 | prot-a-1516 | hypertension | ukb-b-12493 | MR Egger | 27 | -0.00117 | 0.002433 | 0.634127 | -0.00594 | 0.003596 |
| Interleukin-27 | prot-a-1516 | hypertension | ukb-b-12493 | Weighted median | 27 | -0.00108 | 0.001528 | 0.47948 | -0.00408 | 0.001915 |
| Interleukin-27 | prot-a-1516 | hypertension | ukb-b-12493 | Inverse variance weighted | 27 | -0.00227 | 0.001096 | 0.038505 | -0.00442 | -0.00012 |
| Interleukin-27 | prot-a-1516 | hypertension | ukb-b-12493 | Simple mode | 27 | -0.00147 | 0.002978 | 0.626258 | -0.00731 | 0.00437 |
| Interleukin-27 | prot-a-1516 | hypertension | ukb-b-12493 | Weighted mode | 27 | -0.00113 | 0.002988 | 0.709192 | -0.00698 | 0.00473 |
| Interleukin-31 | prot-a-1521 | hypertension | ukb-b-12493 | MR Egger | 23 | -0.00265 | 0.002119 | 0.225415 | -0.0068 | 0.001507 |
| Interleukin-31 | prot-a-1521 | hypertension | ukb-b-12493 | Weighted median | 23 | -0.00263 | 0.001389 | 0.058305 | -0.00535 | 9.25E-05 |
| Interleukin-31 | prot-a-1521 | hypertension | ukb-b-12493 | Inverse variance weighted | 23 | -0.00224 | 0.000953 | 0.018922 | -0.0041 | -0.00037 |
| Interleukin-31 | prot-a-1521 | hypertension | ukb-b-12493 | Simple mode | 23 | -0.00229 | 0.002673 | 0.401863 | -0.00752 | 0.002954 |
| Interleukin-31 | prot-a-1521 | hypertension | ukb-b-12493 | Weighted mode | 23 | -0.00257 | 0.001511 | 0.102924 | -0.00553 | 0.000391 |
| Interleukin 1 receptor like 1 | prot-b-38 | hypertension | ukb-b-12493 | MR Egger | 10 | 0.005683 | 0.001226 | 0.001674 | 0.003281 | 0.008085 |
| Interleukin 1 receptor like 1 | prot-b-38 | hypertension | ukb-b-12493 | Weighted median | 10 | 0.002467 | 0.000978 | 0.011669 | 0.00055 | 0.004385 |
| Interleukin 1 receptor like 1 | prot-b-38 | hypertension | ukb-b-12493 | Inverse variance weighted | 10 | 0.002214 | 0.000933 | 0.017578 | 0.000386 | 0.004042 |
| Interleukin 1 receptor like 1 | prot-b-38 | hypertension | ukb-b-12493 | Simple mode | 10 | 0.002984 | 0.001671 | 0.107798 | -0.00029 | 0.006259 |
| Interleukin 1 receptor like 1 | prot-b-38 | hypertension | ukb-b-12493 | Weighted mode | 10 | 0.002837 | 0.001076 | 0.027047 | 0.000728 | 0.004946 |

**S11. Funnel plot of the causal effect of genus Clostridium innocuum group id.14397 on Interleukin-1 receptor type 2.**

**
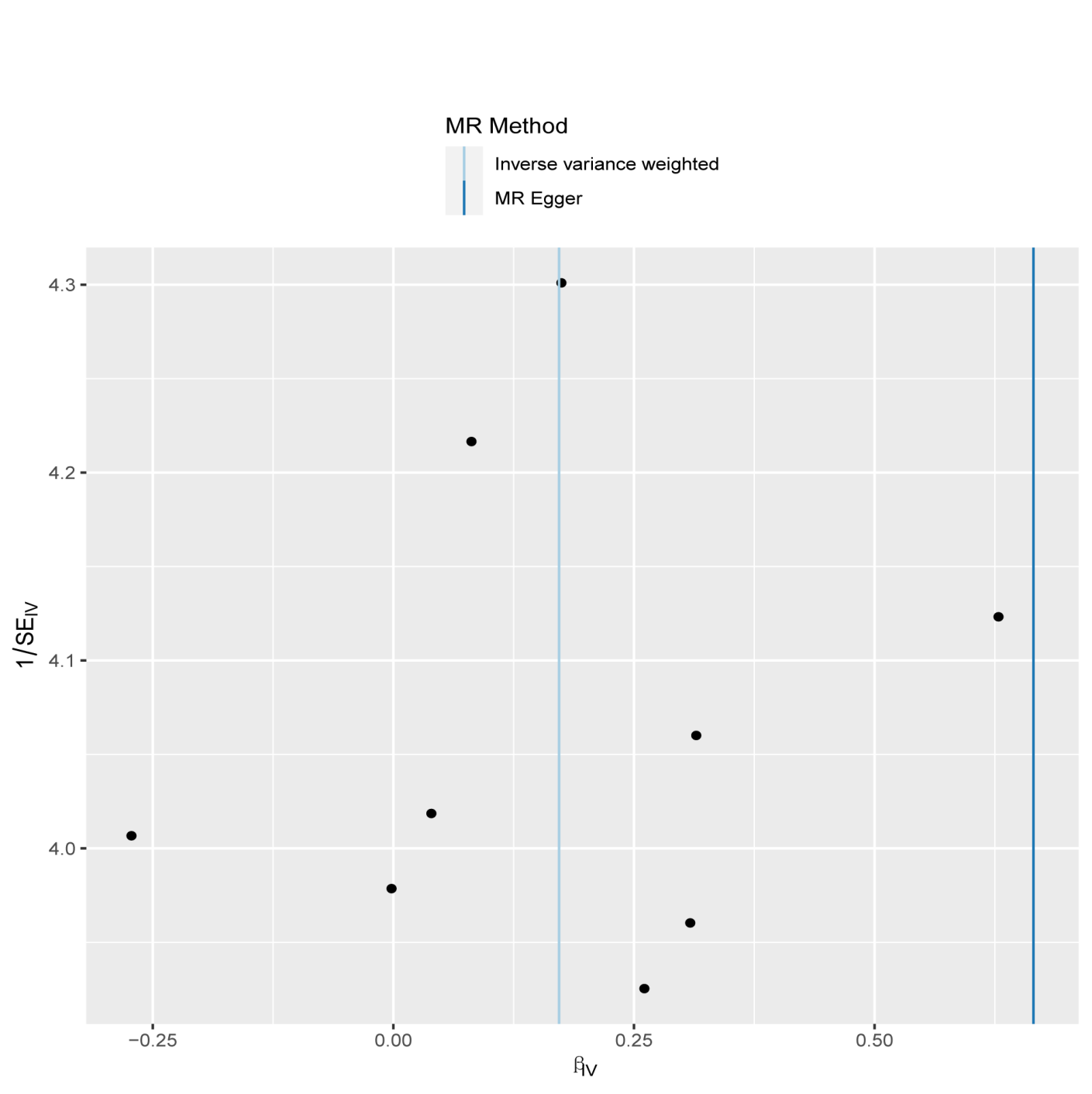
**

**S12. Scatter plot of the causal effect of genus Clostridium innocuum group id.14397 on Interleukin-1 receptor type 2.**

**S13. Leave-one-out analysis of the causal effect of genus Clostridium innocuum group id.14397 on Interleukin-1 receptor type 2.**

**
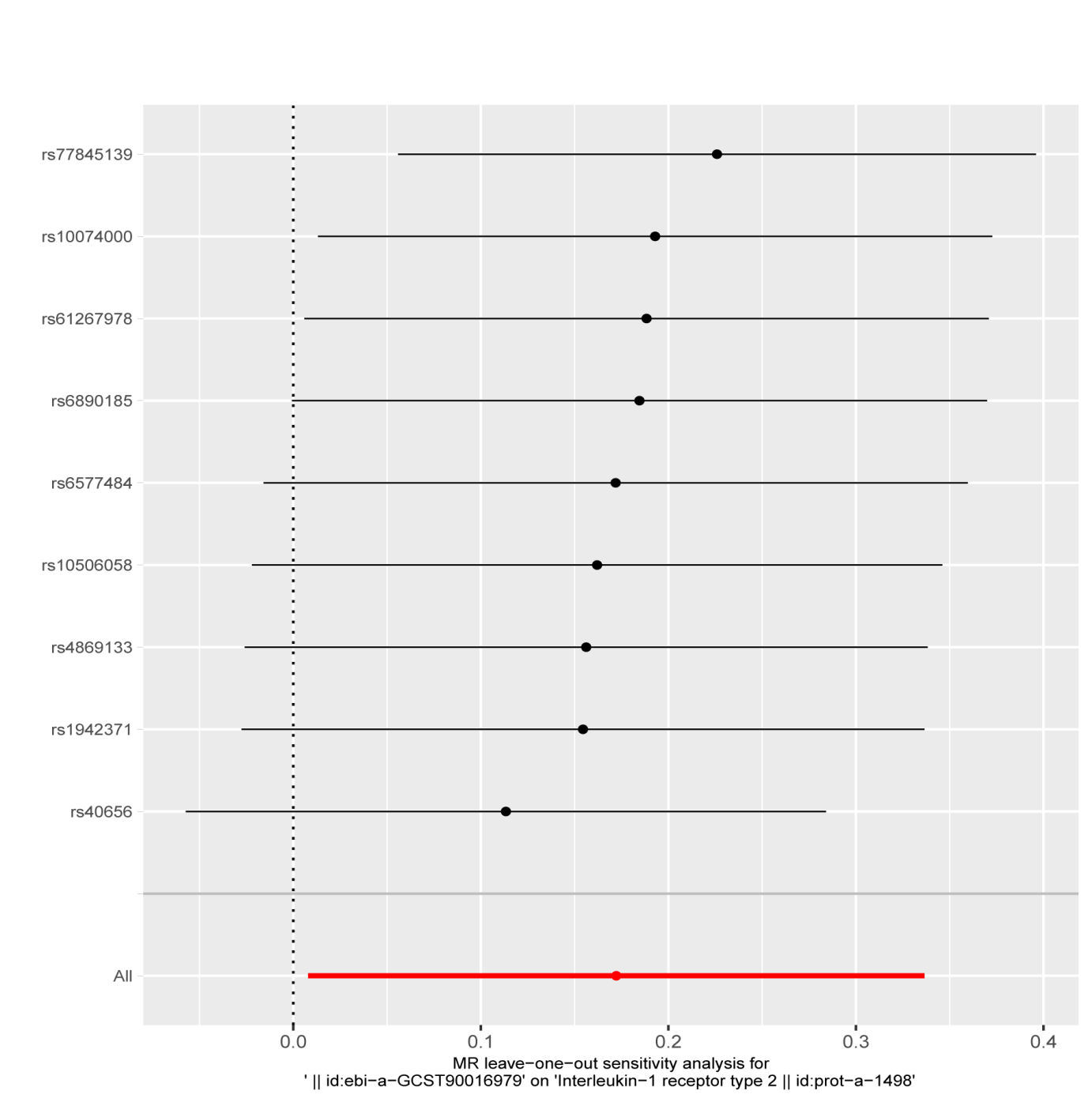
**

**S14. MR results of causal links between genus Clostridium innocuum group id.14397 and Interleukin-1 receptor type 2.**

| exposure | id.exposure | outcome | id.outcome | method | nsnp | b | se | pval | lo_ci | up_ci |
| --- | --- | --- | --- | --- | --- | --- | --- | --- | --- | --- |
| genus Clostridium innocuum group id.14397 | ebi-a-GCST90016979 | Interleukin-1 receptor type 2 | prot-a-1498 | MR Egger | 9 | 0.665042456 | 0.416059189 | 0.153979047 | -0.150433555 | 1.480518467 |
| genus Clostridium innocuum group id.14397 | ebi-a-GCST90016979 | Interleukin-1 receptor type 2 | prot-a-1498 | Weighted median | 9 | 0.155185787 | 0.115254542 | 0.178153775 | -0.070713116 | 0.38108469 |
| genus Clostridium innocuum group id.14397 | ebi-a-GCST90016979 | Interleukin-1 receptor type 2 | prot-a-1498 | Inverse variance weighted | 9 | 0.172206024 | 0.083842777 | 0.039983819 | 0.007874181 | 0.336537866 |
| genus Clostridium innocuum group id.14397 | ebi-a-GCST90016979 | Interleukin-1 receptor type 2 | prot-a-1498 | Simple mode | 9 | 0.189224576 | 0.180754485 | 0.325760167 | -0.165054214 | 0.543503366 |
| genus Clostridium innocuum group id.14397 | ebi-a-GCST90016979 | Interleukin-1 receptor type 2 | prot-a-1498 | Weighted mode | 9 | 0.161080852 | 0.165961508 | 0.360177886 | -0.164203703 | 0.486365407 |
